# A fairness scale for real-time recidivism forecasts using a national database of convicted offenders

**DOI:** 10.1007/s00521-025-11478-x

**Published:** 2025-08-01

**Authors:** Jacob Verrey, Peter Neyroud, Lawrence Sherman, Barak Ariel

**Affiliations:** 1 Institute of Criminology, University of Cambridge, Sidgwick Ave, Cambridge, CB3 9DA UK; 2 Benchmark Cambridge Ltd., Rectory Lane, Somersham, PE28 3EL UK; 3 Institute of Criminology, The Hebrew University of Jerusalem Mt. Scopus, 9190501 Jerusalem, Israel

**Keywords:** Machine learning, Criminal justice, Forecasting, Recidivism, Fairness

## Abstract

This investigation explores whether machine learning can predict recidivism while addressing societal biases. To investigate this, we obtained conviction data from the UK’s Police National Computer (PNC) on 346,685 records between January 1, 2000, and February 3, 2006 (His Majesty’s Inspectorate of Constabulary in Use of the Police National Computer: An inspection of the ACRO Criminal Records Office. His Majesty’s Inspectorate of Constabulary, Birmingham, https://assets-hmicfrs.justiceinspectorates.gov.uk/uploads/police-national-computer-use-acro-criminal-records-office.pdf, 2017). We generate twelve machine learning models—six to forecast general recidivism, and six to forecast violent recidivism—over a 3-year period, evaluated via fivefold cross-validation. Our best-performing models outperform the existing state-of-the-arts, receiving an area under curve (AUC) score of 0.8660 and 0.8375 for general and violent recidivism, respectively. Next, we construct a fairness scale that communicates the semantic and technical trade-offs associated with debiasing a criminal justice forecasting model. We use this scale to debias our best-performing models. Results indicate both models can achieve all five fairness definitions because the metrics measuring these definitions—the statistical range of recall, precision, positive rate, and error balance between demographics—indicate that these scores are within a one percentage point difference of each other. Deployment recommendations and implications are discussed. These include recommended safeguards against false positives, an explication of how these models addressed societal biases, and a case study illustrating how these models can improve existing criminal justice practices. That is, these models may help police identify fewer people in a way less impacted by structural bias while still reducing crime. A randomized control trial is proposed to test this illustrated case study, and further directions explored.

**Supplementary Information:**

The online version contains supplementary material available at 10.1007/s00521-025-11478-x.

## Introduction

### Forecasting recidivism via machine learning

Convicted offenders who recidivate impose serious costs on the UK. First, they impose financial costs. Namely, they are estimated to commit between 434,400 and 556,800 crimes one year after release, which imposes an annual average financial cost ranging between £2.837 billion to £3.630 billion (see suppl. materials for estimations). Second, they impose resource costs. In other words, resources in the criminal justice system are strained [2], and convicted offenders who recidivate may stress these strained resources via lengthy police investigations, victim services, and legal prosecutions [3]. Third, they inflict difficult-to-quantify emotional costs—costs that are borne by victims and communities affected by the crime, as well as those working in the criminal justice system overall [4].

Semina Halliwell may best illustrate the emotional costs of crime: The 12-year-old “changed into a different person” after being raped by a serial rapist, and shortly thereafter, she took her own life [5]. While Semina’s case was extreme, lesser albeit serious variations of emotional costs exist. For example, Ron Evans was ordered to pay compensation to a victim he sexually assaulted in a community center [6]; and Joseph Phillips stole from hundreds of people throughout his criminal career [7]. These offenders have had criminal records. Therefore, if the criminal justice system can predict recidivism, it may be able to target its scarce resources better to prevent these crimes and their serious costs.

For this reason and others, it has attempted to do so for nearly 100 years [8]. Indeed, early recidivism forecasting was typically used to inform parole decisions; they were typically based on unvalidated intuition or simple bivariate associations [9]. They improved over the twentieth century with the introduction of proper evaluation techniques and conventional statistics (e.g., [10]); yet, their results were sometimes underwhelming to the extent where their real-world utility was questionable [11, 12]. Many tools improved substantially with the advent of machine learning [13], in which many practitioners could actualize the predictive validity of these tools, as well as begin harvesting the cost-saving opportunities they produced.

In other words, machine learning is a branch of artificial intelligence in which the computer learns patterns in data [14]. It can produce forecasting tools whose predictive validity vastly exceeds traditional criminological risk assessments based on weighted questionnaires, professional judgment, or conventional statistical techniques. Indeed, machine learning has produced state-of-the-art forecasting tools that can predict domestic homicide, police misconduct, and the spatial–temporal distribution of crime, among other consequential, criminological use cases. When applied to recidivism forecasting, it has produced results that often exceed traditional risk assessments [15–17]. Once developed, these high-performing tools can then be integrated into key decision hubs within the criminal justice system—hubs that include police dispatch centers to help deploy officers to locations likely to experience a serious crime, in the courtroom to facilitate sentencing, and into the parole room to inform a parole decision, among others [18].

However, these machine learning tools need to be carefully developed and deployed to ensure their high performance translates to real-world benefits. In the case of development, machine learning tools use patterns in historical data to predict the future. Thus, if these historical patterns contain uncorrected biases, then these tools risk perpetuating these biases [19]—a common concern for tools trained with policing data [20]. In the case of deployment, measures need to be put in place to combat concept drift—a model’s performance getting worse over time due to changes in the input data [21]—and algorithmic bias, in which a human uncritically acts upon the system’s output [22], among other concerns. Both these development and deployment concerns materialized in the deployment of COMPAS, a recidivism forecasting software that has been accused of perpetuating racial bias and has erroneously kept a low-risk prisoner in jail due to an uncritical reliance on its predictions [22, 23].

### The case of the Police National Computer (PNC)

In short, machine learning tools can predict recidivism with greater predictive validity than conventional risk assessments [13, 15–17], and practitioners can use these predictions to prevent future crime. For example, the Pennsylvania Board of Probation employed a carefully designed forecasting tool that influenced parole decisions, ultimately reducing both violent and non-violent crimes [24]. However, the perfunctory development and deployment of these tools can negatively affect lives. This was exemplified via the Eastern Correctional Facility in upstate New York, which used a tool that exhibited biases and incorrectly denied individuals parole, thereby harming these low-risk individuals and inflicting serious reputational damage on the department using it [22, 23].

**Fig. 1:**
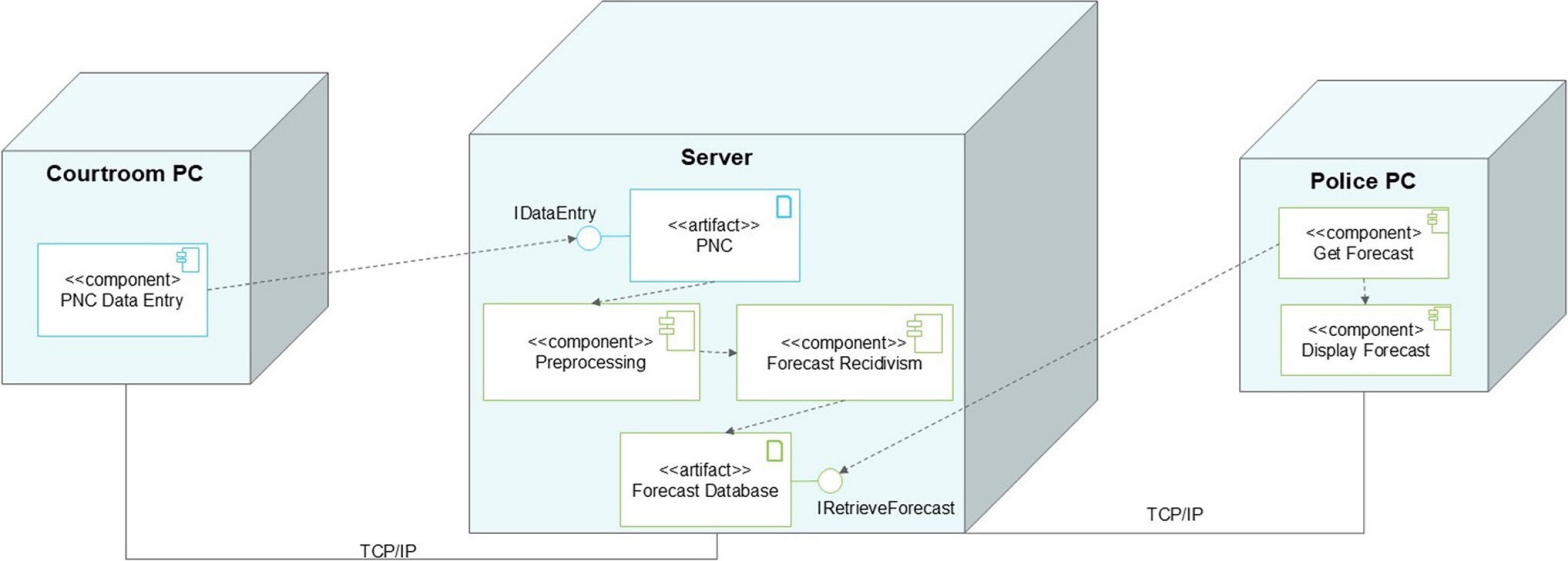
Hypothetical UML deployment diagram. Diagram illustrates potential integration of proposed forecasting instrument into existing criminal justice IT infrastructure. Blue represents existing infrastructure, whereas green represents proposed additions. Diagram illustrates that no new machines or major pieces of IT infrastructure need to be installed to fully deploy our models

To harvest the benefits of machine learning while mitigating its costs, we propose a carefully crafted machine learning tool that can predict recidivism via criminal justice records. Namely, the Police National Computer (PNC) is a centralized database that records convictions and other criminal justice events throughout the UK [1, 25]. In other words, an individual is entered into the PNC each time he/she receives a disposition. We can build a machine learning pipeline upon the existing infrastructure of the PNC, with an illustrative deployment diagram appearing in Fig. 1.

Using this deployment, an offender will receive a recidivism prediction each time he/she receives a conviction. These predictions can then be sent to the law enforcement agency where the convicted offender resides so that police can better manage the offender. Namely, if police receive a positive prediction—i.e., that a convicted offender *will* commit another offense—the officers can enact a variety of interventions to prevent the crime from occurring—interventions such as increased supervision, nudges, or focused deterrence [26, 27]. If police receive a negative prediction—i.e., that a convicted offender will *not* commit another offense—then police can reallocate their resources appropriately such that the offender will not be targeted by law enforcement. Thus, the predictions generated by this model can be used to manage convicted offenders and prevent crime.

Additionally, the criminal justice system is known to exhibit structural or societal biases. Police, for example, may be more likely to target some demographic groups more than others [28]. These structural biases may have persisted for decades, and they may be recreated in predictions generated by a machine learning model trained on criminal justice data [20]. To address these decades-long biases, we propose a fairness scale that provides a framework by which criminal justice practitioners can debias their models. The fairness scale will not remove all forms of societal bias in a model. However, it represents a major and extensive form of bias reduction that is more thorough than measures undertaken by contemporaries (see Sect. 2.2), and it communicates the technical and political assumptions associated with different forms of debiasing. Ultimately, a forecasting model debiased via the fairness scale should produce recidivism predictions that reflect less structural bias than the status quo, which can lead to police acting on these recommendations—that is, reallocating their resources—in a way that is more equitable.

### Overview

To carefully craft this tool, we first review the extant literature on machine learning models that forecast recidivism. We use these studies to define a common set of parameters by which we construct our model—practices that ensure both rigor and comparability with other state-of-the-art tools. Next, we review the fairness literature as it relates to addressing historical biases within machine learning data. We use that literature to construct a scale that communicates the semantic and technical trade-offs associated with debiasing a model. Finally, we integrate findings from both literature reviews—the common parameters and fairness scale—to establish the foundation for our investigation. Namely, we develop general and violent recidivism forecasting models that surpass the current state-of-the-art, then we debias them such that they implement each fairness definition. Deployment considerations and suggested next steps are discussed.

### Statement of contributions

Ultimately, this investigation made theoretical, methodological, and application-related contributions. Namely, the following theoretical contributions are made:

- Extract a common set of parameters by which forecasting models are constructed. This extraction required resolving ambiguities in the literature such as conflicting recidivism definitions. The extraction of these parameters renders them accessible and easy-to-implement by others designing similar forecasting instruments (Sect. 2.1).
- Propose a fairness scale that communicates the technical and semantic challenges of debiasing a model. This is crucial because fairness definitions are seldom implemented in forecasting and criminal justice, and if they are, technical and semantic trade-offs implied therein are sometimes limited (see Sect. 2.2). The fairness scale provides a concise set of fairness definitions highly relevant to criminal justice practitioners, which facilitates the adoption of fairness definitions that are otherwise unimplemented.

The following methodological contributions are made:

- Showcase how others can develop state-of-the-art models that surpass existing forecasting instruments. This was accomplished by using an extensive dataset that is routinely used in the UK national criminal justice process, which involved using preprocessing techniques to render this dataset conducive to machine learning even though it was never intended for this purpose. This was also accomplished via a novel combination of preprocessing techniques that produce predictors inspired by criminological theories, among others (see Sect. 3.3).
- Showcase how others can create a debiased model that has implemented a more extensive form of bias correction than any study reviewed in Sect. 2 (see Sect. 5.3.1 for claim). This was accomplished through a combination of pre- and post-processing techniques suggested by the fairness scale, such as a novel implementation of maximizing performance under fairness (Sect. 3.7).

From an application perspective, recommendations are made that bridge the gap between machine learning, fairness research, and criminal justice:

- Suggest how predictions generated via a debiased forecasting model can improve existing criminal justice practices (see Sect. 5.3.2).
- Propose a randomized control trial to measure real-world impact of those predictions (see Sect. 5.4).
- Propose safeguards to limit the harm of false positives when the model is used in practice (see Sect. 5.1).

## Literature review

Two literatures are reviewed. First, we review the literature on extant machine learning tools that forecast recidivism to define a set of common parameters that will guide model constructions. Second, we review the literature on fairness in machine learning so that we can partially address the historical biases found within our dataset.

Clarifying terms is essential before conducting the literature review, and this is especially true for machine learning, which is complicated by differing terminologies used in the statistics and computer science literature to describe the same underlying phenomena [29]. Thus, in this investigation, we use terms specific to computer science. In terms of the dataset, the term *features* refers to the covariates or independent variables that the model uses to make a prediction, whereas the term *label* refers to the dependent variable or outcome, the phenomenon that the model attempts to predict. Moreover, machine learning *algorithms* are equations or computer code used to output *models*, which are the ultimate *tools* that practitioners can use to forecast recidivism. Finally, a model can either output a probability that a convicted offender will recidivate or a binary yes/no. In the case of binary classification, however, practitioners typically do not classify offenders into “will recidivate” or “will not recidivate” bins—they typically use the terms “high risk” and “low risk,” respectively. Thus, to map the output of a binary classification to law enforcement terminology, we will treat the denotations “high risk” and “will recidivate” as interchangeable, with the same logic applying to “low risk” and “will not recidivate”. Finally, the terms “debias” and “implement fairness” will be treated as synonymously, as advocated in Sect. 2.2.1. A summary of these terms appears in Table 1.

**Table 1:** Glossary of terms, grouped by category

Term	Definition
*Dataset*	
Features	Covariates or variables in a dataset. These are what a model is using to predict recidivism
Label	The ultimate phenomenon a model is trying to predict, such as recidivism over a 3-year period
*Model construction*	
Algorithm	Computer code and/or equation(s) used to learn underlying patterns in data. Outputs a model
Model/tool	A set of rules that are used to make predictions about the future
*Output*	
Predict/predictions	Treated as interchangeable with forecast/forecasts
Will recidivate	Treated as synonymous with “high risk”
Will not recidivate	Treated as synonymous with “low risk”
*Other*	
Debias/fairness	“Debiasing” a model is treated as synonymous with “implementing fairness”

### Recidivism forecasting

To conduct a review on extant machine learning forecasting instruments, we draw from a recent literature review that enumerates twelve different studies that each validated a machine learning forecasting tool [17]. We reviewed each of these studies one-by-one to extract key information. The general result of our review appears in Table 2, with further results appearing in Tables 3 and 4. Note that one of the twelve studies was omitted because the relevant portion of the study was similar to an earlier equivalent.

**Table 2:** Review of machine learning studies that forecast recidivism: definitions and dataset characteristics

Study	Definition(s)			Population		Dataset	
	Unit	Event	Forecast Length	Description	Years (sample size)	Name	Contents (illustrative)
*General recidivism*							
[13]	Convictions	Reconviction for *any offense* that is detected through the Minnesota Bureau of Criminal Apprehension [68]	Indefinite	Offenders in Minnesota, USA	2003–2010 (27,772)	MnSTARR +	55 items over 4 categories: criminal history, demographics, intake, & dynamic factors
[15]	Convictions	Any criminal offense for which an individual is returned into the Ontario Ministry of Community Safety and Correctional Services system on a reconviction, sentenced to incarceration or community supervision	3 years	Offenders in Ontario, Canada with ≤ 2 years sentence	2010–2011 (72,725) and 2004 (26,450)	LS/CMI	43 items over 8 domains: criminal history, education/employment, family/marital, leisure/recreation, companions, substance abuse, pro-criminal attitudes, and antisocial patterns
[35]	Arrests	Any arrest after initial arrest	Indefinite	Probation volunteers in Texas, USA	2017–2019 (730)	NCRA +	7 psychological tests that collectively contained 48 items and demographics
[38]	Anything causing one to end up in a “prison” or “special home” in India	Defined as “Recurrence in criminal behavior,” though never explicitly defines what constitutes criminal behaviors	Indefinite	First time Offenders in various prisons and special homes in Ranchi, India	Unreported (204)	HCR-20 +	55 items over 7 categories: personality factors, family/parental factors, environmental factors, sentencing context, a psychological evaluation, clinical items, risk items
[16]	Convictions	Reoffending after a prior contact with the criminal justice system, with reoffending defined as being found guilty after undergoing a process of formal adjudication	Indefinite	Youth offenders aged 12–19 in Singapore	2004–2008 (3744)	YLS/CMI	Many items over 8 domains: prior and current offenses, family circumstance/parenting, education/employment, peer relations, substance abuse, leisure/recreation, personality/behavior, attitudes/orientation; demographics
*Violent recidivism*							
[32]	Arrests	Uses the Structured Assessment of Violence Risk in Youth (SAVRY) definition of violent reoffending, which is “an act of physical battery sufficiently severe to cause injury that would require medical attention, a threat with a weapon in hand, or any act of forcible sexual assault” [69]	5 years	Youth offenders in Catalonia, Spain	2010 (855)	SAVRY +	Demographics; 40 items over 4 categories including a final expert evaluation: risk items, protective factors, risk factors, and summary scores
*Sexual recidivism*							
[33]	Charged with sexual offense	Not Explicated. Assumed to be set by Florida Department of Juvenile Justice	2 years	Youth offenders In Florida, USA	2007–2012 (3061)	FDJJ	126 item risk assessment producing 553 predictor variables, trimmed to 336
*Drug-related recidivism*							
[34]	Convictions	Not explicated. Assumed to be a reconviction that leads to returning to the Thailand central correctional institution for drug addicts	Indefinite	Drug addicts in Thai correctional institute	Unreported (598)	Thailand	35 items describing criminal history, financial problems, mental health, social network, feelings, and others
*General and violent recidivism*							
[40]	Convictions	General—any type of reoffending Violence—never explicates what offenses count as violent, but defines 4 types: self-directed violence, violence in prisons, further violent offenses, breaking prison permits	2 years	Offenders that entered prison in Catalonia, Spain whose nationalities were recorded	Entered prison 1989–2012 and were given RiskCanvi assessment between 2010–2013 (2027)	RiskCanvi	Two versions: abbreviated 10-items and 43-item screening. Both contain 5 categories: criminality, biography, family/social, clinical, attitudes/personality
[30]	Convictions	General—any new sentences of any kind (including violent offenses) Violence—homicides and assaults	Various	Male offenders in Finland	2007–2011 (1492)	RITA +	Demographics and 52 RITA items in the following categories: alcohol problems, resistance to change, employment problems, problem managing one’s economy, aggression, and drug abuse and associated behavior
*General, violent, and sexual recidivism*							
[31]	Convictions	General recidivism—all offenses excluding misdemeanors. This includes motoring offenses like leaving the scene of an accident and driving under the influence of alcohol or drugs. C Violent recidivism includes threatening, extortion, property crime by using violence and/or threatening, assault and battery, murder, homicide and culpable homicide but excludes destruction of property Sexual recidivism includes rape, violation, indecent assault (with minors or subordinates), sexual contact below age 16 years, age 12 years and sexual contact with someone unconscious or legally incapable. Excludes indecent exposure and bigamy	4 years	All guilty offenders in Netherlands. Testing size was capped at 10,000 due to memory limits	2005: general (159,298) violent (25,041) 2005–2006: Sexual (1332)	StatRec	General: Demographics (gender, age, age of first conviction, most serious offense type, country of birth) Violence and sexual: Same as general with inclusion of offense type and criminal history

Review is based on Travaini et al. (2022). Studies are grouped by the type for recidivism they predict

**Table 3:** Review of machine learning studies that forecast recidivism: evaluation methods and overall performance scores

Study	Evaluation method (test size (*n*) or folds (*k*))	Major evaluation metrics	AUC
*General recidivism*			
[13]	TS (*n* = 6677)	AUC, ACC, Precision, Recall	.78
[15]	CV (*k* = 10)	AUC, ACC	.75
[35]	CV (*k* = 10)	ACC	.70
[38]	CV (*k* = 5)	ACC, Precision, Recall	N/A
[16]	TS (*n* = 1498)	AUC	.69
*Violent recidivism*			
[32]	CV (*k* = 10)	AUC, DP, ERB	.71
*Sexual recidivism*			
[33]	TS (*n* = 765)	AUC	.71
*Drug-related recidivism*			
[34]	CV (*k* = 10)	ACC, Precision, Recall, * F*_1_	N/A
*General and violent recidivism*			
[40]	CV (*k* unreported)	AUC, GFNR, GFPR	.73 (general) .76 (violent)
[30]	CV (*k* = 5)	AUC, pseudo-R2	.82 (general) .77 (violent)
*General, violent, and sexual recidivism*			
[31]	TS (*n* = 10,000)	AUC, ACC, RMSE, SAR, and two calibration metrics	.78 (general) .74 (violent) .73 (sexual)

Review is based on [17]. Studies are grouped by the type for recidivism they predict

TS, Test set; CV, k-fold cross-validation; AUC, Area under the receiver operator characteristics curve; ACC, Accuracy; DP, Demographic parity; ERB, Error rate balance; GFPR, Generalized false positive rate; GFNR, Generalized false negative rate; RMSE, Root-mean squared error

**Table 4:** Review of machine learning studies that forecast recidivism: algorithms and their prevalence

Algorithm/algorithm family	Prevalence (best performing)	Studies
Generalized linear model	7 (5)	[13, 30–32, 34, 35, 40]
Random forest	7 (3)	[13, 15, 16, 30, 32, 33, 35]
Support vector machine	7 (0)	[13, 15, 31, 32, 34, 35, 40]
Boosting	3 (1)	[13, 31, 35]
Neural networks	6 (1)	[13, 31, 32, 34, 38, 40]
Decision trees	3 (0)	[13, 15, 34]
K-nearest neighbors	5 (0)	[31, 32, 34, 35, 38]
Linear discriminant analysis	2 (1)	[31, 35]
Naive Bayes	4 (0)	[13, 32, 34, 38]
Other	3 (1)	[13, 31, 38]

Table depicts algorithms used to create models that forecast recidivism. Prevalence indicates the number of studies that used the corresponding algorithm at least once, whereas parenthesis indicates the number of studies in which that model performed best. Studies were selected via a recent literature review [17]. Generalized linear models include logistic regression, Elasticnet, and other variants. Boosting includes algorithms that primarily rely on boosting, like a gradient boosting machine or XGBoost. Neural networks include both shallow neural networks (e.g., perceptron) and deep learning frameworks

We now wrestle with this literature to learn common parameters surrounding defining recidivism (Sect. 2.1.1), initializing a dataset to train the algorithms on (Sect. 2.1.2), initializing an evaluation method and metrics (Sect. 2.1.3), and deciding which algorithms to train (Sect. 2.1.4).

#### Defining recidivism

Table 2 reveals major inconsistencies between these studies, suggesting there is little standardized set of common parameters by which forecasting models are constructed. Perhaps the most jarring inconsistency is that these studies disagree on the type of recidivism that is forecasted. Specifically, the studies first disagree on the time horizon: roughly half of the studies forecast recidivism indefinitely (*n* = 5)—measuring whether the individual will recidivate *at some undefined point* in the future—whereas the remainder forecast recidivism between 2 and 5 years into the future. In our conversations with potential stakeholders, we found that the indefinite timespan is undesirable due to its imprecision; and a longer timespan was not preferred because police are typically largely interested in preventing crimes in the near future, not in five or ten-plus years. Thus, we elected to forecast recidivism 3 years into the future. This 3-year timespan was initialized somewhat arbitrarily; however, it falls within the range of other studies, avoids the indefinite timespan used by contemporaries, and is sufficiently short that law enforcement remains interested in its predictions.

Next, the studies somewhat disagree on which offenses to forecast. Specifically, the studies are united in that most forecast general recidivism (*n* = 7), or whether the offender will commit *any* offense at some point in the future. While general recidivism is useful, it includes serious offenses in addition to less serious ones. Practitioners, however, would also be interested in forecasting more serious offenses like violent offenses—which some studies have attempted (*n* = 4). However, all four studies have different and somewhat arbitrary definitions of what counts as a violent offense. For example, [30] narrowly defines violence offenses as a homicide or an assault [31]; labels threats and certain property crimes as violent offenses; and [32] takes a broader approach by incorporating sexual offenses, which have typically received their own specialized forecasting instrument [31, 33]. Moreover, none of these studies has published codebooks or tables that elucidate the specific offenses they labeled as violent, which makes their replication problematic. For example [31], labels certain subtypes of property crimes as violent offenses, and it is difficult to identify which property crimes count as violent without a codebook.

Given the ambiguity within the literature, we adopted a balanced definition for classifying violent offenses. Specifically, we draw from the narrow definition in [30] in that we label obviously violent offenses like assault and homicide as violent. However, we expand it by incorporating elements of the definition within [31] such as labeling serious threats as violent—such as possessing a handgun with the intent of using it—and robbery. Thus, our final definitions of recidivism appear below:

- General recidivism is defined as any reconviction within a 3-year period.
- Violent recidivism is defined as a reconviction for a violent offense within a 3-year period. Violent offenses include homicide, robbery, threats, and assault.

#### Dataset initialization: justifying the PNC and its sparsity

Table 2 suggests that most studies use extensive databases that contain an intimate cross section of a convicted offender’s life—including details like mental health records and employment history (e.g., [30, 34]). These intimate features are required by these models to produce recidivism forecasts, as they likely bolster the predictive validity of these models. However, for a model such as ours—one that can be built upon existing criminal justice infrastructure—we do not have access to this rich dataset due to privacy laws and ethical concerns. Moreover, many models use clinical features to generate predictions, meaning they require a practitioner to assess an offender before a recidivism prediction is generated. While these clinical features are likely useful, it is not practical to have an expert assess each newly convicted offender within the UK. Moreover, the effective use of clinical assessment in machine learning requires consistency—both between expert assessors and within the same expert assessor over time. We did not want to bake these consistency concerns into our model, providing further justification for the exclusion of clinical features within our datasets.

When examining criminal justice datasets within the UK, the Police National Computer (PNC) stood out. The PNC dataset is idiosyncratic to past work in that it is extremely sparse—it contains just a fraction of the features used in other models. Despite this major obstacle, we have chosen it anyway for two reasons. First, the dataset is geographically and temporally vast: Its records span decades, and it contains every conviction record within England and Wales. This will enable the construction of a comprehensive history of an offender’s convictions, overcoming limitations of previous databases, which were geographically restricted—typically confined to a county or state—and temporally limited, often covering only a few years and excluding offenses committed elsewhere.

Second, the PNC was chosen because it is already routinely used by the British criminal justice system. Thus, if we could generate a machine learning model on top of existing PNC infrastructure, it can be deployed within the criminal justice system without majorly changing existing IT infrastructure (see Fig. 1): Practitioners would use existing infrastructure to input data into the PNC, predictions would be generated on the same server as the PNC, and its predictions can be displayed upon computers already installed in police stations, in addition to other use cases. Moreover, predictions would be generated automatically, meaning practitioners do not need to undertake any additional data input or transformation operations. This ease of integration gives rise to the potential impact of this project, as it can quickly affect the entire criminal justice system when the time comes to deploy it—pending further validation of our models (see discussion).

The PNC also contains an additional benefit: It uses conviction data rather than rearrest as the basis for recidivism predictions. In other words, arrest data are notorious for containing police bias—especially if it is garnered through proactive policing [20]. Training a machine learning model on arrest data, therefore, would replicate police biases in who they arrest—and this is especially problematic if police incorrectly arrest certain groups of people who are ultimately found not guilty. In contrast, a conviction dataset requires a very high bar of evidence for entry: A suspect is required to be found guilty by the judicial system of England and Wales in order to appear in our dataset. This high bar of evidence suggests that the people who do recidivate actually did, and that those who were wrongfully arrested or stopped by police would be omitted from our dataset. Of course, using conviction data is not a silver bullet that liberates a model from all forms of bias—the entire judicial process could exhibit human bias—however, this decision implements a major form of bias check that is not present in recidivism studies that use arrest data (e.g., [32, 35]).

#### Initializing evaluation method and metrics

Table 3 contains a list of evaluation methods and metrics that each study uses. The studies usually use a cross-validation procedure (*n* = 7) to validate their models, with the remainder using a train–test split (*n* = 4). This dichotomy reflects the trade-off between the two: Cross-validation is far more computationally expensive, but it provides a better sense of the model’s error rate when compared to the computationally-cheaper train–test split [36]. Due to computational resources not being an issue, we have, therefore, elected to use the more rigorous cross-validation procedure to evaluate our models.

The studies are less consistent with the evaluation metrics used, with one exception: the area under the receiver operator characteristics curve (AUC) score. This score is usually used as a proxy for overall performance in the forecasting literature [37], and for this reason, it is used across virtually every study. Because it is the common evaluation metric that unifies these studies, we will use it so that we can benchmark our model against other state-of-the-art instruments. However, we will also calculate other relevant scores that will qualify how the model’s predictions are used in practice (see methods).

#### Algorithm selection

Table 4 lists the algorithms used to construct the machine learning models presented in each study in Table 2. When choosing an algorithm, most studies take a trial-and-error approach in that they apply a popular assortment of algorithms to a recidivism dataset and choose the one that outputs the best-performing model. Of the minority of studies that do justify their assortment of algorithms [13, 15, 16, 31], they use largely the same algorithms as the trial-and-error studies; hence, no meaningful difference emerges.

Drawing from these studies, we will use the algorithms that have produced the best-performing model across studies as determined by AUC score. In other words, if at least one study has purported that a particular algorithm produced the highest-performing model, we will use that algorithm in the present investigation. Using Table 4, the algorithms that we will implement are a random forest, two variations of an algorithm that outputs a generalized linear model, a boosting algorithm, a neural network, and linear discriminant analysis. Note that [38] purports that their best-performing model is a custom ensemble technique; however, the investigation does not fully describe how they implemented that technique; hence, it cannot be replicated.

Details behind the implementation of each algorithm appear in the Methods. A justification of the relevance of each algorithm appears below.

- *Random forest* is an ensemble learning method composed of multiple decision trees, where each tree is trained on random subsets of data and predictor variables [15]. By aggregating the predictions of individual decision trees, the random forest improves its predictive validity, reduces overfitting, and enhances its robustness [13, 16]. The random forest further combats overfitting via bagging (bootstrap aggregation), where each tree is trained on a randomly resampled dataset, introducing variation. The final prediction is typically determined through a majority voting system. When applied to recidivism, a random forest is particularly useful due to its ability to handle complex, high-dimensional data while combatting against overfitting, thereby achieving acceptable predictive validity.
- *Boosting* is an ensemble learning method designed to enhance predictive accuracy by sequentially improving weak learners [13, 31]. Boosting focuses on refining models by assigning greater weight to misclassified observations in each iteration. This process iteratively strengthens weak classifiers, effectively transforming them into a more robust predictive model. In the context of recidivism prediction, boosting is particularly valuable because it may enhance the model’s propensity to correctly classify high-risk individuals who are typically misclassified due to their scarcity.
- *Linear discriminant analysis* is a classical method for classification that can also incorporate dimension reduction [31, 39]. It projects the data onto a lower-dimensional space while maximizing class separability by computing discriminant functions that optimize the ratio of between-class variance to within-class variance. This results in a linear decision boundary, making LDA particularly effective in scenarios where class distributions can be separated using a hyperplane. In the context of recidivism forecasting, LDA can simplify a range of risk factors, such as age and prior convictions, such that they fit on a simple Cartesian plane, enabling output visualization. Moreover, LDA can offer relatively high stability and reduce overfitting in relatively high-dimensional datasets such as those used in recidivism forecasting.
- *Generalized linear models (GLMs)* encompass a family of models that extend traditional linear regression to handle different types of response variables [39], such as binary outcomes common in recidivism. Perhaps the most popular implementation of GLMs is logistic regression: It transforms the probability of an event occurring using the logit function (log-odds), and then it fits a linear relationship between the transformed probability and the predictor variables via maximum likelihood [31]. Logistic regression has been used to create forecasting instruments since the 1940s partially due to its transparency and acceptable predictive validity [13], and for this reason, it is used throughout the recidivism forecasting literature [32, 34, 40]. However, other studies have added regularization to its fitting algorithm through techniques like ridge and lasso, boosting the predictive validity of this instrument [30, 35]. When implementing this algorithm, then, we will use both a standard algorithm and one that implements these regularization techniques.
- *A neural network* is a machine learning model inspired by the structure of the human brain, designed to approximate complex functions [13]. It consists of layers of interconnected nodes (neurons), where the input layer processes raw data, hidden layers extract patterns, and the output layer generates predictions. Neural networks are powerful for recidivism datasets because they use nonlinear transformations to model intricate relationships where, as a general rule, the more hidden layers a network has, the more nonlinear it is. However, they are limited in that they are extremely sensitive to tuning parameters and are prone to overfitting, making them difficult to implement [31].

### Fairness

Criminal justice data often contain human biases [20], which machine learning models can inherit. To mitigate this, we used conviction data instead of arrest data. This decision means that, to be labeled as recidivating, each offender must pass a very high evidentiary bar—they must have so much evidence stacked against them that it culminates in a judicial determination of guilt, that is, a conviction. This high evidentiary bar means that those who were labeled as recidivating actually did, suggesting that our machine learning algorithms will predict recidivism using patterns of people who we are virtually certain committed another crime. Of course, this high evidentiary bar also means that undetected crimes and crimes that do not culminate in a conviction will go undetected. However, including these lower-threshold crimes would risk the introduction of serious bias into the dataset such that people who are wrongfully arrested or stopped due to police suspicion could be labeled as recidivating, which would then be recreated by a machine learning model. Thus, by training our models on conviction data over arrest, we ensure that those labeled as recidivating actually did, thereby addressing a serious form of bias present in police data.

#### The issue of fairness metrics: technical and semantic ambivalence

However, conviction data can still contain human biases that are reflected in the judicial system of England and Wales, such as racial disparities in imprisonment [41]. To address these biases, we reviewed the literature on machine learning fairness as it relates to binary classification tasks typical in recidivism [19]. In other words, to remove human bias from our models, we need to define what lack of bias—or what *fairness*—looks like. Thus, the expressions “debias” and “implement fairness” will be treated as synonymous.

Therein lies a problem: There is no uncontested definition of fairness. Indeed, many fairness definitions exist [19], yet they contradict each other technically [22, 42, 43] and semantically [44, 45]. From a semantic standpoint, there is no publicly accepted, uncontested definition of fairness. Specifically, there are often large base-rate recidivism differences between protected groups: One group of people is statistically more likely to recidivate than another, and the reason for these differences is the subject of ongoing scholarly debate (e.g., [28] versus [46]).

If these base-rate differences reflect societal bias, then a form of “affirmative action” may be needed, where the model’s performance on one group may need to be disadvantaged to equalize base rates between protected groups. This affirmative-action-like procedure mirrors the implementation of the statistical parity fairness metric [42], and it may generate controversy. Conversely, if these base-rate differences do not reflect bias, then a different fairness definition like calibration can be implemented in which these base-rate differences are instilled into the model. Implementing calibration, however, may attract criticism such that we are recreating historical biases in the model. Thus, any fairness metric that we implement may generate controversy because of the lack of semantic agreement on what constitutes fairness.

From a technical standpoint, the large base-rate differences common in criminal justice data will produce different types of errors, and it is mathematically impossible for all types of errors to be simultaneously standardized between protected groups [42]. An illustration of this mathematical incompatibility appears in the trade-off between predictive parity and equal opportunity [22] in that practitioners will need to navigate a difficult scenario: Assuming large base-rate differences, is it more important that all groups suffer the same proportion of false negatives—that each protected group has roughly an equal proportion of high-risk offenders who are incorrectly labeled as low-risk and thereby evade detection—or is it more important that all groups suffer an equal proportion of false positives—of low-risk offenders who are incorrectly labeled as high risk and thereby incorrectly receive a police intervention? Implementing the former may come at the price of exacerbating the latter and vice versa.

In short, fairness metrics and their implementation can be used to mitigate human bias within a model. However, it is technically and semantically difficult to implement all these definitions simultaneously. Thus, practitioners need to navigate difficult semantic and technical decisions to decide which fairness metric is most appropriate.

#### Fairness scale: assumptions and overview

To make these difficult semantic and technical trade-offs palatable to practitioners, we propose a fairness scale. Specifically, the fairness scale was designed so practitioners can quickly identify different definitions of fairness and their assumptions, thereby allowing them to understand how a model is debiased. Moreover, the fairness scale corrects a problem in the literature in that most recidivism forecasting studies do not examine fairness; and of the two studies that do [32, 40], they do not majorly discussing the semantic or technical ramifications of choosing that metric over others. Other studies provide a comprehensive overview of major fairness metrics [19, 42], but presenting a large number of fairness metrics and definitions to criminal justice stakeholders may not be effective—especially when some of these metrics may be irrelevant, and all proposed metrics necessitate thorough explanation to navigate the difficult technical and semantic decisions they imply.

Therefore, the purpose of this fairness scale is to identify major fairness definitions most relevant to criminal justice practitioners, as well as to show which fairness definitions can be easily implemented simultaneously. In doing so, we render the complex literature on fairness metrics palatable to criminal justice practitioners, and we answer an existing call in the fairness literature to define a subset of fairness metrics relevant to each domain [19]. To create the fairness scale, we simplified the fairness literature by using the following assumptions.

First, forecasting models either output a binary yes/no classification, or it can output a risk score, and these outputs require different fairness metrics [19]. Most machine learning models used in recidivism forecasting output a binary yes/no classification. Thus, we restrict fairness metrics to only those compatible with a binary output. This excludes risk-score-based metrics like calibration and positive/negative class balance.

Second, there are typically large base-rate differences in recidivism data, making the simultaneous implementation of many fairness metrics incompatible [22, 42, 43]. Thus, the fairness scale assumes these large base-rate differences will exist in the underlying datasets that implement this scale. This does not change the fairness metrics that we select; however, it does change how we design the scale such that the technical trade-offs between these metrics become salient.

Third, criminal justice interventions informed by forecasting are typically asymmetrical in that one type of prediction matters far more than the other. In other words, one prediction tends to lead to high-impact action, whereas the other type of prediction often leads to little happening. For example, we envision that our model will be used by local police to select convicted offenders to monitor after they are released from prison. If our model outputs a negative prediction, i.e., does not predict recidivism, then nothing happens. However, if the model outputs a positive prediction, then high-impact interventions can ensue in attempting to deter a future crime from happening. This is just one example use case; however, similar logic holds for models used in parole hearings, bail decisions, and for those used in forecasting future police misconduct, among others.

Ideally, both positive and negative predictions would be equally fair. However, the implementation of different fairness metrics often requires steep technical trade-offs [42] such that practitioners may need to prioritize implementing fairness on one type of prediction over the other. In this regard, we selected fairness metrics whose implementation prioritizes the mitigation of human bias within the high-impact prediction, which, in this case, refers to positive predictions. For use cases in which negative prediction is more impactful, these fairness metrics can easily be reconfigured to focus on those predictions instead—for example, using negative prediction value over precision when implementing predictive parity.

Fourth, fairness metric implementation can be political. Thus, we restrict our search to fairness metrics that implement a definition that semantically corresponds to fairness as defined in the popular imagination. In other words, we will likely need to present our models to external stakeholders, who, in turn, may need to explain them to those they represent. In this regard, it would be preferable to use fairness metrics whose definitions correspond to fairness as is popularly understood or brought up in vernacular. This assumption renders semantically simple and popular fairness definitions like fairness through unawareness desirable whereas more complicated ones are less so. This assumption also prioritizes fairness metrics that focus on errors and group-level differences because both—with their corresponding impact on human lives—tend to be well-understood.

**Fig. 2:**
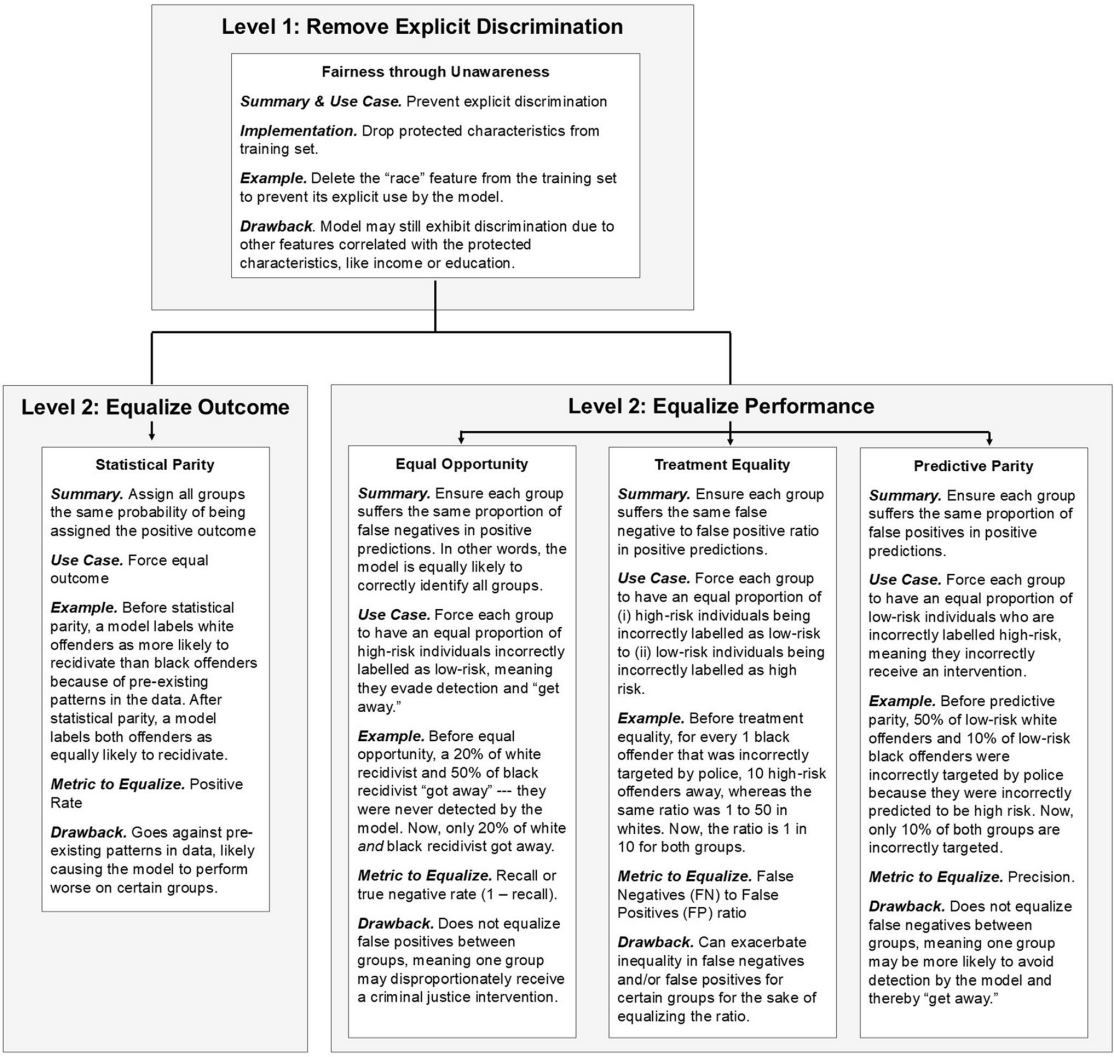
Proposed fairness scale. Gray boxes indicate intention, whereas white box contains a fairness metric and detailed description. The scale begins with one level 1 definition, fairness through unawareness, which argues that a model is fair if it does not explicitly use a protected attribute. After implementing fairness through unawareness, the scale suggests an additional level 2 fairness definition can be implemented. Due to semantic and technical contradictions between these metrics, only one level 2 definition may be implementable in criminal justice datasets

Fifth, we designed the fairness scale so that it implements sparsity—so that it only depicts a few popularly understood definitions of fairness that are highly relevant to criminal justice applications. In other words, we limited our scale to only contain five fairness metrics because sparse models tend to be well-understood by humans according to the interpretable machine learning literature, as they represent the limits of working memory [47]. While it is possible to include additional fairness definitions that are less relevant, this added complexity would obfuscate the distillation of this scale to those stakeholders who do not have a mathematical or machine learning background. With these assumptions enumerated, the fairness scale appears in Fig. 2.

To navigate the technical and semantic ambiguities inherent in implementing a fairness metric, the scale is split into two levels. Level 1 contains one fairness definition—fairness through unawareness—which represents the first step criminal justice practitioners can take to remove human bias from their model. To implement fairness through unawareness, a model should not explicitly use protected characteristics within its decision-making process [19]. This can be easily implemented by removing protected characteristics from the training set. Moreover, it does not majorly contradict any other fairness metrics; it is powerful in that it protects criminal justice agencies from the reputational damage associated with using models that explicitly use protected characteristics; and it corresponds nicely to a basic, widely-understood definition of fairness. Due to its simplicity, its compatibility with other fairness metrics, and its ease of implementation and understanding, it is intended to be the first fairness definition that criminal justice practitioners implement.

Fairness through unawareness will not fully resolve the issue of protected characteristics due to them appearing in more implicit forms. To further implement fairness, practitioners can implement a level 2 definition. These definitions can contradict each other, both technically and semantically, especially in criminal justice datasets with significant base-rate differences [42, 43]. As such, we limit our approach to only implementing one level 2 definition at a time. The first level 2 definition is statistical parity. Statistical parity equalized outcome: It ensures an equal proportion of each protected group is labeled as high risk *regardless* of base-rate differences. In contrast, the remaining level 2 definitions attempt to equalize model performance, not outcome. These definitions are (i) equal opportunity, in which an equal proportion of each protected group suffers the same *false negatives* in positive predictions, (ii) predictive parity, in which an equal proportion of each protected group suffers the same proportion of *false positive* in positive predictions, and (iii) treatment equality, in which each group suffers the same proportion of errors, as measured by a false negative to false positive ratio. An overview of each appears below.

#### Fairness scale: detailed overview

We use the following notations to define our level 2 fairness definitions, using race as the protected attribute. By protected attribute, we mean that the presence of race within our dataset may contain human bias; therefore, implementing these fairness metrics can address that bias. We do not formally define our level 1 fairness definition—fairness through unawareness—because of its simplicity.

- *E*: Protected Attribute. The characteristic that should not unfairly influence predictions, which, in this investigation, is race (ethnicity). Four races will be considered: Asian (*a*), Black (*b*), Other (*o*), and White (*w*).
- *X*: Additional Attributes. All other relevant individual characteristics used for prediction, such as criminal history.
- *Y*: Actual Classification Result. This indicates whether the offender actually did recidivate.
- *S*: Predicted Probability. Probability assigned by the model that an individual belongs to a certain classification, *c*, computed as *P*(*Y* = *c* ∣ *R*, *X*).
- *d*: Predicted Decision. A binary classification derived from S based on a threshold. For example, if S is above 0.5, *d* predicts recidivism model predicts recidivism.

*Statistical parity* is achieved when all groups have an equal probability of being assigned to the positive predicted class, regardless of their actual probability. Formally, a model must satisfy *P*(*d* = 1|*E* = *e*_*i*_) = *P*(*d* = 1|*E* = *e*_*j*_), ∀ *e*_*i*_, *e*_*j*_ ∈ {*w,b,a,o*}, which, in the context of race, would mean all races of convicted offenders would be predicted to recidivate in equal proportions. Statistical parity does not account for differences in base rates of recidivism between groups. Thus, its implementation can force the model to ignore statistical differences in recidivism rates, leading to a decrease in performance for certain races.

This definition is important because racial disparities in recidivism rates may reflect systemic biases rather than inherent differences in criminal behavior. Thus, implementing statistical parity could help counteract these disparities by ensuring equal outcome across groups. However, it is controversial because it does not distinguish between disparities caused by bias and those reflecting real differences in criminal behavior. Insofar as much as base-rate differences in recidivism are due to legitimate factors, forcing statistical parity could lead to unfair outcomes like overestimating risk for one group, potentially leading to unjust outcomes.

*Equal opportunity* ensures that individuals who truly belong to the positive outcome group—those who will recidivate—have an equal chance of being correctly classified, regardless of protected attribute. Formally, this means the model must satisfy *P*(*d* = 1|*Y* = 1, *E* = *e*_*i*_) = *P*(*d* = 1|*Y* = 1, *E* = *e*_*j*_), ∀ *e*_*i*_, *e*_*j*_ ∈ {*w,b,a,o*} ensuring that true positive rates (recall) *and* false negative rates (1-recall) are equal across groups. In the criminal justice context, this means that an equal proportion of each protected group “get away”—that the model fails to detect an equal proportion of high-risk offenders, preventing them from receiving the intervention. Equal opportunity—like all remaining level 2 definitions—equalizes some aspect of performance, not outcome: It ensures the model performs equally well on all protected groups, allowing these large base-rate differences to persist.

A machine learning model will output a list of predictions—names of convicted offenders it predicts will recidivate. *Predictive parity* ensures that the proportion of these names who will actually recidivate is the same between protected groups, or, in other words, that both groups suffer the same proportion of false positives in positive predictions. Formally, this means the model must satisfy *P*(*Y* = 1| *d* = 1, *E* = *e*_*i*_) = *P*(*Y* = 1| *d* = 1, *E* = *e*_*j*_), ∀ *e*_*i*_, *e*_*j*_ ∈ {*w,b,a,o*}. In the context of criminal justice, false positives can be costly in that low-risk individuals are wrongly given high-impact interventions. Predictive parity helps ensure that no group disproportionately suffers from false positives, promoting fairness in how police interventions are applied.

*Treatment equality* equalizes the balance of errors rather than focuses on correcting one specific error. A model satisfies this definition if the ratio of false negatives (FN) to false positives (FP) is equal across protected and unprotected groups. Formally, this means: $\frac{{{\text{FN}}}}{{{\text{FP}}}}e_{i}$ = $\frac{{{\text{FN}}}}{{{\text{FP}}}}e_{j}$, ∀ *e*_*i*_, *e*_*j*_ ∈ {*w,b,a,o*}. In the criminal justice context, treatment equality ensures that each group suffers the same proportion of false negatives and false positives—for example, for every low-risk offender that is incorrectly predicted to be high risk, there are five high-risk offenders who are incorrectly predicted to be low-risk, and this ratio persists between protected groups. However, this does not necessarily reduce overall disparities—it only ensures that the trade-off between overestimating and underestimating risk is the same for all groups.

### Synthesis

In short, two literatures were reviewed to initialize the parameters by which we construct our recidivism forecasting models. First, we reviewed the literature on recidivism forecasting via a recent literature review [17], extracting the following common parameters by which we will construct our models:

- *Defining forecasting.* We will forecast both general and violent recidivism to mirror a trend in the literature. *General recidivism* is defined as any reconviction in a 3-year period, whereas *violent recidivism* is defined as any reconviction for a violent offense within a 3-year period, with violent reconviction being defined as a hybrid between [30, 31] narrow and expansive definitions of violence, respectively.
- *Dataset selection.* Consistent with most authors, we will use a conviction dataset as the foundation by which our models will be trained to predict recidivism. This allows us to combat societal biases commonly found in arrest data [20]. Moreover, we selected a sparse dataset that is already used in the existing criminal justice system to show how machine learning can be integrated into the existing criminal justice technical infrastructure.
- *Algorithm selection.* We will construct a general and violent recidivism forecasting model via any algorithm that outputs a best-performing model based on the reviewed studies. This yielded a selection of 5 algorithms: a random forest, two variations of a generalized linear model, linear discriminant analysis, boosting, and a neural network.
- *Evaluation methods & metrics.* We will use cross-validation as the method by which our models are evaluated because it is what most authors have done, and it gives a better sense of a model’s error rate when compared to the computationally-cheaper train–test split [36]. We will evaluate our model via AUC scores because it is the common metric by which we can compare our model to other state-of-the-art instruments, in addition to other metrics relevant to understanding the performance of our model as it is implemented in practice (see methods).

Second, we reviewed the fairness literature so that we can mitigate human bias within our models. Specifically, we proposed a fairness scale that simplified the semantic and technical contradictions of the fairness literature in a way that criminal justice practitioners can understand. That scale yielded one level 1 definition of fairness, fairness through unawareness, which represents an easy-to-implement, well-agreed-upon definition of fairness. It also presents multiple level 2 definitions that build upon the level 1 definition—definitions whose implementation may require practitioners to make difficult semantic and technical decisions as to what constitutes fairness (see Fig. 2). We will implement each definition of fairness within our models.

## Methods

### Ethics

All methods were performed in accordance with relevant guidelines, regulations, and protocols. The Ethics Committee of the Institute of Criminology, University of Cambridge formally approved of this experiment, including all protocols, guidelines, and regulations followed, as well as provided ethical oversight. This ethics approval was obtained via the standard application process: by submitting the full ethics application to the Institute of Criminology, University of Cambridge (B/Ethics/February Version 9), along with a completed risk assessment (RA7.12) and research abstract, before being reviewed and ultimately approved by the committee.

Moreover, this study analyzed legacy crime data that was collected as part of a standard criminal justice process. Due to the retrospective nature of the dataset, the infeasibility of other research designs, and the minimal risk of harm to individuals, the need for informed consent was waived by the Ethics Committee of the Institute of Criminology, University of Cambridge. The dataset was fully anonymized before being handed to the authors.

### Overview

The Police National Computer (PNC) is a national database available to all police forces in the UK, as well as additional non-police organizations [25] that contains conviction and disposition records, among others [1]. On February 3, 2011, PNC records that contained convictions were made available to the research team as part of a wider research program into offender desistance, which includes Operation Turning Point to target low-harm offenders [48]. The research was funded by Monument Trust and had a steering group including a senior judge, a senior prosecutor, a probation chief, and a chief constable. Nevertheless, the resulting dataset produced 1,641,775 PNC records that contained 30 features.

**Fig. 3:**
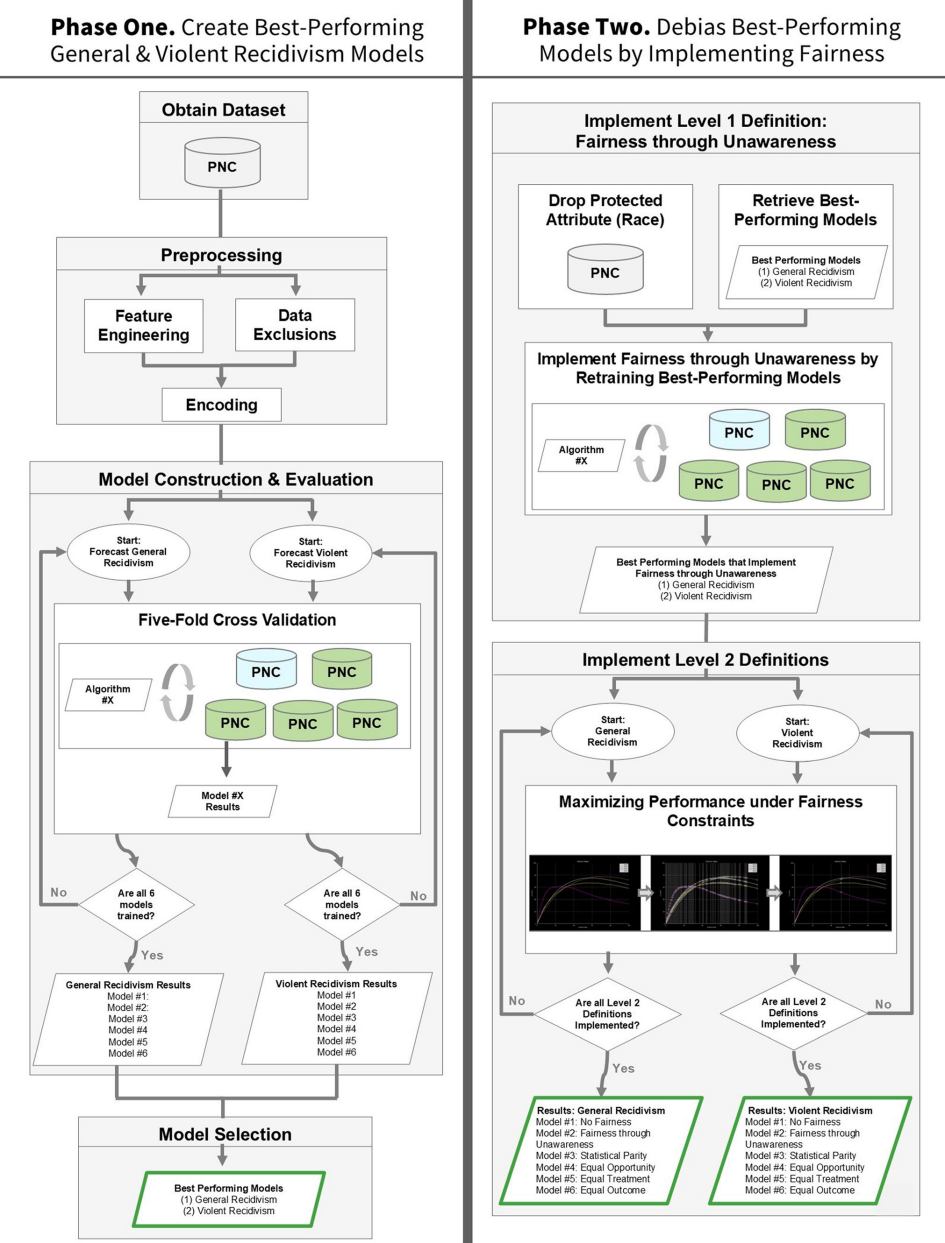
Methods overview. In phase one, we create twelve models—six that can forecast general recidivism, six that can forecast violent recidivism—and select the best model. In phase two, we debias our best-performing model via a fairness scale. Arrows indicate flowing from one step to another. Steps are grouped together by intention, depicted via gray boxes. Cylinders indicate dataset: Blue cylinders indicate a dataset partition that is part of the testing set, whereas green indicate the equivalent for a training set. White squares indicate subprocess. Circles indicate start of loop. Parallelogram indicates output, whereas diamonds indicate decision points. Model optimization (hyperparameter tuning) is omitted from the schematic due to size constraints; yet, it would appear in fivefold cross-validation

After obtaining the PNC records, the investigation was conducted in two phases, as illustrated in Fig. 3. In phase one (Sects. 3.3–3.5), we construct 12 forecasting models—6 that forecast general recidivism, 6 for violent recidivism—and select the best-performing model for each category. Both best-performing models outperform the existing state-of-the-art. In phase two (Sects. 3.6–3.7), we debias our best-performing models by implementing each definition of fairness as it appears in the Fairness Scale. This involves creating a model that implements fairness by unawareness, as well as imposing fairness constraints on that model such that each level 2 fairness definition is implemented. Both phases are illustrated in Fig. 3.

### Preprocessing with dataset overview

Preprocessing was undertaken to transform the PNC into a dataset legible to a machine learning algorithm, as well as to improve the predictive validity of the ultimate model. Specifically, our preprocessing technique occurred in three steps: (i) feature engineering, (ii) data exclusions, and (iii) encoding. Feature engineering was used to construct features that can boost the model’s performance as well as clean the dataset. A summary of our feature engineering operations appears in Table 5 for general features, Table 6 for criminological features, with full justification for each operation appearing in the suppl. methods. In short, feature engineering accomplished the following major intentions:

- Render the dataset legible to machine learning such that a forecasting model could be constructed using that dataset, using techniques proposed by [49]. This involved removing features that may be irrelevant to a prediction, such as the individual offender’s ID; creating features that summarize redundant existing features, such as extracting total prison sentence from the sentence embedded within each disposition; and extracting key information embedded within existing features, among other techniques. See Table 5 for summary, suppl. methods for further justification.
- Construct each offender’s criminal history because recent criminal history has been shown to significantly predict future reoffending [50].
- Construct predictors using criminological theories because injecting relevant domain knowledge into a forecasting model often increases performance [51]. This involved consulting with criminologists to devise features that leveraged criminological theory to detect phenomena like crime specialization, assign a risk level based on the age–crime curve, and leverage proxies for a criminogenic location, among others. See Table 6 for summary, suppl. methods for further review and justification.

**Table 5:** Summary of feature engineering

Techniques used, grouped by intention (number of features affected)	Feature name	Data type (subtype)	Example value(s)	Justification (feature deletion) or description (feature creation)
*Make offender’s offenses legible to machine learning*				
Create feature (1)	Involved minor	Boolean	True, False	Flag whether an offense involved targeting a minor, or an individual under 18. Based on Home Office Offense Code
Create feature (1)	Involved weapon	String	Gun, Knife, Bomb	Highlight whether an offense involved a weapon, and if so, lists the type. Based on Home Office Offense Code
Delete feature (1)	Home office offense code	String	00200, 10110	Offense codes are hyper-specific and semi-arbitrary, with little underlying semantic relationship. Their inclusion may confound a model The feature “Offense Class” already summarizes an offense. Details that demand more granularity were extracted using this feature; they appear above
*Flag whether an offender will recidivate within 3 years*				
Create feature (1)	3-year all recidivism	Boolean	True, False	Flag whether the offender was reconvicted for any offense over the next 3 years
Create feature (1)	3-year violent recidivism	Boolean	True, False	Flag whether the offender was reconvicted for a violent offense over the next 3 years
*Understanding offender’s age*				
Create feature (1)	Age at time of disposition	Integer	50, 15	Age of offender when he/she received his/her disposition. Created from subtracting court date from the offender’s age at time of PNC download
Create feature (1)	Minor at time of disposition	Boolean	True, False	Flag whether the offender was less than 18 years old at time of disposition because that affects sentencing
Delete feature (1)	Age	Integer	53, 18	How old the convicted offender was at the time of PNC download, i.e., February 2011. Age is better measured via “Age at time of disposition,” feature; hence, it was redundant and dropped
*Understand offender’s disposition(s)*				
Delete feature (4)	DispText1, DispText2, DispText3, DispText4	String	“Ticket No. 39521”, “Costs #54.00”	Each convicted offender could receive up to 4 dispositions. This feature records any notes for each disposition Over 80% of each DispText feature is empty; and of the few DispText values that are initialized, the notes are not recorded systematically. It is difficult to extract meaning from non-standardized features that are mostly missing values, hence, they were deleted
Delete feature (4)	DispRank1, DispRank2, DispRank3, DispRank4	Integer (categorical)	1, 2, 3, 4	Gives the order that the dispositions were given in. However, the order of each disposition is already implied in its name. For example, DispCat1 refers to the category of the first disposition, DispCat2 for the second, etc. Hence, these features were redundant and dropped
Create feature (1)	Total custodial sentence	Integer	30, 120, 720	The number of days that the convicted offender was sentenced to prison. Namely, each disposition that the convicted offender received could have sentenced him/her to prison for varying numbers of days. This feature records his/her total sentence. Extracted from summing DispDays 1–4
Delete feature (4)	DispDays1, DispDays2, DispDays3, DispDays4	Integer	7, 7, 21, 60	The number of days in prison that each disposition required the convicted offender to serve. The total custodial sentence—rather than the individual custodial sentence associated with each disposition—is far more meaningful; hence, these were discarded
Create feature (1)	Total fine	Float		The total fine that the convicted offender was required to pay, if any, in Pounds Sterling (£). Extracted from summing DispAmt 1–4
Delete feature (4)	DispAmt1, DispAmt2, DispAmt3, DispAmt4	Float	9.63, 10.00	The fine associated with each individual disposition. Total fine is far more meaningful, rendering these features largely redundant. Hence, they were dropped
*Constructing criminal history*				
Create feature (11)	Total Criminal Damages over Lifetime, Total Sex Offenses over Lifetime	Integer	0, 1, 15	For each of the eleven offense classes, a counter feature was created that counts the number of times an offender was convicted of an offense that fell within the corresponding class
Create feature (1)	Ever involved with weapon	Boolean	True, False	Flag whether an offender has ever been convicted of an offense involving a weapon
Create feature (1)	Ever targeted minor	Boolean	True, False	Flag whether an offender has ever been convicted of an offense involving a minor
*Miscellaneous: remove other confounding features*				
Delete feature (1)	Court Date	DateTime	31/10/2002, 1/1/2003	Date the convicted offender received his/her disposition(s). Removed because machine learning algorithms tend to not know how to handle DateTime values, and extracting additional information from these values (e.g., court month, court year) would not meaningful
Delete feature (2)	Offense Force Code, Process Force Code	Integer (categorical)	42, 12	Numeric ID representing the force in which the offense was committed, or the force in which the offense was processed. This is a proxy for the location of each offender There are 73 unique values for process force code, with offense force code containing a similar number. To meaningfully use these values, we would need to one-hot encode them, adding 73 new columns to our dataset. The addition of location (i) did not majorly affect model performance and (ii) massively slowed down training time in pilot studies. Thus, both location proxies were removed, though it may be explored in a future project
Delete feature (1)	Address Postcode	String	RH11 9JR, DA1 5JL	This feature mostly contained missing values. Additionally, adding location features massively slowed down training time while not majorly affecting model performance (see Offense Force Code, Process Force Code), hence, it was removed
Delete feature (1)	isPrimaryOffense	Boolean	True, False	An offender could be convicted of multiple offenses on a single court date. This Boolean flags whether an offense was the main offense a convicted offender was convicted for that day, or an auxiliary offense. Main offenses were those that were deemed to be the most serious relative to other simultaneous offenses This feature was deleted because our final dataset only contained primary offenses, hence, it became a non-meaningful constant (see data exclusions)
Delete feature (1)	Person ID	Integer	92, 3021	A numeric ID representing each convicted offender. Removed because we did not want a convicted offender’s name to influence the model’s recidivism prediction

Full details appear in the suppl. methods and results

**Table 6:** Summary of criminological feature engineering

Techniques used, grouped by criminological insight (number of features affected)	Feature name	Data type (subtype)	Example value(s)	Justification (feature deletion) or description (feature creation)
*Criminal careers*				
Create feature (1)	Age of first disposition	Int	15, 24, 18	Measure the onset of criminal activity
Create feature (2)	Court appearance count, offense count	Int	1, 2, 12	Measure offending frequency
Create feature (1)	Years since last disposition	Float	1.3510, 0.62	Measure desistence (attempts), that is, how long an offender went without another conviction
Create feature (1)	Diversity index	Float	0, 0.732	Measure extent of crime specialization where 0 indicates complete specialization, and the maximum value 0.91 indicates complete lack of specialization
*Reintegrative shaming*				
Create feature (2)	Ever receive custodial sentence, ever receive fine	Boolean	True, False	Flag whether an offender has ever been sentenced to prison or received a fine, respectively
Create feature (1)	Total custodial sentence over lifetime	Integer	360, 3600	Record the number of days a convicted offender was sentenced to prison over his/her lifetime
Create feature (1)	Total fine over lifetime	Float	452.35, 2050.00	Records the total fine that the convicted offender received over his/her lifetime, in Pounds Sterlin (£)
*Age–crime curve*				
Create Feature (1)	Age–Crime Risk Level	String	High, Medium, Low	Flag whether an offender is high, medium, or low risk based on his/her age relative to the age–crime curve
Criminogenic location				
Create feature (2)	Total offenses per 1000 people, total drug offenses per 1000 people	Float	2.41, 0.81	Loose proxy for how criminogenic a location is, aggregated at the level of the jurisdiction that a UK law enforcement agency protects
*Other risk factors*				
Create feature (1)	Substance Abuse Risk	Boolean	True, False	Measure whether an individual has ever been convicted of drug possession

Criminological features were constructed after all other feature engineering steps were undertaken. Full details appear in the suppl. methods and results

Data exclusions were undertaken to overcome issues such as simultaneous convictions, concept drift, and an insufficient measurement window, among others. A summary of data exclusions appears in Table 7, with full details appearing in the suppl. methods. In short, these data exclusions resulted in a final dataset of 346,685 rows, where each row represented a convicted offender at a particular court date between January 1, 2000, and February 3, 2006.

**Table 7:** Exclusion summary

Issue with description	Proposed solution	Number of exclusions
*Simultaneous convictions* An offender may be convicted of multiple offenses during one court date, resulting in multiple predictions per case	Drop non-primary offenses such that the most serious offense (primary) is the one used as the foundation for the prediction The dropped (non-primary) offenses are not omitted from the model’s decision-making process: They are captured in the criminal history feature and are thereby captured in predictions	774,446
*Concept drift* Early convictions, like those in the mid-to-late twentieth century, may not accurately reflect modern cases. This could cause our model to learn outdated patterns in data, thereby impairing performance	Drop early convictions, or those before January 1, 2000	365,877
*Insufficient measurement window* It does not make sense to measure recidivism for recently released offenders because they do not have enough time to recidivate	Drop recent convictions, or those that occurred after February 3, 2006	154,658
*Individuals with an anomalous age* Some individuals were assigned an infeasibly old age. Because age is one of the greatest predictors of recidivism, including these individuals could confound a model’s ability to learn patterns in a highly predictive feature	Drop individuals who were 111 years old, as there is an unusual amount of 111-year-olds (*n* = 109) and there is a ten-year age difference between the 111-year-olds and the next oldest offender, aged 99 (*n* = 1)	109
Total exclusions		1,295,090

Exclusions are grouped by the overarching issue that the exclusion solved, with further details appearing in the suppl. methods and results

Finally, one-hot encoding was undertaken to transform all categorical features (strings) into binaries to boost the performance of the ultimate model. Descriptive statistics portraying the final dataset appear in suppl. Tables S1 and S2, with an illustration of the contents of the dataset appearing in suppl. Table S3.

### Model construction and optimization

All models were trained, optimized, and evaluated using Python 3.10.12 and GCC 11.4.0 via JupyterLab 4.0.5, using popular libraries like Matplotlib 3.5.1, Pandas 2.1.0, and NumPy 1.24.3. Specifically, all models were trained on a machine that used a 64-bit Windows operating system running the Windows Subsystem for Linux (Ubuntu 22.04.3). The machine contained 16 GB DDR5 RAM, a relatively powerful AMD Ryzen 9 7940HS CPU, and NVIDIA GeForce RTX 4060 GPU, which greatly facilitated the training and optimization of these models.

We selected 5 algorithms or algorithm families that have output a best-performing forecasting model based on the reviewed studies, as discussed in Sect. 2.1.4 and Table 4. From these 5 algorithms or families, we construct 6 different models per label. Specifically, we construct one model per algorithm, with the sole exception being the generalized linear models—we construct two models from this family due to substantial differences in their best-performing implementation [30, 35, 40]. Thus, there are 12 models total: 6 models that forecast general recidivism and 6 that forecast violent recidivism.

All models were trained and optimized using nested *k*-fold cross-validation, in which *k* was set to five for both the inner and outer loops. Hyperparameter tuning was used to optimize models; hence, nested cross-validation was used over standard cross-validation to prevent potential data leakage. The purpose of hyperparameter tuning was to empirically identify the best hyperparameter combinations by which our models could be constructed, based on the highest AUC score (see Sect. 2.1). Hyperparameter tuning was undertaken via a random grid search, in which 100 different combinations were tested. The sole exception to this procedure was a neural network. We used manual hyperparameter tuning due to the notorious difficulty of optimizing a neural network; thus, standard fivefold cross-validation was used over nested. Details behind the implementation of each algorithm appear below.

- *Random forest.* Breiman’s algorithm [52] served as our implementation of the random forest because this is a standard algorithm by which random forests are constructed.
- *Boosting: gradient boosting machine.* Friedman’s algorithm [53] served as our implementation of a boosting algorithm because this is a standard algorithm by which gradient boosting machines are constructed.
- *Implementing linear discriminant analysis.* Our implementation of linear discriminant analysis draws from [39] using single-value decomposition, which is consistent with the reviewed work that leveraged this approach (see Table 4 and Sect. 2.1.4).
- *Implementing a neural network *via* multilayered perceptron.* A multilayer perceptron architecture was used to implement a neural network [39], which is consistent with past work using a neural network (see Table 4 and Sect. 2.1.4). This approach leveraged Adam optimization, a rectified linear unit activation function, and a hidden layer configuration of (256, 128, 64, 32) neurons. The implementation details, such as the hidden layer configuration, were chosen via manual hyperparameter tuning.
- *Implementing generalized linear models *via* (i) logistic and (ii) Elasticnet regression.* There were three studies in which the GLM was the best-performing model. One study used a standard logistic regression [40], whereas the other two studies used some variation of an Elasticnet logistic regression (“Elasticnet”), which is a logistic regression that uses ridge and lasso regularization [30, 35]. Thus, both implementations were leveraged.

### Evaluation metrics: performance

We use two sets of evaluation metrics to evaluate our model: One set measures performance, whereas the other set measures fairness. To measure performance, five evaluation metrics were used: recall, precision, specificity, the area under the receiver operator characteristics curve (AUC), and *F*_1_ score. They were selected because they are apt performance proxies for the criminal justice space and are used somewhat ubiquitous in the forecasting literature as it relates to criminology and other fields [17, 37, 54]. To measure fairness, four metrics were used, one metric for each level 2 definition of the fairness scale: the statistical range (*R*) of the positive rate (*R*_PR_) to measure statistical parity, the statistical range of recall (*R*_Recall_) to measure equal opportunity, the statistical range of precision (*R*_Precision_) to measure predictive parity, and the statistical range of the false negative to false positive ratio (*R*_Error_balance_) to measure treatment equality. A summary of these evaluation metrics appears in Table 8.

**Table 8:** Summary of major evaluation metrics

Metric	Formula	Description
**Performance**		
***Granular metrics***		
Recall	$\frac{{{\text{TP}}}}{{{\text{TP}} + {\text{FN}}}}$	Proportion of high-risk individuals our model can identify
Precision	$\frac{{{\text{TP}}}}{{{\text{TP}} + {\text{FP}}}}$	Model will output a list of names of individuals it predicts will recidivate (“positives”). Precision is the proportion of those names that are correct—i.e., the model labels them as high risk, and they actually recidivate
Specificity	$\frac{{{\text{TN}}}}{{{\text{TN}} + {\text{FP}}}}$	Proportion of low-risk individuals our model can identify
***Overall performance***		
AUC score	$\mathop \sum \limits_{i = 1}^{n} \frac{{\left( {{\text{FPR}}_{i} - {\text{FPR}}_{i - 1} } \right)*\left( {{\text{TPR}}_{i} - {\text{TPR}}_{i - 1} } \right)}}{2}$	How well a model performs across decision all thresholds
*F*_1_ score	$\frac{{2*{\text{Precision}}*{\text{Recall}}}}{{{\text{Precision}} + {\text{Recall}}}}$	How well a model performs at a specific decision threshold
**Fairness**		
***Equal outcome***		
R_PR_	$R_{{{\text{PR}}}} = \mathop {\max }\limits_{e \in E} \left( {{\text{PR}}_{e} } \right) - \mathop {\min }\limits_{e \in E} \left( {{\text{PR}}_{e} } \right)$	Statistical Parity. To which extent the model equalizes outcome, that is, flags the same proportion of each protected group as high risk. Satisfied if *R*_PR_ < 0.01
***Equal treatment***		
*R*_Recall_	$R_{{{\text{Recall}}}} = \mathop {\max }\limits_{e \in E} \left( {{\text{Recall}}_{e} } \right) - \mathop {\min }\limits_{e \in E} \left( {{\text{Recall}}_{e} } \right)$	Equal Opportunity. To which extent the false negative rate is equalized between protected groups as it relates to positive predictions—that is, whether an equal proportion of each protected group “got away.” Satisfied if *R*_Recall_ < 0.01
R_Precision_	$R_{{{\text{Precision}}}} = \mathop {\max }\limits_{{e{ } \in { }E}} \left( {{\text{Precision}}_{e} } \right) - \mathop {\min }\limits_{{e{ } \in { }E}} \left( {{\text{Precision}}_{e} } \right)$	Predictive Parity. To which extent the false positive rate is equalized between protected groups as it relates to positive predictions—that is, whether an equal proportion of low-risk offenders will be incorrectly labeled as high risk and thereby erroneously receive law enforcement resources. Satisfied if *R*_Precision_ < 0.01
R_Error_balance_	$R_{{{\text{Error}}\_{\text{balance}}}} = \mathop {\max }\limits_{{e{ } \in { }E}} \left( {{\text{Error}}\_{\text{balance}}_{e} } \right) - \mathop {\min }\limits_{{e{ } \in { }E}} \left( {{\text{Error}}\_{\text{balance}}_{e} } \right)$	Treatment Equality. To which extent each protected group suffers the same false negative (FN) to false positive (FP) ratio. Satisfied if *R*_Error_Balance_ < 0.01
**Other equations**		
Error_balance	$\frac{{{\text{FN}}}}{{{\text{FP}}}}$	Ratio of false negatives (FN) to false positives (FP). Used to assess treatment equality
PR	$\frac{{{\text{TP}} + {\text{FP}}}}{{{\text{TP}} + {\text{FP}} + {\text{TN}} + {\text{FN}}}}$	Positive Rate. Proportion of all offenders that the model flags as high risk

For fairness metrics, let *E* represent the set of all racial groups considered, defined as *E* = {*a*, *b*, *o*, *w*}, where each element *e* ∈ *E* denotes a specific racial group, *a* denotes Asian, *b* denotes Black, *o* denotes other, *w* denotes white. Bold text indicates category, *bold and italics* indicates subcategory, and plain text indicates an evaluation metric

All evaluation metrics rely on values derived from a confusion matrix, whose summary appears below.

#### Confusion matrix

A confusion matrix represents a prevalent means of illustrating the performance of a machine learning model; it is shown in Table 9. It is a two-by-two contingency table in which the model’s predicted value is the *y*-axis, and the true value is the *x*-axis. Their tabulation produces four measurements that illustrate a model’s performance: true positive count (TP) and true negative count (TN)—the number of recidivism predictions the model got right—and false positive count (FP) and false negative count (FN)—the number of recidivism predictions the model got wrong. Percentages can also be displayed alongside counts. These four measurements are the foundation for all remaining evaluation metrics.

**Table 9:** Example confusion matrix

Actual	Predicted	
	Recidivism	No recidivism
Recidivism	**True positive count (TP)**	*False negative count (FP)*
No recidivism	*False positive count (FP)*	**True negative count (TN)**

Bold indicates correct predictions, whereas italic indicates incorrect predictions

#### Recall

Recall is the proportion of recidivating offenders that the model could identify. For example, if there were 100 recidivating offenders and the model had a recall score of 0.5, then the model could identify 50 of the 100. High recall is crucial to our model because it translates to fewer missed cases. In other words, our model can be used by law enforcement to flag high-risk offenders that will recidivate. In this context, the more high-risk offenders our model correctly detects, the more opportunities law enforcement will have to prevent a future crime. Thus, high recall can translate to more crimes correctly detected and thereby prevented, and its equation appears below [54]:

1 $${\text{Recall}} = {\text{Sensitivity}} = {\text{True}}\;{\text{positive}}\;{\text{rate}}\;\left( {{\text{TPR}}} \right) = \frac{{{\text{TP}}}}{{{\text{TP}} + {\text{FN}}}}.$$

#### Precision

Measuring false positives is critical because they represent low-risk individuals incorrectly flagged as high risk, leading to unnecessary police interventions. Such misallocations can have serious negative impacts on the lives of these individuals, including undue scrutiny or legal consequences. In this context, precision evaluates how effectively the model minimizes false positives. Specifically, the model will output names of individuals predicted to recidivate. Precision is the proportion of those names that will actually go on to recidivate. For example, if our model has a precision score of .75, then this means for every 4 names it outputs, 3 will recidivate whereas 1 is a false positive. Thus, a higher precision score indicates fewer false positives, meaning fewer low-risk individuals are wrongly targeted. Its equation is given by [54]:

2 $${\text{Precision}} = {\text{Positive}}\;{\text{prediction}}\;{\text{value}}\;\left( {{\text{PPV}}} \right) = \frac{{{\text{TP}}}}{{{\text{TP}} + {\text{FP}}}}.$$

#### Specificity

Specificity is the proportion of individuals who do not recidivate that the model successfully detects. It can be thought of as the recall score for negative predictions, and it is crucial for calculating AUC, the evaluation metric that unifies most recidivism studies (see Table 3). It is the mathematical compliment of the false positive rate (FPR), and its equation appears below [54]:

3 $${\text{Specificity}} = {\text{True}}\;{\text{negative}}\;{\text{rate}}\;\left( {{\text{TNR}}} \right) = 1 - {\text{False}}\;{\text{positive}}\;{\text{rate}}\;\left( {{\text{FPR}}} \right) = \frac{{{\text{TN}}}}{{{\text{TN}} + {\text{FP}}}}.$$

#### Area under curve (AUC)

The AUC score is typically used to summarize overall model performance because it provides an indication of the model’s performance across decision thresholds [37]. In other words, each model faces a fundamental trade-off between true positives and false positives. For example, a model may be able to detect more recidivism (true positives) if it lowers the decision threshold needed to flag recidivism; a model can be configured to require 1% confidence to flag recidivism rather than 50% confidence. This decision would undoubtedly result in more recidivism detections, but it would also result in more false positives—hence the trade-off emerges. This trade-off occurs again if one were to raise the decision threshold—requiring 99% confidence to detect recidivism, for example—albeit the consequences are reversed.

This trade-off can be graphically illustrated through the receiver operator characteristics curve (ROC), which plots true positives against false positives across various confidence thresholds. One can take the area under the curve (AUC) of the ROC graph to summarize the overall quality of this trade-off, where higher values indicate a better trade-off. AUC is often used as a measure of overall performance [17, 37, 54], and it is calculated as follows, using the true positive rate and false positive rates that are defined in (1) and (3) [54]:

4 $${\text{AUC}} = \mathop \sum \limits_{i = 1}^{n} \frac{{\left( {{\text{FPR}}_{i} - {\text{FPR}}_{i - 1} } \right)*\left( {{\text{TPR}}_{i} - {\text{TPR}}_{i - 1} } \right)}}{2}.$$

Note that FPR and TPR are vectors of false and true positive rates, respectively, across various decision thresholds, with *n* being their length. AbiNader et al. [37] provide guidance on meaningful interpretation of the AUC score: A score of 1 reflects being a perfect classification, 0.5 reflects a chance classification, 0 reflects a perfect misclassification, and a value of 0.71 or greater reflects an excellent model. Moreover, the AUC score reflects a common benchmark that virtually every recidivism forecasting study reports (see Table 3); thus, its utilization will allow our models to be compared to the existing state-of-the-art.

#### ***F***_1_ score

While the AUC score assesses a model’s overall performance *across* decision thresholds, the *F*_1_ score can summarize a model’s overall performance *at that specific threshold*. Namely, the *F*_1_ score is the harmonic mean between precision and recall. Both precision and recall are critical for assessing our model’s use in practice; thus, its summary statistic—the *F*_1_ score—should provide a sufficient proxy for overall performance. Its equation is provided by [54]:

5 $$F_{1} = \frac{{2*{\text{Precision}}*{\text{Recall}}}}{{{\text{Precision}} + {\text{Recall}}}}.$$

The *F*_1_ score was also chosen because of its ease of customizability. By default, the *F*_1_ weights recall and precision equally. However, it is a special case of the *F*_*β*_ score in which *β* could be altered to implement a custom cost ratio. This custom cost ratio is especially useful for criminal justice practitioners because they often assign a very different weight to false positives and false negatives, allowing these weights to be factored into a model’s overall assessment. For example, in the case of homicide forecasting, one criminal justice agency argued that the cost of 1 false negative—of a future murderer being paroled—is equivalent to the cost of 10 false positives—of low-risk individuals being incorrectly denied parole and thereby kept in prison [55]. This 1-to-10 false negative to false positive ratio can be baked into the overall model assessment via the *F*_*β*_ score, in which *β* would assign far more weight to recall than precision. A custom cost ratio was not implemented in this investigation (*β* = 1); however, the ability to easily do so makes the use of the *F*_*β*_ score more persuasive.

### Evaluation metrics: fairness

**Fig. 4:**
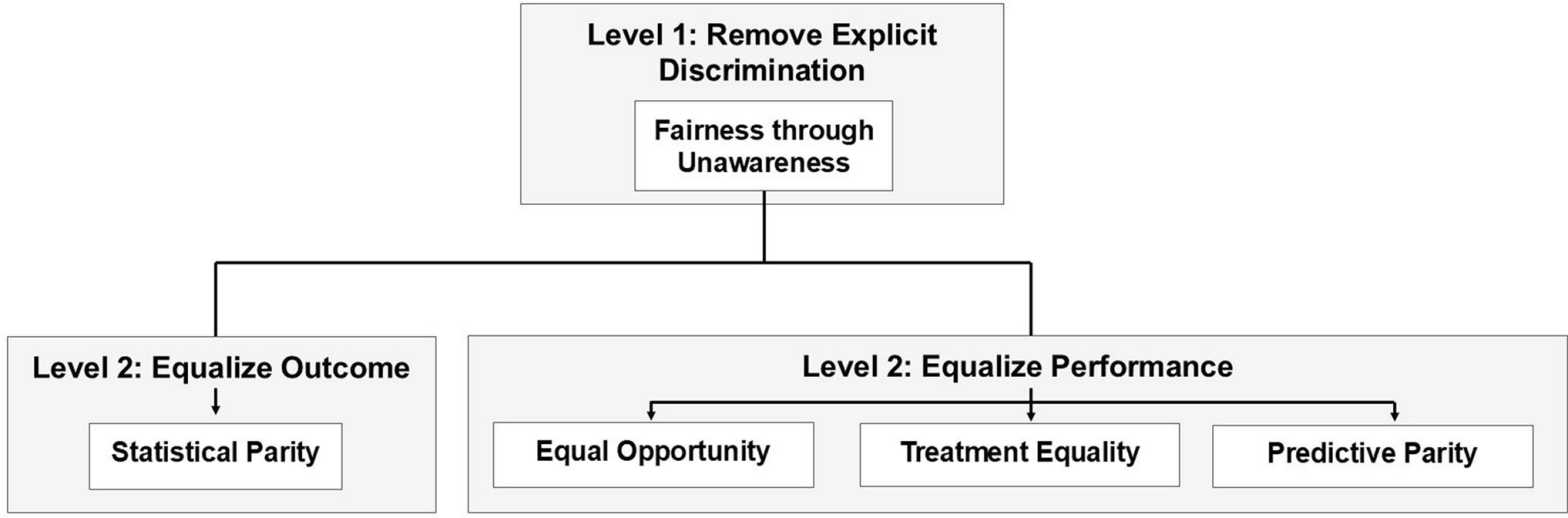
Proposed fairness scale, abridged. Gray boxes indicate intention, white boxes indicate fairness metric

In addition to evaluating performance, we extracted metrics that measure a model’s fairness based on the fairness scale, using race as the protected attribute. An abridged version of the fairness scale appears in Fig. 4. The fairness scale is broken into two levels. The level 1 definition of fairness—fairness through unawareness—needs no specific evaluation metric because it is satisfied simply when the protected attribute—which, in this investigation, is race—is dropped from the training set. Each level 2 definition has its own metric that measures the extent of its implementation, which is introduced below. For reference, an abridged version of the fairness scale appears in Fig. 4.

#### Measurement logic and satisfying a fairness definition

Fairness definition implementation is commonly measured through the statistical range (*R*) of a performance metric, where, the lower its range across protected groups, the greater the extent of the fairness metric’s implementation [56]. The exact performance metric used varies by definition [19], as introduced in Sect. 2.2.3; yet, all definitions are united in that statistical range of their performance metric must be below a tolerance value ϵ to be considered satisfied.

In other words, to satisfy a fairness definition, the statistical range of the performance metric across protected group should ideally be zero because zero indicates that there are no disparities [56]; any value above zero indicates disparity. However, it is extremely difficult to achieve zero disparity using real-world datasets [56], and this is especially true for those arising from the criminal justice system where large base-rate differences in recidivism may be especially conducive to disparity [42]. Thus, a tolerance value ϵ is used where, if the disparity arising from the statistical range is lower than the tolerance, the definition is satisfied [56].

In keeping with this approach, we measure the implementation of a fairness definition by taking the statistical range of the performance metrics associated with each definition across protected groups. The specific performance metric associated with each definition was introduced in Sect. 2.2.3, albeit in a mathematical notation rather than one more common in the machine learning literature. Thus, for the remaining subsections (Sects. 3.6.2–3.6.5), we draw from the mathematical notations introduced in Sect. 2.2.3 to translate them into equivalent equations within the machine learning literature.

To further keep with this approach, if the statistical range of a performance metric falls below ϵ, we consider that metric’s definition satisfied. For this investigation, we chose a tolerance value of 0.01 because it indicates that there is a less-than-one percentage point difference in a performance metric between protected groups. For example, this means that the precision score of all racial groups must be within one percentage point of each other for our model to satisfy predictive parity. This threshold was initialized empirically through implementation (see Sect. 3.7): It was sufficiently small such that it minimized differences between groups, yet it was not so small that it forced the model to use a highly limited set of decision boundaries that impaired model performance. We acknowledge that this ϵ value is somewhat arbitrary; yet, there is no universal value by which ϵ should be set because it is highly context dependent [56]. Moreover, other authors have used a far greater disparity tolerance of 20-percentage points in keeping with the disparate impact heuristic [32, 56]. We viewed this larger tolerance as too great; thus, we elected to use a more rigorous equivalent.

For the remaining subsections (Sects. 3.6.2–3.6.5), let *E* represent the set of all racial groups considered, defined as *E* = {*a*, *b*, *o*, *w*}, where each element *e* ∈ *E* denotes a specific racial group, *a* denotes Asian, *b* denotes Black, *o* denotes other, *w* denotes White.

#### Statistical parity: measured via positive rate range (*RPR*)

Statistical parity equalizes outcome. As introduced in Sect. 2.2.3, it is achieved when the model predicts that an equal proportion of each race will recidivate [19], as measured through the positive rate:

6 $${\text{PR}} = \frac{{{\text{TP}} + {\text{FP}}}}{{{\text{TP}} + {\text{FP}} + {\text{TN}} + {\text{FN}}}}.$$

We use the statistical range (*R*) of the positive rate between different racial groups to measure the implementation of statistical parity [56]:

7 $$R_{{{\text{PR}}}} = \mathop {\max }\limits_{{e{ } \in { }E}} \left( {{\text{PR}}_{e} } \right) - \mathop {\min }\limits_{{e{ } \in { }E}} \left( {{\text{PR}}_{e} } \right)$$

#### Equal opportunity: measured via recall range (***R***_Recall_)

The remaining fairness metrics equalize some aspects of model performance. As introduced in Sect. 2.2.3, equal opportunity equalizes false negatives in that an equal proportion of high-risk offenders in each racial group “get away.” The statistical range of recall scores between racial groups can be used to measure its implementation [56]:

8 $$R_{{{\text{Recall}}}} = \mathop {\max }\limits_{{e{ } \in { }E}} \left( {{\text{Recall}}_{e} } \right) - \mathop {\min }\limits_{{e{ } \in { }E}} \left( {{\text{Recall}}_{e} } \right).$$

#### Predictive parity: measured via precision range (***R***_Precision_)

As introduced in Sect. 2.2.3, predictive parity equalizes false positives between racial groups in positive predictions. This means that an equal proportion of low-risk offenders is incorrectly labeled as high risk and thereby receives a police intervention. The statistical range of precision scores between racial groups can measure its implementation [56]:

9 $$R_{{{\text{Precision}}}} = \mathop {\max }\limits_{{e{ } \in { }E}} \left( {{\text{Precision}}_{e} } \right) - \mathop {\min }\limits_{{e{ } \in { }E}} \left( {{\text{Precision}}_{e} } \right).$$

#### Treatment equality (***R***_Errror_balance_)

Treatment equality was introduced in Sect. 2.2.3 and ensures that each racial group suffers the same ratio of false negatives and false positives, as defined below [19]:

10 $${\text{Error}}\_{\text{balance}} = \frac{{{\text{FN}}}}{{{\text{FP}}}}.$$

To measure the extent of implementation, the statistical range of error balance can be used, as defined below [56]:

11 $$R_{{{\text{Error}}\_{\text{balance}}}} = \mathop {\max }\limits_{{e{ } \in { }E}} \left( {{\text{Error}}\;{\text{balance}}_{e} } \right) - \mathop {\min }\limits_{{e{ } \in { }E}} \left( {{\text{Error}}\;{\text{balance}}_{e} } \right).$$

### Implementing fairness

#### Justifying threshold setting over other techniques to implement fairness

We will implement fairness via a threshold-setting procedure: through fairness constraints [57], a post-processing technique that maximizes performance under fairness. This technique “works” by leveraging the confidence score. In other words, the model outputs a confidence score for each prediction, where, if it exceeds a certain threshold, the model flags the individual as at risk of recidivism. This sort of threshold-setting technique may require the model to use custom decision thresholds for each protected group [58] such that a fairness definition can be achieved. For example, to implement statistical parity, a model may be required to flag Whites as at risk of recidivism if it is 50% confident, whereas that threshold may be raised to 80% for Blacks.

This sort of threshold-setting procedure is just one example of how the fairness scale can be implemented. Other techniques exists, and they are often divided into preprocessing, in-processing, and post-processing techniques [42]. Preprocessing techniques often occur on the dataset itself; they include techniques like rebalancing and perturbing both outcome and sensitive group membership. In-processing techniques typically occur within the algorithm as part of the model creation process; it can involve rewriting an algorithm to incorporate a fairness penalty, which is often difficult. Post-processing techniques occur after a model is created, with one of the best-known techniques being setting custom decision thresholds for each protected group [58].

We chose this specific threshold-setting technique [57] first because it is highly flexible—it is model agnostic, meaning it can be applied to *all* models outputting a confidence score. Second, it is very easy to understand, facilitating explainability to stakeholders and thereby adoption. Third, it is very easy to update: It does not require the model to undergo retraining, which can be an expensive and regular occurrence in the real world where the sociopolitical environment and regulations are constantly changing. The flexibility, explainability, and ease-of-updating associated with this approach justify why we select it over other techniques. Moreover, we were able to use this technique to implement all definitions of fairness (see Sect. 4.1), and this efficacy further justifies its use.

Finally, studies of recidivism forecasting typically do not debias their model or implement a fairness definition. Of the reviewed recidivism forecasting studies (Sect. 2.1), only two have examined fairness [32, 40]; of these two, only one has implemented a fairness definition via a calibration procedure [40]. Thus, it is difficult to compare our approach to other approaches used in the reviewed forecasting literature because there is relatively little precedent to draw from. In this regard, this threshold-setting procedure may create precedent in a subfield where little precedent exists, further adding to this investigation’s contribution.

#### Implementation details

Numerous steps are undertaken to implement the threshold-setting procedure proposed by [57]. Namely, we first assess a model’s performance at every possible decision threshold, separated by race. Let *M* represent the set of fairness metrics, defined as *M* = {*R*_PR_, *R*_Recall_, *R*_Precision_, *R*_Errror_balance_}. For each threshold *T* ∈ [0,1], we compute (i) a fairness metric and (ii) the model’s overall score for each racial group *e* ∈ *E.* We then identify the set of thresholds *T*_Fair_ that satisfy our fairness constraint *for each individual metric*, which must be less than our tolerance value ϵ of 0.01 (see Sect. 3.6.1). Finally, from these thresholds, we select the combination that produces the best-performing model for each individual metric. Best performance is defined by (i) computing the model’s *F*_1_ score for each racial group at that threshold, (ii) summing together those *F*_1_ scores, weighing each race’s score equally, and (iii) selecting the model with the highest *F*_1_ score sum.

Mathematics, for each fairness metric *m* ∈ *M,* define the set of fairness thresholds unique to the metric $T_{{{\text{Fair}}}}^{m}$ as:

12 $$T_{{{\text{Fair}}}}^{m} = {\text{\{ }} T \in \left[ {0.1} \right] {|} m\left( T \right) < 0.01\}$$

Thus, for each fairness metric *m* ∈ *M,* we now have the corresponding set of fairness thresholds $T_{{{\text{Fair}}}}^{m}$. Next, let *F*_1*e*_(*T*) denote the *F*_1_ score for each racial group *e* ∈ *E* at threshold *T*. We seek to find the optimal threshold $T_{{{\text{opt}}}}^{m}$ from the fairness-constrained threshold set $T_{{{\text{Fair}}}}^{m}$ that maximizes the sum of the *F*_1_ score for all racial groups:

13 $$T_{opt}^{m} = \mathop {\text{arg max}}\limits_{{T \in T_{{{\text{fair}}}}^{m} }} \mathop \sum \limits_{{e \in { }E}} F_{1e} \left( T \right) .$$

Ultimately, $T_{{{\text{opt}}}}^{m}$ will yield the optimal threshold that (i) satisfies the fairness metric *m* while (ii) maximizing model performance.

**Fig. 5:**
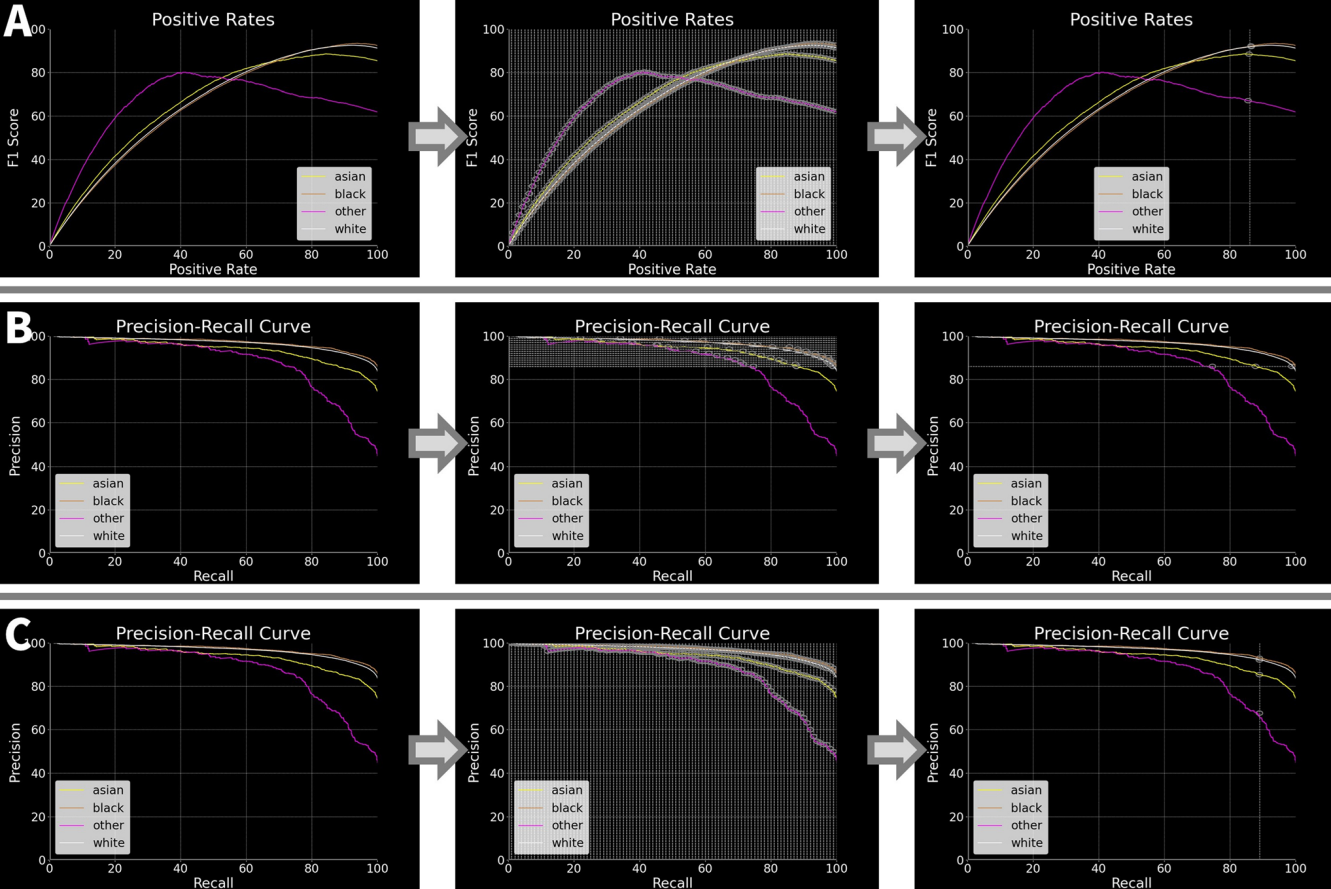
Algorithmic implementation of fairness constraints, with results plotted. Results are for general recidivism, in which the thresholds of our best-performing model were optimized to achieve a particular fairness constraint. Results of Eq. (12) appear in the center graphs, whereas equivalent for Eq. (13) appears on right-most graphs. All graphs depict three phases: (i) an initial graph showing the metric to equalize, (ii) the algorithm identifying all thresholds for which the statistical range of that metric between racial groups is less than the threshold (0.01), indicating fairness, and (iii) the ultimate threshold selected, which produced the highest *F*_1_ score sum between races, weighted equally. **A** The implementation of statistical parity, accomplished by equalizing positive rate between races while maximizing the *F*_1_ score sum. **B** The implementation of predictive parity, accomplished by equalizing precision between races while maximizing the *F*_1_ score sum. **C** The implementation of equal opportunity, accomplished by equalizing recall between races while maximizing the *F*_1_ score sum. Thresholds were omitted from the graphs for simplicity. All graphs were obtained from fold #0 of the gradient boosting machine that implemented fairness through unawareness in which it could not explicitly use race

Finally, Eqs. (12) and (13) were implemented using a Python algorithm, whose output is graphically illustrated via Fig. 5. Figure 5 suggests that our equations were successfully implemented. Namely, Fig. 5 illustrates Eq. (12) identifying all possible decision thresholds in which a fairness constraint is satisfied (center graphs), as well as Eq. (13) in which it identified the decision thresholds that produced the highest *F*_1_ score sum for a particular fairness constraint (right-most graphs). Once these thresholds were identified, the model was configured to only predict recidivism for a particular racial group if the probability of recidivating was greater than that racial group’s decision threshold, thereby ensuring (i) maximum model performance while (ii) satisfying a fairness definition.

## Results

Results were divided into two sections. First, we identified the best-performing model for both general and violent recidivism *across all possible decision thresholds*, as identified via AUC score. This is consistent with past forecasting studies (see Table 3), and it allows our model to be compared to existing state-of-the-art instruments.

Second, once a best-performing model is identified, we then assess its (i) performance and (ii) fairness *at one specific threshold.* Once its performance is assessed, we then configure the model so that it implements each fairness definition described in the fairness scale. Each fairness definition can be successfully engineered into the model; however, the implementation of each definition inflicts technical and semantic trade-offs onto the model, a finding consistent with the literature [22, 42, 43]. Thus, we ultimately present a selection of models practitioners can use, with the practitioners being responsible for the ultimate decision (see Sect. 5.3 for recommendations).

### Best-performing models across all thresholds

#### Identifying best-performing models

**Fig. 6:**
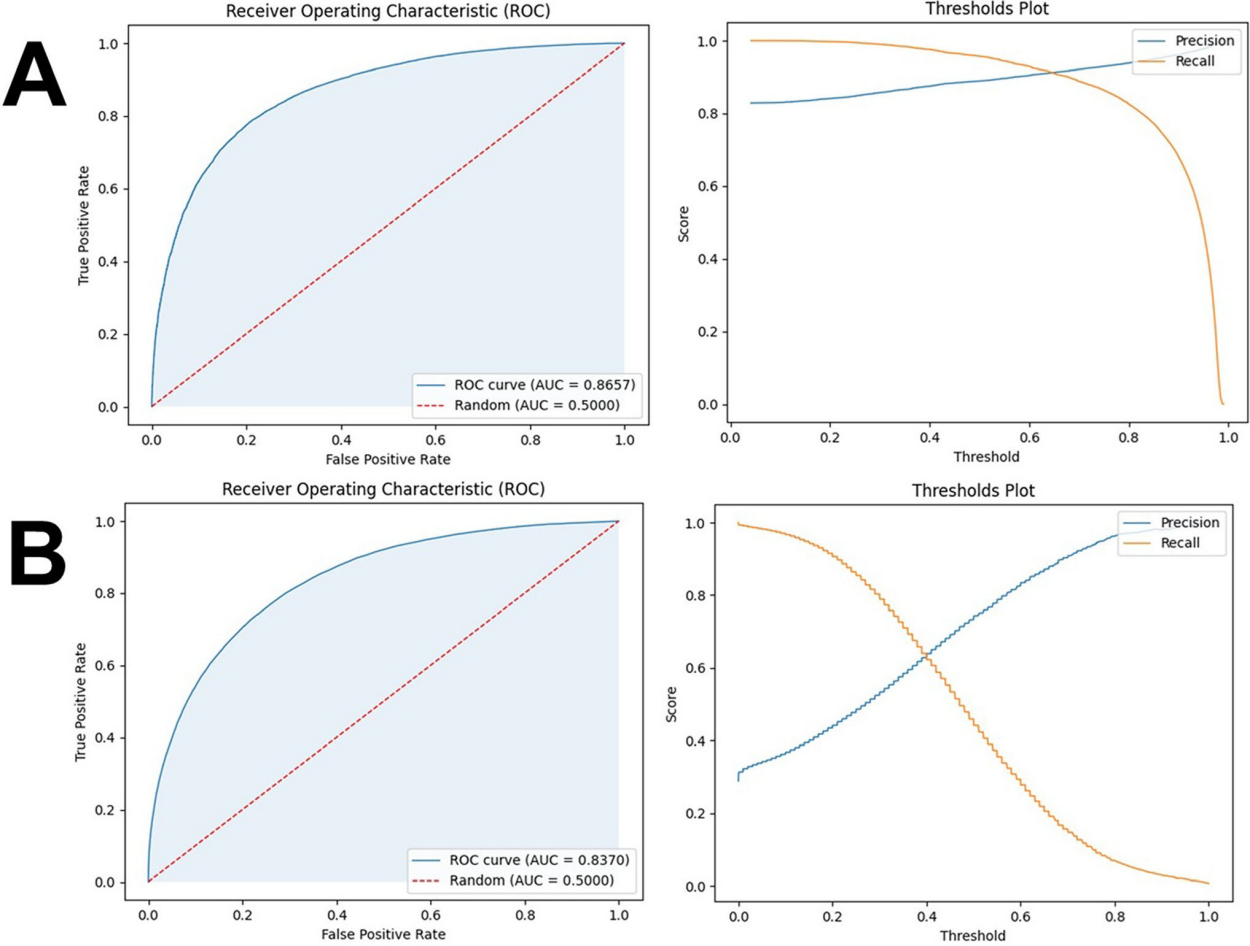
Illustration of model performance across thresholds. **A** The receiver operator characteristics curve and a threshold plot for the gradient boosting machine, the model that could best predict general recidivism as determined by AUC score. **B** The equivalent for the random forest, the model that could best predict violent recidivism. Results were taken from Fold #0

The performance of all 12 models appears in Table 10, which contains the performance summary of the 6 models that can predict violent recidivism and the 6 that can predict general recidivism. Our best-performing model was the gradient boosting machine and the random forest for general and violent recidivism, respectively, as they output the highest AUC scores. Recall, precision, and specificity were taken at the threshold that maximized recall score while requiring a minimum precision score of 85% to minimize the harm to false positives. If this minimum precision score could not be met, then the threshold that maximized the model’s precision score was chosen. The performance of both models appears in Fig. 6.

**Table 10:** Performance summaries of machine learning models that forecast recidivism

Model	AUC (SD)	Recall (SD)	Precision (SD)	Specificity (SD)
*General recidivism*				
Gradient boosting machine	0.8660 (0.0005)	0.9567 (0.0009)	0.8894 (0.0003)	0.4269 (0.0032)
Multilayer perceptron	0.8649 (0.0010)	0.9601 (0.0028)	0.8870 (0.002)	0.4105 (0.0105)
Random forest	0.8506 (0.0012)	0.9474 (0.0004)	0.8870 (0.0005)	0.4182 (0.0056)
Linear discriminant analysis	0.8443 (0.0012)	0.5584 (0.0025)	0.9637 (0.0006)	0.8985 (0.0024)
Logistic regression	0.8184 (0.0030)	0.6259 (0.0219)	0.9543 (0.0013)	0.8554 (0.0084)
Elasticnet	0.7508 (0.0033)	0.7131 (0.0203)	0.9137 (0.0028)	0.6751 (0.0222)
*Violent recidivism*				
Random forest	0.8375 (0.0018)	0.2537 (0.0178)	0.8532 (0.0022)	0.9823 (0.0014)
Gradient boosting machine	0.7784 (0.0015)	0.0437 (0.0029)	0.8507 (0.0006)	0.9969 (0.0002)
Multilayer perceptron	0.7777 (0.0026)	0.0465 (0.0184)	0.8505 (0.0001)	0.9967 (0.0013)
Linear discriminant analysis	0.7512 (0.0014)	0.0107 (0.0024)	0.8550 (0.0053)	0.9993 (0.0002)
Logistic regression	0.6987 (0.0052)	0.0329 (0.0124)	0.6609 (0.0427)	0.9929 (0.0034)
Elasticnet	0.5862 (0.0014)	0.4903 (0.2742)	0.3800 (0.0671)	0.6297 (0.2071)

Models are sorted by descending AUC score

Both models appear to detect general and violent recidivism remarkably well. The gradient boosting machine can detect 95.67% of offenders who will commit another crime generally, with 88.94% precision, whereas the random forest can detect 25.37% of offenders who will commit a violent crime, with 85.32% precision. Both the random forest and gradient boosting machine are remarkably precise in that most of the predictions they generate are correct—that is, if they predict someone will recidivate, that person will recidivate over 85% of the time. However, the random forest is not able to detect as many cases of violent recidivism relative to the gradient boosting machine’s general recidivism forecasts because its recall score is significantly lower. Yet, this may not be a problem because the sample size is sufficiently large: It can still correctly identify *several thousand individuals* who will recidivate (see suppl. Table S5) despite the low recall score, giving police an ample sample size of individuals whose crime can be prevented. Regardless of these discrepancies, both models achieved an overall AUC score that indicates “excellent” performance [37].

#### Comparison to state-of-the-art

Next, we compare our models to other state-of-the-art instruments in Table 11. Our models appear to represent the new state-of-the-art: The general recidivism model exhibits an AUC score 0.0460 points higher than the previous best-performing model, whereas the equivalent for violent recidivism is 0.0675. These are noticeable performance boosts that are within the range of what is expected.

**Table 11:** Descriptive statistics summarizing AUC values of other recidivism studies

Recidivism type (sample)	*M*	SD	Median	25th/75th percentile	Mode	Min	Max
General recidivism (*n* = 7)	.75	.05	.75	.70/.78	.78	.69	.82
Violent recidivism (*n* = 4)	.75	0.03	.75	.73/.77	N/A	.71	.77

Statistics exclude current results. Results were taken from all eight studies that reported an AUC score for general and/or violent recidivism. However, some studies reported scores for both general and violent recidivism; hence, the total exceeds eight

The success of these models can be attributed to the dataset we used, as well as the preprocessing undertaken. Namely, other forecasting studies used datasets that were highly localized both temporarily and geographically—that is, they belonged to a specific jurisdiction, and they only spanned a relatively small number of years. In contrast, the PNC contains conviction records for the entire UK. This large geographical and temporal range allowed us to recreate criminal history in a way that was completer and more holistic than past datasets, and this valuable information almost certainly boosted the predictive validity of our models. Moreover, the devised criminological predictors almost certainly boosted performance, as many of these predictors are unique to this investigation.

### Implementing fairness

#### Overview

Next, we debias our best-performing models by implementing fairness as it appears in the fairness scale. Namely, we take the best-performing model for both general and violent recidivism and calculate: (i) performance and (ii) fairness metrics on each. Then, we implement each definition of fairness using fairness constraints. First, we implement our level 1 definition—fairness through unawareness—in which we retrain our best-performing models so that they cannot explicitly use the protected attribute which, in this case, is the race feature; performance and fairness metrics on this model are calculated. Second, we impose fairness constraints on our “fairness through unawareness” models such that they implement a level 2 fairness definition; once these constraints are implemented, we consider it a new model. Thus, this process resulted in a total of 12 models: one baseline model, one model that implements fairness through unawareness by itself (level 1), and four models that each implement their own level 2 definition. Thus, each model that implemented a level 2 definition exhibits two different definitions of fairness: (i) fairness through unawareness, as this was the base model under which the fairness constraint was implemented, and (ii) the level 2 definition it implemented. Results appear in Tables 12 and 13, with full results separated by race appearing in suppl. Table S7.

**Table 12:** General recidivism models: performance and fairness summaries

Model	Performance			Fairness			
	*F*_1_ (SD)	Recall (SD)	Precision (SD)	*R*_PR_ (SD)	*R*_Recall_ (SD)	*R*_Precision_ (SD)	*R*_Error_balance_ (SD)
*No fairness implementation* Baseline model	0.9219 (0.0005)	0.9567 (0.0009)	0.8894 (0.0003)	0.4606 (0.0162)	0.1769 (0.0151)	0.0768 (0.0209)	0.9521 (0.2646)
*Level 1: No explicit discrimination* Fairness through unawareness	0.9213 (0.0008)	0.9565 (0.0009)	0.8885 (0.0007)	0.3330 (0.0194)	0.0956 (0.0152)	0.1828 (0.0164)	0.1870 (0.0365)
*Level 2: equalize outcome* Statistical parity	0.9212 (0.0009)	0.9548 (0.0054)	0.8899 (0.0033)	**0.0092 (0.0004)**	0.0475 (0.0074)	0.3750 (0.0187)	0.7609 (0.1531)
*Level 2: equalize performance* Equal opportunity	0.9124 (0.0048)	0.9183 (0.0177)	0.9069 (0.0081)	0.2023 (0.0327)	**0.0077 (0.0027)**	0.2533 (0.0240)	0.8308 (0.2925)
Treatment equality	0.9212 (0.0009)	0.9548 (0.0054)	0.8899 (0.0033)	0.0054 (0.0098)	0.0871 (0.0062)	0.1761 (0.0220)	**0.0063 (0.0026)**
Predictive parity	0.9192 (0.0015)	0.8626 (0.0044)	0.9836 (0.0026)	0.5786 (0.0241)	0.2608 (0.0262)	**0.0095 (0.0002)**	2.0804 (0.2895)

The baseline model is the gradient boosting machine described in Sect. 4.1, with fairness metrics calculated. The fairness through unawareness is the same as the baseline model, except it was not able to explicitly use race. The level 2 models are variations of the level 1 model that each implement their own level 2 definition of fairness, as implemented by the fairness constraint procedure described in Sect. 3.7. Bold indicates that a model satisfies a definition of fairness. *F*_1_ score is a model’s overall performance assessment at a specific threshold. *R*_PR_ measures our implementation of statistical parity; *R*_Recall_ measures our implementation of equal opportunity; *R*_Precision_ measures our implementation of predictive parity; *R*_Error_balance_ measures our implementation of treatment equality

**Table 13:** Violent recidivism models: performance and fairness summaries

Model	Performance			Fairness			
	*F*_1_ (SD)	Recall (SD)	Precision (SD)	*R*_PR_ (SD)	*R*_Recall_ (SD)	*R*_Precision_ (SD)	*R*_Error_balance_ (SD)
*No fairness implementation* Baseline model	0.3908 (0.0207)	0.2537 (0.0178)	0.8532 (0.0022)	0.0660 (0.0081)	0.1554 (0.0168)	0.0452 (0.0273)	27.4831 (9.0561)
*Level 1: no explicit discrimination* Fairness through unawareness	0.3885 (0.0135)	0.2517 (0.0113)	0.8525 (0.0031)	0.0586 (0.0035)	0.1545 (0.0152)	0.1633 (0.0905)	17.0536 (3.1678)
*Level 2: equalize outcome* Statistical Parity	0.6341 (0.0040)	0.6690 (0.0261)	0.6037 (0.0153)	**0.0080 (0.0013)**	0.2452 (0.0207)	0.3317 (0.0219)	1.2197 (0.1564)
*Level 2: equalize performance* Equal opportunity	0.6366 (0.0039)	0.7122 (0.0615)	0.5804 (0.0387)	0.2092 (0.0205)	**0.0079 (0.0005)**	0.1910 (0.0316)	0.3383 (0.1266)
Treatment equality	0.6257 (0.0085)	0.7007 (0.1268)	0.5865 (0.0811)	0.2628 (0.0682)	0.0993 (0.0151)	0.1191 (0.0279)	**0.0257 (0.0198)**
Predictive parity	0.6345 (0.0140)	0.7790 (0.0565)	0.5392 (0.0416)	0.4035 (0.1033)	0.2818 (0.0544)	**0.0056 (0.0013)**	0.7827 (0.0705)

Table is the same as Table 12, except the baseline model is the random forest model that can forecast violent recidivism, as described in Sect. 4.1

#### Level 2 fairness definitions require trade-offs that prevent simultaneous implementation

Results indicate that implementing a fairness constraint did not majorly affect the performance of the model overall. This was likely the result of the careful fairness constraint implementation we undertook, which was specifically geared to maximize model performance under that constraint (see Sect. 3.7). However, the models differ significantly regarding fairness metrics. Specifically, the baseline models—the model that explicitly used race—contradicted many fairness definitions. The violent recidivism model, for example, flagged 9.06% of Blacks as high risk of recidivism whereas only 3.01% of Asians were flagged for the equivalent (see suppl. Table S7), violating statistical parity; it could detect recidivism approximately 20-percentage points better for Black and White offenders than Asians, violating equal opportunity (see *R*_Recall_ in Table 13 for summary, suppl. Table S7 for recall scores for each race); and the errors were unbalanced such that Asians suffered dramatically more false negatives than all other races, violating treatment equality (see *R*_Error_balance_ in Table 13 for summary, suppl. Table S7 for error balance for each race). These differences were typically ameliorated somewhat when fairness through unawareness was implemented—when the model could not explicitly use race—yet major differences persisted. Moreover, this example is specific to the violent recidivism model, yet similar findings hold for general recidivism.

**Fig. 7:**
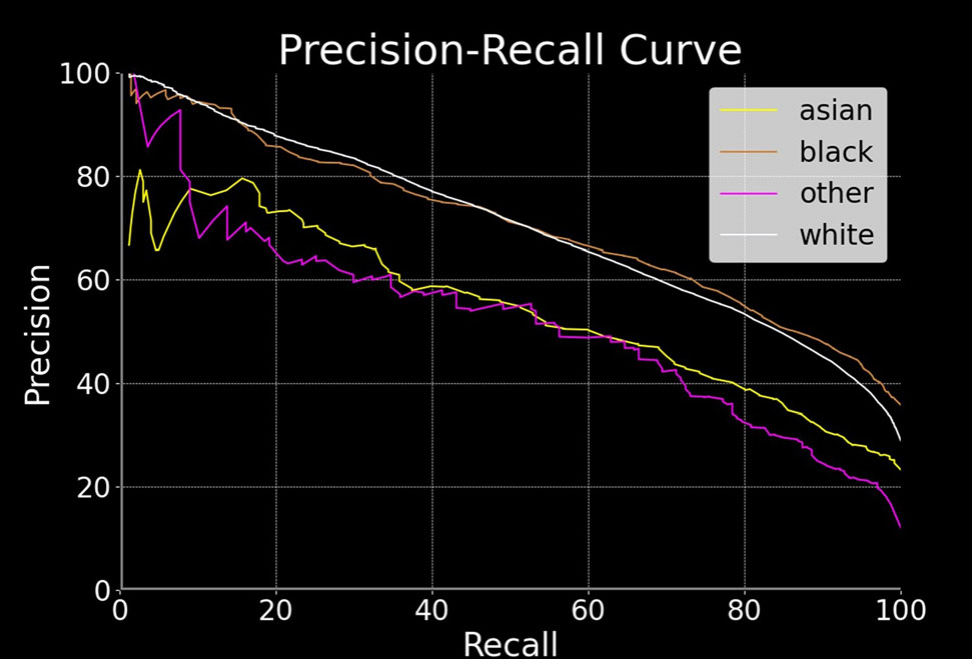
Precision–recall curve of random forest that can predict violent recidivism. Figure was created using the random forest that implements fairness by unawareness in which it does not explicitly use race. Figure illustrates that the precision–recall curves for each race never intersect, highlighting the technological difficulty of implementing equal opportunity (recall) and predictive parity (precision) between models

We used level 2 fairness definitions—and their corresponding fairness constraints—to remedy each of these inequalities. However, only one could be nicely implemented at a time; the simultaneous implementation of multiple level 2 fairness definitions was difficult due to large base-rate differences in the data, a finding echoed in the literature [42, 43]. Perhaps the best illustration of these incompatibilities appears in the precision–recall curve in Fig. 7, which illustrates precision and recall across every possible decision threshold, separated by race, for violent recidivism. Equalizing precision (predictive parity) and recall (equal opportunity) between races would be ideal, as it would satisfy two level 2 definitions simultaneously. However, this ideal is infeasible: The precision–recall curves of the four races never intersect, even factoring in the tolerance ϵ. Thus, one is left with a difficult trade-off. Namely, is it more important to equalize predictive parity (precision) such that an equal proportion of low-risk offenders are incorrectly labeled high risk for each race; or is equal opportunity (recall) perhaps more important such that the model can detect recidivism equally well between races? This argument is specific to predictive parity and equal opportunity; yet, a similar argument can be made for combinations of the four level 2 fairness definitions.

Thus, this finding echoes a common finding in the fairness literature: The simultaneous implementation of these fairness metrics is incompatible; each inflicts a technical trade-off; and that trade-off requires one to make a semantic-political decision [42]. Indeed, whenever one level 2 definition was implemented, it often came at the price of exacerbating the other three. For example, we could implement equal opportunity such that our model could detect recidivism equally well between races (*R*_Recall_ < 0.01); however, this exacerbated the predictive parity violation such that some races experienced significantly more false positives than others (*R*_Precision_ = 0.2023 for general, 0.2092 for violent). Moreover, we could achieve predictive parity such that each race suffers the same proportion of false positives in positive predictions (*R*_Precision_ < 0.01)—such that an equal proportion of low-risk offenders is incorrectly flagged as high risk; however, this caused our model to detect recidivism dramatically better for some races than others, exacerbating the equal opportunity violation (*R*_Recall_ = 0.2608 for general, 0.2818 for violent). Finally, we could override preexisting patterns in this criminal justice dataset to force the model to flag an equal proportion of each race as high risk, satisfying statistical parity (*R*_PR_ < 0.01); however, this exacerbated the other three level 2 definitions of fairness.

Crucially, results show that all five fairness definitions are implementable, but not simultaneously. Thus, practitioners may need to select one level 2 fairness definition to implement. When applied to the real world, this simultaneous implementation incompatibility suggests that all models should perhaps be presented to criminal justice practitioners, and local context should decide which metric to implement (see Sect. 5.3).

## Discussion

In the UK, nearly half of all convicted offenders will commit another crime one year after release [59], and these crimes impose serious financial, resource, and emotional costs on victims, communities, and the criminal justice system overall (see suppl. introductory material) [2–4]. If the criminal justice system can forecast recidivism, then it could prevent both these crimes and their serious costs; yet, in its nearly 100-year history of attempting to do so, it has achieved mixed success [9, 11]. In this investigation, we first created two machine learning models—one that forecasts general recidivism, another that forecasts violent recidivism—that outperform existing state-of-the-art instruments. Second, we debias our best-performing models so that they implement at least one definition of fairness. The performance of our debiased models is sufficiently high such that police can consider using them to prevent recidivism. Thus, we discuss how police can carefully move toward deploying this models (Sects. 5.1–5.4), before proposing further academic directions (Sect. 5.5) and explicating our scientific contributions (Sect. 5.6).

### Potential to reduce crimes while managing false positives

Police can use our models to predict and thereby prevent recidivism. Specifically, police can use our models to generate a list of recently released offenders who will likely recidivate. They can then leverage a variety of interventions to prevent these offenders from committing crimes. For example, police can launch an intervention like a nudge in which they send a text message warning the offender not to offend; enact focused deterrence in which they visit the offender, informing him/her that future offending will result in swift action; and issue protection or risk orders, ordering the offender to avoid certain situations likely to evoke a crime, among other interventions. While other use cases exist (see introduction), this is the primary envisioned use case of our model: It can help police identify recently released convicted offenders who are high risk so that their recidivism can be prevented.

The specifics of the intervention will be developed in collaboration with a yet-to-be-identified partner agency; thus, the intervention is not yet finalized. However, multiple safeguards will be implemented to minimize harm to false positives. In other words, our models outperform the existing state-of-the-arts, and they are sufficiently precise such that the overwhelming majority of names they output will be of offenders that will actually commit another crime (precision = 0.8894 and 0.8532 for general and violent recidivism, respectively). However, there will inevitably be some low-risk offenders who are incorrectly labeled as high risk and thereby wrongly given a police intervention, known as a false positive. These police interventions can result in serious societal gains because they can prevent lives from being ruined by a future crime. However, police interventions can also harm society if they adversely affect the lives of these false positives. Thus, safeguards that guard false positives are crucial.

To this end, we first recommend the agency we work with limit the intervention they conduct to effective albeit less-extreme ones. In other words, less-extreme interventions like nudges and focused deterrence have been shown to effectively prevent future crimes [26, 27] while leaving lasting adverse effects on those they are conducted on, if any at all. Thus, we will insist that police use these less-extreme interventions over the higher-impact equivalents that can inflict potentially life-changing consequences—like protection orders or arrests.

Second, we built in restrictions into our model on who can receive a forecast. In other words, for police to generate a recidivism forecast, an individual must be a convicted offender who has been entered into the PNC; the model cannot make predictions from everyday people who have never received a conviction because they will not be eligible for this sort of PNC entry. Thus, when a false positive is generated, it will represent an offender with at least one conviction. These are people who have already had a thorough interaction with the criminal justice system. Thus, in no way will predictions stemming from our model cause everyday people to receive a police intervention, thereby greatly limiting the potential harm to false positives.

### Human-centered deployment: proposed design

Third, we propose a human-in-the-loop deployment design so that a human practitioner can catch and override false positives. Specifically, humans are known to succumb to a sort of algorithmic bias in which they uncritically accept a model’s output [22]. Algorithmic bias may prevent humans from correcting false positives because if our model makes an egregious mistake—such as flags a clearly low-risk offender as high risk—a human may uncritically accept this output and assign this low-risk offender a police intervention.

Fortunately, automation bias—and its effect on false positives—can be combated via a particular human-in-the-loop deployment design [60]. In this framework, both the practitioner and the machine learning model independently forecast recidivism using a similar feature set derived from the preprocessed PNC data, which includes variables such as the offender’s age, gender, and criminal history. Among these, criminal history, alongside age and gender, emerges as one of the strongest predictors of future offending in our models. This information allows practitioners to make informed predictions in a manner similar to our model.

**Fig. 8:**
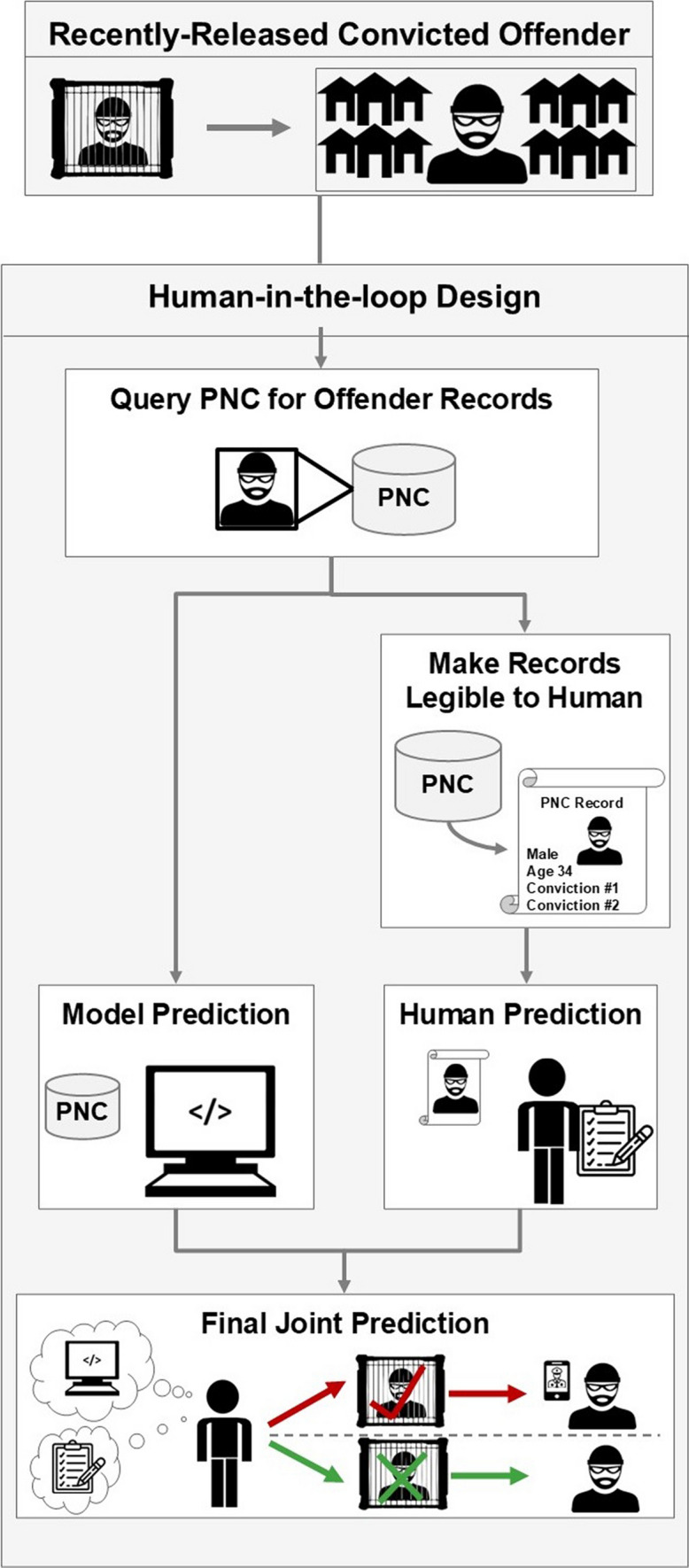
Proposed human-in-the-loop deployment design. Design is intended to showcase a joint human-model decision in deciding who receives a police intervention. This design is intended to enforce decision accountability while serving as a check against false positives. Gray box indicates category, whereas white box indicates a specific step. Bottom box (“Final Joint Prediction”) is intended to show how a practitioner—with both a human and model-generated recidivism forecast—can either allow or disallow a police intervention, which, in this case, is a text message

Independent prediction is essential because it will force the practitioner to analyze the specifics of each case by himself/herself, without the influence of the model. After forming an initial judgment, the practitioner can *then* receive the model’s output. This independent prediction generation, therefore, will require the practitioner to have already worked through the offender’s case such that, if they receive a surprising model prediction, they can challenge it. In this way, the model serves as a supplementary source of intelligence, enhancing rather than replacing the practitioner’s judgment. This deployment strategy is illustrated in Fig. 8.

This human-in-the-loop design is preferable because it maintains accountability—the practitioner, not the machine, will remain responsible for the ultimate decision to undertake the police intervention. Moreover, this also serves as a check against false positives because, if a clearly low-risk offender is labeled as high risk by the model, the practitioner will be able to override its output. Of course, the human-in-the-loop deployment design will not completely remediate algorithmic bias [60]; however, it can combat it more effectively than conventional deployment designs in which the model automatically generates a prediction without requiring the human to wrestle with specifics.

### Addressing structural bias

#### Within models

There are deep structural and societal biases throughout the criminal justice system. For example, over-policing a minority neighborhood can inflate crime detection in those neighborhoods; socially disadvantaged people are unlikely to have access to a skilled attorney; and criminal justice actors can be explicitly and implicitly biased against certain groups, among other biases (e.g., [20, 28]). All these structural biases have undoubtably biased who appears in the conviction data that our models were trained on. Specifically, these biases affected the *base-rate recidivism differences* between protected groups. In other words, certain demographic groups are more likely to receive a conviction than others, and it is unclear to what extent these differences reflect structural biases in the criminal justice system [28] versus a genuine difference in crime [46].

These base-rate recidivism differences affect forecasting models because these models will recreate historical patterns in the data [28]. For example, if the training data suggest that 80% of Blacks recidivate whereas only 20% of Asians, a machine learning model will attempt to recreate this 60-percentage point base-rate recidivism difference in its predictions. Insofar as much as this base-rate difference represents structural bias, the model would be recreating structural bias.

It is critical to note that this structural bias concern is not unique to our approach; structural biases exist in *all* criminological datasets drawing from a partial judicial system [42]. Indeed, all models presented in the literature review (Sect. 2.1) drew from a dataset that inherited some form of structural bias, meaning each of these published forecasting models may be recreating some form of this bias in their predictions. However, our approach is unique in that we have addressed structural bias more thoroughly than these reviewed works.

First, we were careful to use conviction data rather than arrest data. Conviction data filter out non-convictable offenses, which disproportionately impact certain demographics through false arrests or unfounded charges [61]. The stringent legal standard for conviction ensures that the training data are composed exclusively of individuals judicially determined to have committed a crime. Thus, non-convictable offenses do not appear in our data, thereby preventing their embedded structural biases from being recreated by our models.

Second, when implementing the fairness scale, we removed a protected characteristic from the models—race—so that they could no longer explicitly use this feature in their predictions. This sort of debiasing does not fully address structural biases because it can appear in more implicit forms [19]. Yet, it does address explicit bias because some criminal justice practitioners may overtly use race in their conduct [62] despite legal prohibitions suggesting otherwise. Our models cannot explicitly use race, thereby supporting the assertion that they may be less biased than the status quo.

Third, we simultaneously implemented more structural bias correction techniques than the reviewed studies. In other words, our debiased models (i) used conviction data, (ii) were prohibited from explicitly using a protected characteristic via the implementation of fairness through unawareness, and (iii) implemented a level 2 fairness definition that either equalized outcome or some aspect of performance, thereby addressing structural biases arising from base-rate recidivism differences. While the reviewed studies have undertaken (i)-(iii) (e.g. [40],), our study is unique in that we have undertaken all three *simultaneously*. This simultaneous implementation allows us to implement multiple fairness definitions, thereby correcting multiple forms of structural bias in a way not otherwise present in the literature.

Fourth, our study used a more rigorous disparity tolerance value relative to past work. In other words, to be considered fair, the tolerance value ε is often set to 20-percentage points (see Sect. 3.6.1) [32, 56]. This means that a model may still be considered fair or debiased if, for example, it can detect Black recidivists 19 percentage points better than White recidivists. We viewed this conventional tolerance value as too high; thus, we lowered the value to merely one percentage point (see Sect. 3.6.1 for further review and justification). Our low tolerance value suggests that our debiased models more robustly satisfy a fairness definition relative to others, further combating the structural bias assuaged by that fairness definition’s implementation.

**Fig. 9:**
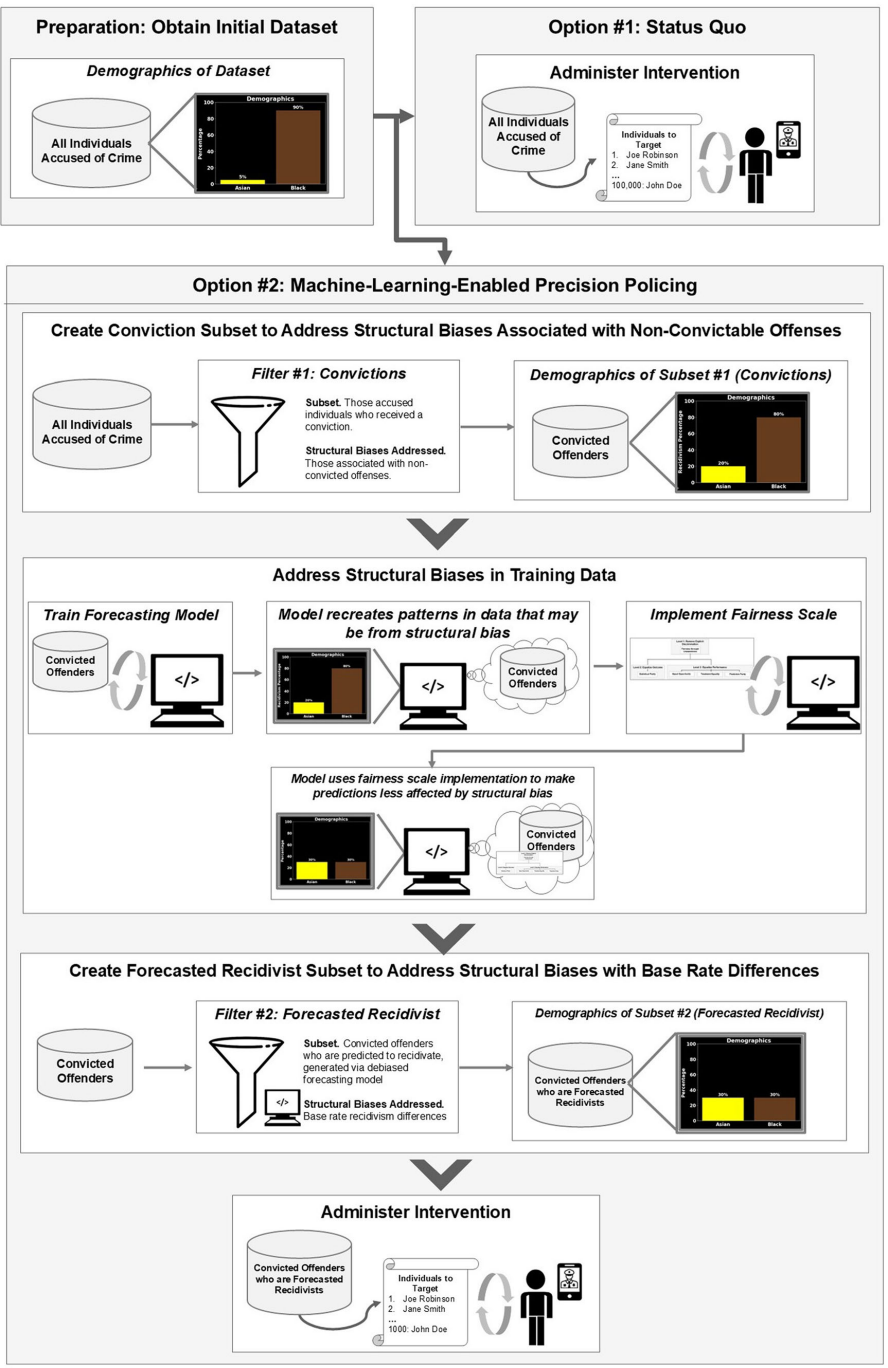
Illustration of how fairness scale implementation can correct for structural biases in base-rate recidivism differences between protected groups. In this illustration, protected groups refers to race with brown representing Black individuals and yellow representing Asian individuals within the demographic charts. The particular fairness scale implementation focuses on statistical parity. Illustration indicates that, rather than targeting all individuals accused of a crime, police can target a subset of individuals that are (i) convicted offenders (“Filter #1”) and (ii) forecasted recidivists, with forecasted recidivist being selected via a model that implements some form of statistical parity (“Filter #2”). This would result in both *fewer* people being targeted by police, and these *fewer* people may more equitably represent the population, as indicated by the various demographic graphs going from very racially imbalanced (toward top) to perfectly balanced (toward bottom)

Fifth, we were the only reviewed study that proposed implementing statistical parity. This is a more extreme form of structural bias correction than others because it “overrides” preexisting patterns in the training data such that the model’s predictions are more equitable. In other words, there are large base-rate recidivism differences between protected groups that persist even after using conviction data. These base-rate differences may reflect decades of structural bias [61]; however, we show practitioners how to configure the model such that, going forward, the model flags all races as *equally* likely to recidivate (see Sect. 4.2 and Tables 12, 13). In this regard, the decades of structural bias that affected the base-rate recidivism differences in the training data are somewhat “overridden” such that they will less affect the model’s predictions going forward. An illustration of how this may affect the criminal justice system appears in Fig. 9.

It is critical to note that implementing statistical parity will not correct all forms of structural bias. First, our implementation is controversial: Criminologists continue to debate the degree of structural bias embedded within criminal justice datasets [28, 46]; thus, the assumption embedded within statistical parity—that all protected groups should be equally likely to recidivate—is contentious. However, a less-extreme implementation of statistical parity exists such that base-rate recidivism differences between protected groups can be *reduced* in the model’s predictions to the extent that it is caused by structural bias. Thus, these base-rate differences do not need to be erased completely, and this implementation can be achieved using methods very similar to those in Sect. 3.7. Second, there are subtle yet significant forms of structural bias within the criminal justice system that cannot be corrected via modeling. Affluent individuals, for example, may be dramatically less likely to be arrested for a drug offense when compared to their low-income equivalents [28], and if they never appear in a criminal justice dataset, a model cannot flag them as at risk of recidivism. It is difficult—if not impossible—for debiasing procedures to correct for this sort of entrenched structural bias; however, a model does not need to be free from structural bias to improve the criminal justice system. It merely must exhibit less structural bias than the status quo, as encapsulated via a recent review [42]:

*“The benchmark is current practice. By that standard, even small steps, imperfect as they may be, can in principle lead to meaningful improvements in criminal justice decisions.”*

#### Within society

Once again, we have yet to find a partner agency to trial these models. However, we envision that our models can be used to implement precision policing such that police target a more specific and equitable subset of people than they otherwise would, with nudges representing a timely use case. In this case, nudges take the form of a police text message sent to individuals at risk of committing a crime; it warns the individuals not to offend. There is no obligation embedded in this text message: The recipient simply gets a warning, and there is nothing more he/she needs to do. For this reason, nudges are viewed as very low impact because they do not majorly disrupt the recipient’s life; yet, they are powerful in that they have been shown to prevent crime [27]. Thus, British police are trailing nudges in their department.

Police typically do not use precision policing to select individuals who are eligible to receive a nudge. The status quo may be that police may consider sending a nudge to *all* individuals accused of a certain crime, with a recent case study illustrated by [63]. The broad selection basis is unideal because it risks recreating structural biases: It may contain names of falsely accused individuals, which may disproportionately affect certain groups [61]; it risks sending a nudge to individuals who may never commit a crime again; and, of the people who will genuinely committed another crime, they may disproportionately be from certain low-income neighborhoods due to inflated crime detection [28], among other unideal factors.

Our models could be applied to this selection mechanism to achieve a more precise and equitable subset of individuals to target. First, our models will only identify convicted offenders (Sect. 5.1). Convicted offenders are a subset of all individuals accused of a crime. Thus, using only convicted offenders prevents falsely accused individuals from receiving the police intervention, thereby removing the structural biases contained therein. Second, from this subset of convicted offenders, an *even smaller* subset will be selected by our model: those at risk of recidivism. This second filtering is noteworthy because, if we use a model that implements a form of statistical parity, it could identify individuals in a way that less reflects structural biases than the status quo. Thus, by selecting only individuals who are (i) convicted offenders and (ii) flagged as likely to recidivate by a model that implements statistical parity, we dramatically reduce the number of people eligible for this sort of police intervention relative to the status quo, and we also ensure this selection can be more equitable.

### Toward a careful deployment

**Fig. 10:**
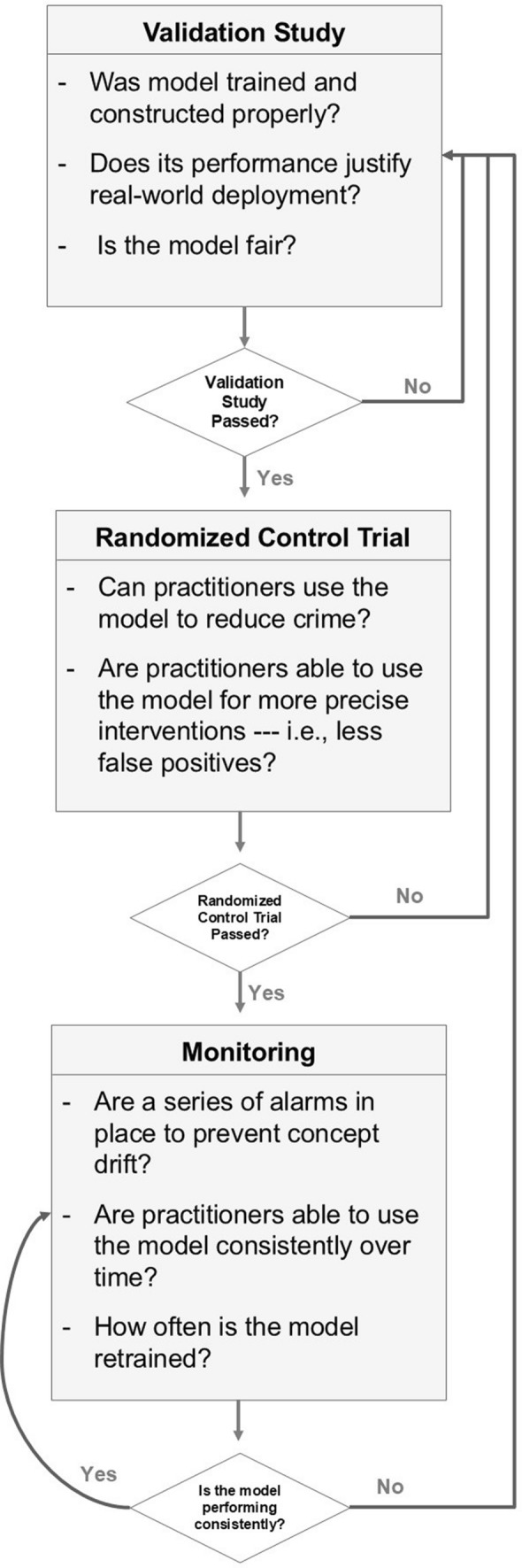
Proposed deployment pipeline. Diamonds indicate decision points, whereas gray boxes indicate a major deployment phase with key considerations

Before this sort of intervention is used, our models must be retrained on more recent data to avoid concept drift [21]. Assuming this retraining is successful, we propose deploying the models carefully through a rendition of the deployment pipeline proposed by [64] to ensure their purported benefits map onto the real world. Specifically, this deployment pipeline argues that, for police to use a machine learning forecasting model, it must go through a rigorous, three-stage process that parallels drug testing in medicine. First, a model must pass an initial validation study in which the predictive validity of the model justifies real-world deployment. Second, a model must be carefully and selectively deployed in the real world via a randomized control trial (RCT), the most rigorous form of scientific testing [65], to ensure its purported effects translate to real-world benefits. Third, once a model passes both the (i) validation study and (ii) the RCT, it can be deployed in the real world; albeit its output must be monitored over time and, if its performance dips, it gets immediately pulled from production. An adaptation of this pipeline appears in Fig. 10.

**Fig. 11:**
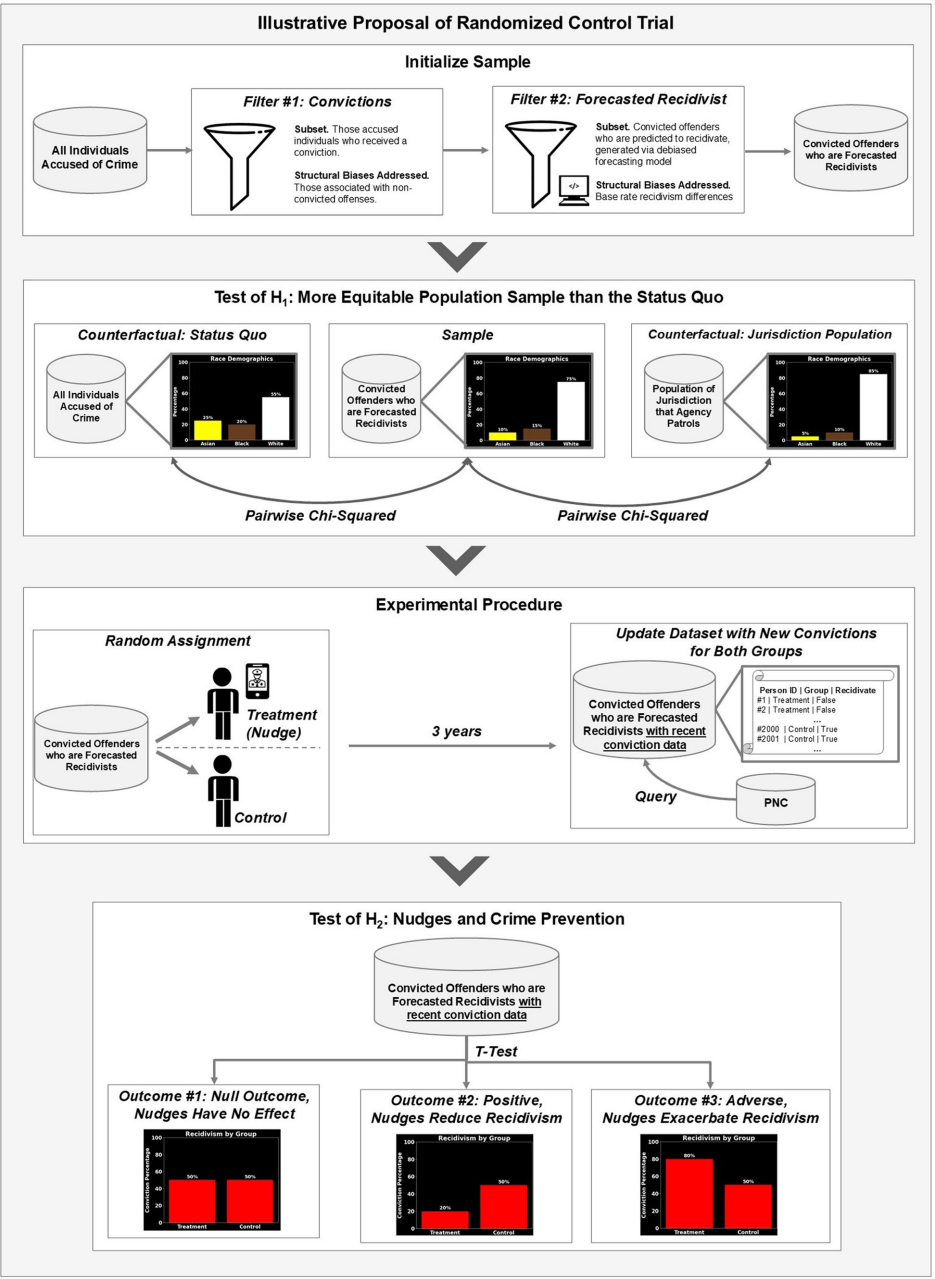
Illustrative proposal of randomized control trial. Illustration shows experimental flow, major tests of hypothesis, and what hypothetical results could look like. Illustration depicts general recidivism; yet, a very similar design can be implemented with violent recidivism

By constructing state-of-the-art forecasting models that outperform the literature, we argue that we have passed the validation study—that the predictive validity of our models can justify real-world deployment. Next, we will conduct an RCT with a yet-to-be-determined partner force. However, we would trial these models on a nudge paradigm similar to the one proposed by [63]. We are using nudges because they are timely in that they are being trialed by UK police [63], and they exhibit the potential to prevent crimes [27] while being very low impact, minimizing the potential harm to recipients. Our hypotheses are twofold. They appear below, with an overview of the proposed experimental design appearing in Fig. 11.

- H_1_: The machine learning model allows police to target a more equitable distribution of the population than they would otherwise target.
- H_2_: The nudge has a statistically significant effect on crime reduction for both (i) general crimes and (ii) violent crimes.

First, we would identify a list of individuals eligible to receive a nudge. The status quo suggests police select these individuals from a list of all individuals accused of a crime (see Sect. 5.3.2); however, we would use this list as a starting point. In other words, we would subset this list to only those who have received a conviction, and then we would run these individuals through our retrained models to identify those at risk of general and violent recidivism over a 3-year period. General and violent recidivism are defined in Sect. 2.1.1, rendering this experiment consistent with our proposed models. 3142 individuals would ultimately appear on this subset because power analysis suggests that this should be sufficient for detecting a small effect size (*d* = 0.1), assuming an alpha of 0.05, 80% power, and a two-tailed significance test [66].

These retrained models will likely implement a form of statistical parity. Thus, the predictions they generate should be used to construct a sample less structurally biased than the status quo. Therein lies the test of H_1_: We will take the demographic composition of our sample and compare it to the demographic composition of two counterfactuals: a random sample of (i) all individuals accused of crimes and (ii) the jurisdiction of the police agency oversees. The former represents the status quo, whereas the latter can serve as a proxy for what an equitable demographic distribution would look like in the absence of criminal justice bias. Using race as an example, we will conduct a chi-squared test to see if the demographics of our sample are significantly different from either counterfactual; and, if significance is detected, we will use a pairwise chi-squared with a Bonferroni correction to identify the source of this discrepancy. If our sample is more demographically similar to the population of the jurisdiction than the status quo, we will conclude that H_1_ is supported. This would be supported by the chi-squared tests under the following conditions:

- The demographics of our sample are statistically different from the demographics of the status quo: A sample from all individuals accused of a crime.
- The demographics of our sample is *not* statistically different from the demographics of a sample derived from the jurisdiction, our potential proxy for an equitable distribution.

Next, in keeping with the paradigm of a randomized control trial [65, 67], we would assign half our sample to the treatment, the other half to the control, and then we would administer a nudge in the form of a police text message. This administration will be in a manner similar to [63] in which thorough manipulation checks are in place, such as checking for a valid phone number. After the nudge is administered, we would measure whether the recipients have received a new conviction for a general or violent crime over the next 3 years. The 3-year period was chosen because it should be sufficiently long for a new conviction to appear, and it renders it consistent with our models (see Sect. 2.1.1). Moreover, we would detect new convictions via PNC queries to obviate the need for a survey-based design in which we follow-up with each individual offender.

Regarding results, if there is less crime in the treatment group than the control group for both general and violent crime, we would conclude that the nudge was successful, using a pairwise-t-test to validate this comparison. We would use this test because it tends to be sufficient for measuring crime differences in police-conducted randomized control trials [67]. Other possible results—such as a null or adverse result—are illustrated in Fig. 11.

It is critical to note that this RCT proposal is a toy example. We do not yet have a partner force who agreed to this, suggesting details and implementation specifics will almost certainly change. Moreover, we have also omitted many granular details from this illustration such as crime measurement specifications like the debate around crime prevalence, count, or harm [67]. Nevertheless, our illustration offers a robust illustration of how an RC could be conducted.

### Further research directions in the fairness scale

Regardless of real-world deployment, the fairness scale could be improved through further research. Namely, this investigation exists partially to propose the fairness scale and show that it *can* be implemented—it was not designed to evaluate the optimal way of implementing it. In other words, we chose to debias our model via a specific post-processing threshold-setting procedure [57] for reasons discussed in Sect. 3.7.1. This technique allowed us to validate the fairness scale by showing: (i) Each definition within the fairness scale is implementable and (ii) our results are highly consistent with the literature [42]. However, a better implementation technique may exist that leverages a variety of pre-, in-, and post-processing debiasing techniques. Some combination thereof can produce more optimal implementations of the fairness scale; yet, discovering this combination is well beyond the bounds of this initial validation study.

Second, we chose a strict disparity tolerance (ϵ = 0.01), requiring our models to overcome a relatively high bar to satisfy a fairness definition for reasons discussed in Sect. 3.6.1. In practice, however, the bar need not be so high; it can be lowered to tolerate more disparity, and this lowering may allow the model to achieve multiple level 2 definitions simultaneously. For example, if a more generous 20-percentage point disparity is allowed (e.g., [32]), one could potentially implement multiple level 2 definitions simultaneously, like predictive parity *and* equal opportunity. Of course, increasing disparity tolerance raises the serious question as to whether the model really satisfies a fairness definition—especially if, for example, the model generates a 20-percentage point difference in false positives for one race relative to another. Yet, a study that investigates fairness scale implementation *at different disparity tolerances* could elucidate the trade-offs in raising or lowering this tolerance.

Third, the fairness scale is a domain-specific framework for debiasing a forecasting model. It is intended to help criminologists debias a recidivism forecasting instrument in a way that addresses challenges in criminal justice datasets (see Sect. 2.2.2). Yet, in practice, it may be applicable to other fields. Indeed, the assumptions that went into the construction of the fairness scale—such as a preference for binary outputs and sparsity (see Sect. 2.2.2)—may be applicable to other domains using machine learning forecasting instruments such as finance or medicine. Further work is needed to elucidate the assumptions of these fields such that some rendition of the fairness scale can be applied, further facilitating debiasing in these fields.

### Scientific contributions

This investigation makes (i) theoretical, (ii) methodological, and (iii) deployment-related contributions to science. From a theoretical perspective, we first built upon the literature review conducted by [17], extracting a set of common parameters by which forecasting models are constructed. This extraction required resolving ambiguities in literature such as conflicting recidivism definitions and a disjoint set of evaluation metrics, among others. The resolution of these inconsistencies gave rise to a concise set of common parameters in Sect. 2.3, rendering them accessible and easy-to-implement by others designing similar forecasting instruments.

Second, we propose a fairness scale that effectively communicates the technical and semantic challenges of debiasing a model. This is crucial because fairness definitions are rarely implemented in forecasting and criminal justice. Indeed, of the eleven forecasting studies reviewed, only two implemented a fairness definition [32, 40], and even then, discussion of the semantic and technical trade-offs implied therein was limited. This may stem from the fairness literature’s reputation for complexity, as its reviews often present a long list of contradictory and domain-irrelevant metrics. Indeed, a recent review highlights just that, arguing that more work is needed to define a subset of fairness definitions relevant to a subfield [19]. In this regard, the fairness scale does just that—it provides a concise set of fairness definitions tailored to criminal justice forecasting, accounting for domain-specific challenges such as significant base-rate disparities and the disproportionate consequences of certain predictions (see Sect. 2.2). By clarifying and simplifying these complexities, the scale facilitates the practical adoption of fairness definitions that are otherwise unimplemented.

Moreover, the fairness scale helped enable us to address structural bias in a way more thorough than the reviewed studies in Sects. 2.1 and 2.2. Namely, we debiased our models through five procedures: (i) used conviction data, (ii) prevented the models from explicitly using a protected characteristic via the implementation of fairness through unawareness, (iii) *further* debiased the models in (ii) by then implementing a level 2 definition, (iv) chose a rigorous disparity tolerance value ε such that the models must overcome a relatively high bar to satisfy a fairness definition, and (v) proposed implementing a form of statistical parity, which is a more extreme form of bias correction that can override patterns in the data arising from decades of structural bias. The simultaneous implementation of all techniques may suggests that we have created the most debiased model to-date relative to the reviewed literature, insofar as much as debias is defined as the lack of structural bias discussed in Sect. 5.3.

From a methods perspective, we make fairness and model-performance-related contributions. From a fairness perspective, we propose a novel implementation of maximizing performance under fairness, adopted from [57] (Eqs. 12–13, Fig. 5). From a performance perspective, we develop state-of-the-art models that surpass existing forecasting instruments. This success stems from training on a dataset that is both geographically and temporally broader than those used in prior studies (Table 2). Since this dataset was not originally intended for forecasting, we applied a novel and extensive combination of preprocessing techniques to make it suitable (Table 5), including a set of predictors informed by the criminology literature that can boost the performance of similar models (Table 6). This preprocessing step was relatively more involved than past research; yet, the use of this difficult dataset allows our models to be integrated relatively easily into the criminal justice system (Fig. 1). Thus, we demonstrate the preprocessing required to adapt such data for forecasting and the methodology for building state-of-the-art models.

From a deployment perspective, we provide novel recommendations that bridge the gap between machine learning, fairness research, and criminal justice. Specifically, we suggest how machine learning can address the structural biases of the criminal justice system (see Sect. 5.3); we propose a RCT to measure real-world impact (see Sect. 5.4); and we propose safeguards to limit the harm of false positives when the model is used in practice (see Sect. 5.1), among other measures. These recommendations offer concrete examples on how to render engineering terms and concepts intelligible to the community where the tool will ultimately be deployed. Finally, our study can serve as a blueprint for integrating machine learning into policing in a way that both reduce crime and addresses systemic inequities in criminal justice data—a blueprint that can be followed by others within our field.

## Supplementary Information

Below is the link to the electronic supplementary material.

Supplementary file1 (DOCX 162 KB)

## Data Availability

The conviction data used in this study cannot be shared due to ethical and legal restrictions.
