# Supplementary material for: A fairness scale for real-time recidivism forecasts using a national database of convicted offenders
Source: Neural Comput Appl. 2025 Aug 1;37(26):21607–57. doi: 10.1007/s00521-025-11478-x (PMC12401775; doi:10.1007/s00521-025-11478-x)
Supplement: Supplementary file 1 — Supplementary file1 (DOCX 162 KB) [file 521_2025_11478_MOESM1_ESM.docx]

**SUPPLEMENTAL MATERIALS**

**A Fairness Scale for Real-Time Recidivism Forecasts Using a National Database of Convicted Offenders**

Jacob Verrey^1*^, Peter Neyroud^1^, Lawrence Sherman^1, 2^, Barak Ariel ^1, 3^

^1^ Institute of Criminology, University of Cambridge, Sidgwick Ave, Cambridge CB3 9DA, UK

^2^ Benchmark Cambridge Ltd., Rectory Lane, Somersham, PE28 3EL, UK

^3^ Institute of Criminology, The Hebrew University of Jerusalem Mt. Scopus, 9190501, Jerusalem, Israel

* Corresponding author. *Email:* jjv31@cam.ac.uk **(**J. Verrey)

# Table of Contents

Contents

[Table of Contents 2](#_Toc200032909)

[1 | Supplemental Introductory Materials 3](#_Toc200032910)

[1.1 | Estimating Annual Cost of Crime for Convicted Offenders Who Recidivate 3](#_Toc200032911)

[2 | Supplemental Methods and Results 5](#_Toc200032912)

[2.1 | Preprocessing 5](#_Toc200032913)

[2.2 | Illustrative Confusion Matrices 24](#_Toc200032914)

[2.3 | Full Fairness Results, Grouped by Race 25](#_Toc200032915)

[3 | Supplemental References 28](#_Toc200032916)

# 1 | Supplemental Introductory Materials

## 1.1 | Estimating Annual Cost of Crime for Convicted Offenders Who Recidivate

There were 1.01 million convicted offenders for the year ending in March 2022 [1], with previous years oscillating between 0.905 and 1.16 million [2, 3]. As many as 83% of those convicted offenders will recidivate [4], or commit another crime, with more moderate estimates placing the figure between 30% and 60% [5, 6]. To estimate the number of crimes these individuals will commit, we use the one-year reconviction rate for England and Wales of 48%, taken from the year ending in March 2016 [6]. Taken together, these statistics suggest that convicted offenders will commit between 434,400 and 556,800 crimes one year after release, using conviction numbers from 2020 and 2021 respectively [2, 3].

The average unit cost of crime varies dramatically, ranging from £3,217,740 for a homicide to £550 for a cybercrime incident [7]. It is difficult to estimate the cost without more information on the types of crimes these convicted offenders commit. To provide some sort of estimation, however, we calculate that the median cost of crime is £6,520. Using the median cost of crime, these 434,400 and 556,800 crimes translated to a financial loss of £2,836,632,000 and £3,630,336,000 respectively. Moreover, this financial loss represents a yearly expense, as a different batch of convicted offenders are re-released every year.

Finally, this annual financial loss is both a crude and highly conservative estimate. First, we use the one-year reconviction rate; any crimes that were committed after one year, or any crimes that were not detected *or* not successfully prosecuted are omitted. Second, we use the median unit cost of crime, yet the average cost of crime is far higher due to the disproportionate costs of more serious crimes [7]. To correct these shortcomings, it is possible to use prosecution statistics, the ‘dark figure’ of nonreported crimes, and a crime portfolio of convicted offenders to reach a more accurate estimation [8]. Yet, our estimation should be sufficient for illustrating the potential financial benefits of crime prevention.

# 2 | Supplemental Methods and Results

## 2.1 | Preprocessing

Each row in the initial dataset represented a convicted offender at a particular court date, and it contains information such as (i) the offense details, (ii) disposition(s) resulting from the offense, and (iii) limited demographic details about the offender, such as age, gender, race, and location proxies. In its current state, the dataset could not be effectively used to construct machine learning models. Thus, we undertook three preprocessing operations to render it useful: (i) feature engineering and removal and (ii) data exclusions, and (iii) data encoding. The contents of our finished, preprocessed dataset appear in Table S1 below.

| **Table S1**. Descriptive statistics of categorical features and labels in PNC dataset. These descriptives were taken after performing all preprocessing operations *except* one-hot encoding. This is because one-hot encoding merely changes the structure of the database, not the contents; it tends to make the dataset far less legible to humans, far more legible to machine learning. All values are rounded to four significant figures.  Within the feature column, **Bold** text indicates a category, ***bold and italics*** text indicates a feature, and plain text indicates a unique value. | | | |
| --- | --- | --- | --- |
| **Feature** | **Count** | **Percentage** |  |
| **Demographics** |  |  |  |
| ***Sex*** |  |  |  |
| Female | 53070 | 15.3078 |  |
| Male | 293412 | 84.6336 |  |
| Unknown | 203 | 0.0586 |  |
| ***Race*** |  |  |  |
| Asian | 10639 | 3.0688 |  |
| Black | 20974 | 6.0499 |  |
| Other | 7032 | 2.0284 |  |
| White | 308040 | 88.853 |  |
| ***Is Minor*** |  |  |  |
| False | 278523 | 80.3389 |  |
| True | 68162 | 19.6611 |  |
| **Current Disposition(s)** |  |  |  |
| ***Category of First Disposition*** |  |  |  |
| Absolute Discharge | 1612 | 0.465 |  |
| Caution | 55423 | 15.9866 |  |
| Community Penalty | 72185 | 20.8215 |  |
| Conditional Disposition | 37294 | 10.7573 |  |
| Fine | 83979 | 24.2234 |  |
| Fully Suspended | 1470 | 0.424 |  |
| Immediate Custody | 66916 | 19.3017 |  |
| Other | 22546 | 6.5033 |  |
| PND | 5260 | 1.5172 |  |
| ***Category of Second Disposition*** |  |  |  |
| Absolute Discharge | 6 | 0.0017 |  |
| Caution | 55423 | 15.9866 |  |
| Community Penalty | 9828 | 2.8349 |  |
| Conditional Disposition | 248 | 0.0715 |  |
| Fine | 844 | 0.2434 |  |
| Fully Suspended | 37 | 0.0107 |  |
| Immediate Custody | 851 | 0.2455 |  |
| Other | 151215 | 43.6174 |  |
| PND | 5260 | 1.5172 |  |
| Not Applicable (no second disposition) | 122973 | 35.4711 |  |
| ***Category of Third Disposition*** |  |  |  |
| Absolute Discharge | 1 | 0.0003 |  |
| Caution | 55423 | 15.9866 |  |
| Community Penalty | 889 | 0.2564 |  |
| Conditional Disposition | 6 | 0.0017 |  |
| Fine | 36 | 0.0104 |  |
| Fully Suspended | 9 | 0.0026 |  |
| Immediate Custody | 54 | 0.0156 |  |
| Other | 61989 | 17.8805 |  |
| PND | 5260 | 1.5172 |  |
| Not Applicable (no third disposition) | 223018 | 64.3287 |  |
| ***Category of Fourth Disposition*** |  |  |  |
| Caution | 55423 | 15.9866 |  |
| Community Penalty | 143 | 0.0412 |  |
| Conditional Disposition | 4 | 0.0012 |  |
| Fine | 3 | 0.0009 |  |
| Fully Suspended | 2 | 0.0006 |  |
| Immediate Custody | 16 | 0.0046 |  |
| Other | 11695 | 3.3734 |  |
| PND | 5260 | 1.5172 |  |
| Not Applicable (no third disposition) | 274139 | 79.0744 |  |
| **Current Offense** |  |  |  |
| ***Offense Class*** |  |  |  |
| Breach offenses | 24765 | 7.1434 |  |
| Burglary, robbery, theft | 108382 | 31.2624 |  |
| Criminal damage | 10224 | 2.9491 |  |
| Drug offense | 33233 | 9.5859 |  |
| Fraud and forgery | 8653 | 2.4959 |  |
| Offenses outside England and Wales | 30971 | 8.9335 |  |
| Other indictable offenses | 21029 | 6.0657 |  |
| Sexual offense | 1631 | 0.4705 |  |
| Summary offenses excluding motoring | 87905 | 25.3559 |  |
| Unknown | 2 | 0.0006 |  |
| Violent offenses against persons | 19890 | 5.7372 |  |
| ***Offense Involved Minor*** |  |  |  |
| False | 345060 | 99.5313 |  |
| True | 1625 | 0.4687 |  |
| ***Offense Involved Weapon*** |  |  |  |
| Not applicable | 339660 | 97.9737 |  |
| Chemicals | 259 | 0.0747 |  |
| Explosives | 58 | 0.0167 |  |
| Firearm | 988 | 0.285 |  |
| Knife | 5691 | 1.6415 |  |
| Other | 29 | 0.0084 |  |
| **Criminal History** |  |  |  |
| ***Has Targeted Minors*** |  |  |  |
| False | 335252 | 96.7022 |  |
| True | 11433 | 3.2978 |  |
| ***Has been involved with weapon*** |  |  |  |
| False | 284213 | 81.9802 |  |
| True | 62472 | 18.0198 |  |
| **Criminological Features** |  |  |  |
| ***Substance Abuse Risk*** |  |  |  |
| False | 228685 | 65.9633 |  |
| True | 118000 | 34.0367 |  |
| ***Age-Crime Risk Level*** |  |  |  |
| Low | 78,349 | 22.5995 |  |
| Medium | 83,660 | 24.1314 |  |
| High | 184,676 | 53.2691 |  |
| ***Ever Received Custodial Sentence*** |  |  |  |
| False | 279704 | 80.6796 |  |
| True | 66981 | 19.3204 |  |
| ***Ever Been Given Fine*** |  |  |  |
| False | 261992 | 75.5706 |  |
| True | 84693 | 24.4294 |  |

| **Table S2**. Descriptive statistics of continuous features in PNC dataset, obtained from the 346,685 disposition records. Most of the features listed below had values that were prominently zero. Thus, all zero values were excluded to meaningfully display these summary statistics. The number of values who are not missing-values for a given feature is indicated by the (n) value.  Within the feature column, **Bold** text indicates a category ***bold and italics*** indicates a subcategory, and plain text indicates a feature name. | | | | | | | |
| --- | --- | --- | --- | --- | --- | --- | --- |
| **Feature (*n*)** | ***M*** | ***SD*** | ***Median*** | ***25^th^/75^th^ Percentile*** | ***Mode*** | ***Min*** | ***Max*** |
| **Demographics** |  |  |  |  |  |  |  |
| Age at Court Sentencing (346,685) | 25.4800 | 9.5673 | 23 | 18/31 | 19 | 10 | 88 |
| **Current Disposition(s)** |  |  |  |  |  |  |  |
| Total Custodial Sentence in Days (66,423) | 1433.493 | 20120.35 | 120 | 60/360 | 90 | 1 | 368191 |
| Total Fine (84,493) | 156.7671 | 5184.459 | 60 | 50/100 | 50 | 1 | 1000000 |
| **Criminal History** |  |  |  |  |  |  |  |
| Total Breach Offenses over Life (134,889) | 3.4457 | 3.2862 | 2 | 1/4 | 1 | 1 | 53 |
| Total Violent Offences over Life (111,472) | 2.3037 | 2.1455 | 2 | 1/3 | 1 | 1 | 46 |
| Total Fraud or Forgery over Life (71,418) | 3.3920 | 4.8533 | 2 | 1/4 | 1 | 1 | 105 |
| Total Offenses Outside of England and Wales over Life (34,043) | 24.8661 | 27.5866 | 15 | 5/35 | 1 | 1 | 214 |
| Total Drug Offenses over Life (123,045) | 2.6008 | 2.7541 | 2 | 1/3 | 1 | 1 | 98 |
| Total Unknown Offenses over Life (23,423) | 2.6903 | 3.3744 | 1 | 1/3 | 1 | 1 | 40 |
| Total Burglary, Robbery, or Theft Offenses over Life (233,418) | 15.0985 | 20.6444 | 7 | 3/20 | 1 | 1 | 324 |
| Total Other Offenses over Life (162,333) | 4.5882 | 6.8750 | 3 | 1/6 | 1 | 1 | 205 |
| Total Criminal Damages over Life (109,153) | 2.7957 | 5.4384 | 2 | 1/3 | 1 | 1 | 182 |
| Total Sex Offenses over Life (9,467) | 1.7126 | 1.6895 | 1 | 1/2 | 1 | 1 | 32 |
| **Criminological Features** |  |  |  |  |  |  |  |
| ***Criminal Careers*** |  |  |  |  |  |  |  |
| Age of First Disposition (346,685) | 17.0693 | 6.7555 | 16 | 13/18 | 14 | 10 | 88 |
| Court Appearance Count over Life (305,429) | 12.33 | 21.114 | 8 | 3/16 | 1 | 1 | 599 |
| Offense Count over Life (305,429) | 25.6421 | 40.8047 | 14 | 5/34 | 1 | 1 | 900 |
| Years since Last Conviction (305,429) | 1.2445 | 2.2767 | 0.5479 | 0.2110 / 1.3753 | 0.0192 | 0.0027 | 48.8055 |
| Diversity index (305,429) | 0.5198 | 0.239 | 0.5926 | 0.4444 / 0.6947 | 0 | 0 | 0.876033 |
| ***Reintegrative Shaming*** |  |  |  |  |  |  |  |
| Total Custodial Sentence over Life (157,433) | 2268.11 | 7587.442 | 1090 | 360/2750 | 180 | 1 | 1100820 |
| Total Fine over Life (215,965) | 508.9527 | 9283.732 | 225 | 100/480 | 50 | 1 | 2725525 |
| ***Criminogenic Location*** |  |  |  |  |  |  |  |
| Total Offenses per 1000 people (310,701) | 63.6988 | 11.8193 | 62.7 | 53.1/74.6 | 84 | 38.1 | 84 |
| Total Drug Offenses per 1000 people (310,701) | 2.7321 | 1.0181 | 2.4 | 2.1/2.9 | 2.4 | 1.6 | 5.5 |

**2.1.1 | Feature Engineering.** To render the dataset legible to machine learning, as well as to boost the predictive validity of our ultimate machine learning model, feature engineering was undertaken. Feature engineering involves the addition of new features (columns) in the PNC dataset. This often involves injecting our domain expertise into the model, highlighting features known to predict future re-offending such as age of first disposition. This also involves removing irrelevant features from the model, such as a person’s name. A narrative and detailed justification behind each feature engineering step appears below, grouped by intention.

***2.1.1.1 | Understanding the Convicted Offender’s Offense.*** The dataset contained 2 features that summarized the offender’s offense: a Home Office Offense Code, and an Offense Class. Specifically, the offense code feature contains five-digit numeric codes. They are semi-arbitrary and hyper-specific; they are so specific that the code often changes when the minute details of an offense change, and they do not change in a predictable manner. To illustrate the hyper-specificity and arbitrariness of these codes, the offense code for violence against a person would be 00505 if chloroform was involved, 00506 if the victim was maimed via an explosion, and 00840 if religiously aggravated, among many other codes. Our dataset contained 657 unique codes, and it is difficult for a machine learning model to learn the relationship between these codes if they do not exhibit a meaningful semantic relationship.

Fortunately, the offense class feature summarizes these unique codes by disclosing the specific offense category that the code belonged to. For example, if an offense code represented a violent offense, it would belong to the offense class “Violent Offenses against Persons”, whereas a similar logic would hold to offense codes that represented sex offenses, criminal-damage-related offenses, etc. There were 11 of these codes, and they were sufficient representations of the offender’s offense. Thus, the hyper-specific offense code feature was dropped, whereas the more general offense class feature remained.

Before dropping the hyper-specific offense code feature, however, we used them to construct additional features that may help a machine learning model predict recidivism. First, we created a Boolean feature involved minor to indicate whether the offense involved the offender targeting a minor under 18 years old. Second, we created a categorical feature involved weapon that indicated the weapon involved in a particular offense. If no weapon was present, the feature was assigned to ‘none’, whereas if a weapon was present, it would contain the corresponding weapon. Finally, we used the offense code feature to implement our definition of violent recidivism that was discussed in the main text --- the hybrid definition that draws from both [9, 10]. This was to construct the label, as discussed in the next subsection; it was not used to construct a predictive feature.

***2.1.1.2 | Flagging Recidivism (Labels).*** To predict whether a convicted offender will recidivate, we needed a sort of ‘answer key’ that our machine learning model could use to determine if a convicted offender actually did recidivate. To construct the answer key, we constructed two labels: a Boolean feature that flagged a convicted offender as recidivating if he/she was reconvicted within a three-year period, whereas the other label did the same except for only those reconvictions that were violent offenses. Violent offenses were defined by manually grouping offenses via the Home Office offense code feature in a way that implemented the hybrid definition of violence discussed in the main text [9, 10]. Moreover, we were able to link individual offenders to specific offense codes via a person ID feature. Ultimately, these Boolean labels were named 3 year all recidivism and 3 year violent recidivism.

These two labels – violent and general recidivism – appeared in our dataset simultaneously. For this reason, it is crucial to stress that only one label was used to train the models. In other words, when we trained machine learning models to forecast general recidivism, we ensured that the violent recidivism label was completely removed from the dataset and vice versa. This removal ensures that predictions for one type of recidivism had no bearing on predictions for the other, thereby ensuring these models are truly independent of each other and thereby combatting data leakage.

***2.1.1.3 | Understanding Age.*** Next, the dataset contained the convicted offender’s age at the time of PNC download. Thus, we used their age at PNC download – in tandem with their court date – to create a feature called age at time of disposition, which recorded their age at the time they received their disposition. This led us to dropping the original age feature. We also created a Boolean feature that flagged whether they were a minor at the time of disposition because this would affect sentencing, which would be set to True if the offender was 17 or younger at the time of his/her disposition.

***2.1.1.4 | Understanding the Convicted Offender’s Disposition(s*).** Each convicted offender could have received up to 4 court dispositions, and there were 5 features that summarized each disposition: (i) the type of disposition the convicted offender received (ii) the fine associated with the disposition (if any), (iii) the days in custody that the convicted offender was sentenced to, associated with that specific disposition (if any), (iv) the rank of the disposition, and (v) any annotations or notes the practitioner wishes to add concerning the disposition. The disposition rank feature was meaningless because the rank of the dispositions were already given in the name. For example, if the convicted offender received three dispositions, then they would be stored under the as DisCat1, DispCat2, DispCat3 columns in our dataset based on rank, respectively. Moreover, the practitioner’s notes and annotations were also meaningless because they were primarily missing values, and, of the few non-missing values, the annotations were highly idiosyncratic, following no standardized pattern. Thus, both types of features – (iii) the custom annotation feature and (iv) the rank of the dispositions – were dropped for each of the 4 potential dispositions, resulting in 8 total exclusions: DispText1, DispText2, DispText3, DispText4, DispRank1, DispRank2, DispRank3, and DispRank4.

Next, each individual disposition could have its own fine and/or custodial sentence associated with it. However, it would be more effective to summarize the *total* custodial sentence and *total* fine because this is the actual punishment that the convicted offender received. Thus, we constructed two features – total custodial sentence in days and total fine – that summed the individual custodial sentence and total fine from each of the four dispositions. This rendered the original features they drew from redundant, hence, they were dropped, resulting in 8 exclusions: DispDays1, DispDays2, DispDays3, DispDays4, DispAmt1, DispAmt2, DispAmt3, and DispAmt4.

Finally, there are 4 remaining features, with each feature summarizing the disposition(s) that each convicted offender received: DispCat1, DispCat2, DispCat3, and DispCat4. We did not modify any of these features because individual disposition summaries are sufficiently meaningful.

***2.1.1.5 | Constructing Criminal History using Current Offense, Disposition(s), and Age.*** We also constructed criminal history from the PNC dataset to incorporate it into recidivism predictions. Specifically, we constructed 11 counter features that incremented each time a convicted offender committed an offense. These counter features were Total Breach Offenses over Life, Total Burglary, Robbery, or Theft Offenses over Life, Total Criminal Damages over Life, Total Drug Offenses over Life, Total Fraud and Forgery over Life, Total Offences Outside of England and Wales over Life, Total Other Offenses over Life, Total Sex Offenses over Life, Total Summary Offenses over Life, Total Unknown Offenses over Life, Total Violent Offenses Against Person over Life. Much like the label, we used the person ID feature to link each convicted offender to his/her past convictions.

Finally, we constructed a series of Boolean features. We created ever involved with weapon and ever targeted minor features that recorded if, over the course of the offender’s lifetime, he/she has been convicted of an offense that involved weapons or minors respectively.

***2.1.1.6 | Removing Other Confounding Features.*** We removed the court date feature because it is difficult for a machine learning algorithm to parse a date-time value. Moreover, we removed the three features related to location. Specifically, we dropped Address Postcode because it was primarily missing values. Process Force Code and Offense Force Code contain the name of the agency that processed a criminal offender and the name of the agency which initially responded to the offense, respectively. The force listed in both features is the same in roughly 95% of cases – i.e., the same force that responded to the offender also processed him/her – and both could be used as meaningful proxy for location.

However, there were 73 unique processing forces, and for a machine learning model to meaningfully handle these categorical strings, we would need to one-hot encode them [11]. This would have added 73 new columns to our dataset, which would have seriously slowed down the training process. Thus, we conducted a pilot test and found that removing this feature did not majorly affect model performance while, at the same time, massively shortened training time. Thus, we elected to remove these location proxies from the final dataset. Finally, we removed the PersonID feature because we did not want a person’s name to influence a machine learning model’s forecast, and we removed the isPrimaryOffense feature because our dataset only contained primary offenses after the data exclusion process, hence, this feature simply became a non-meaningful constant (see data exclusions).

***2.1.1.7 | Criminological Features.*** Next, we reviewed the criminological literature for insights that can be used to construct features indicative of future reoffending. When reviewing the literature, we were greatly limited by the sparsity of our PNC dataset: it does not contain clinical features or exhibit detailed demographic information, and access to these features would have allowed us to leverage more criminological theories to derive criminological predictors. For example, the general theory of crime posits that low self-control is a robust predictor of future criminality [12]. Therefore, if we had access to psychological assessments that measured self-control, we could have engineered features based on the general theory of crime, thereby bolstering the performance of our models.

Therefore, the sparsity of the PNC greatly restricted the number of criminological predictors we could derive we could leverage. Indeed, we were mostly limited to just criminological theories that made use of criminal history and demographics.

*2.1.1.7.1 | Criminal Careers.* First, we used the criminal careers paradigm [13] to construct 5 features that measure the presence of a criminal career. In other words, the criminal career paradigm posits that there is a subpopulation that routinely engages in reoffending. Numerous features can be engineered to detect this criminal subpopulation, and if it they can be detected, reoffending can be better predicted.

The paradigm proposes several feature categories that can be used to measure the presence of a criminal career: (i) onset, (ii) offending frequency, (iii) desistence, and (iv) specialization. First, the earlier in life that an individual offends, the more likely he/she is to offend again. Thus, the criminal onset phenomenon is captured via the age of first disposition feature. Second, offending frequency is measured via the court appearance count and offense count features because greater offending frequency is indicative of future reoffending. Third, offenders can desist from future reoffending where, the longer they desist, the less likely they are to offend again in the future. Thus, desistance is measured via the years since last disposition feature. Finally, crime specialization can be used to detect criminal careers. Thus, the diversity index was used to measure the extent of crime specialization [14]. The diversity index was chosen over similar indices because it does not require similar offenses to be sequential, thereby giving it greater flexibility in measuring specialization. The equation appears below and is provided by [14]:

(S1) $D=1-\sum_{m=1}^{M} p_{m}^{2}$

*p* is the proportion of offences in crime category *m*. The diversity index *D* ranges from 0, which indicates complete specialization, to a maximum value defined by (*M* -1)/*M*, where *M* represents the total number of crime categories *m*. There are eleven categories total; thus, the maximum diversity index *D* is 0.91. Finally, it is crucial to note that there are other aspects of the criminal careers paradigm that are not captured above – such as escalation or de-escalation; however, these were the insights that could be most easily derived given the sparsity of the PNC.

*2.1.1.7.2 | Reintegrative Shaming.* Reintegrative shaming argues that individuals may experience stigmatization after they are exposed to the judicial system [15]. This may make individuals more likely to be treated as an outcast and thereby experience resentment, and this resentment may ultimately make the individuals more likely to commit another offense. While any exposure to the judicial system may cause stigmatization, it may be particularly acute if the offender during incarcerated or if they receive a particularly negative court disposition. To measure stigmatization, we therefore construct the ever received custodial sentence and ever received fine features to record whether the convicted offender was ever sent to prison or received a fine, respectively. After constructing these counters, we created two additional features - total custodial sentence over life and total fine over life – that summed together the total custodial sentence and total fine that the convicted offender has received over his/her lifetime, as the larger these two features, the more likely they are to experience stigmatization and ultimately recidivate.

*2.1.1.7.3 | Age Crime Curve.* The age crime curve is a remarkably consistent finding that suggests that certain ages are far more likely to offend than others. For example, [16] demonstrates that 15-24 year olds were at the highest risk of offending, followed by 25-34 year olds, followed by the all other age groups. The remarkable observation is that this finding held across both time and geographies, holding true for both US arrests in the 1980s and ocnvictions in Old Bailey, London between 1800-1900. Indeed, the age crime curve is hailed as one of the most remarkable and consistent findings within criminology [12].

Thus, we baked the age-crime curve into our model. Specifically, we constructed the feature age crime risk level that flagged an offender as higher risk if he/she was between 15-24, medium risk if he/she was between 25-34, and low risk for all other ages, following the curve proposed by [16]. If an offender received a custodial sentence, then the age that he is expected to be released from prison was used to estimate risk instead of his/her age in court. For example, if an offender was 20 years old in court and received a 10-year prison sentence, he would be labelled as medium risk because he may not be released from prison until he is 30 years old.

*2.1.1.7.4 | Criminogenic Location.* Routine Activities Theory suggests that certain places can be criminogenic such that some locations are more conducive to crime than others [17]. For example, crime is more likely to occur at an unguarded jewelry store than at a highly guarded equivalent. To measure how criminogenic a certain place is, one typically needs access to the specific postal code of the crime, and therein lies the problem: this geographic granularity is not available in the PNC. In other words, the PNC sometimes listed the convicted offender’s address via Address Postcode; however, this was mostly missing values and therefore cannot be used (see §2.1.1.6.7). Instead, the Process Force Code feature appeared to be the most reliable, granular measure of geography because it stated the law enforcement agency that processed the convicted offender. Despite being the most reliable, granular measurement available, it was not unideal: most UK law enforcement agencies large geographic areas, making this measurement a coarse proxy for location.

To gain some understanding of how criminogenic a place is, the Process Force Code was used as a proxy for where that crime occurred, and 2 derivative features were constructed from that proxy. First, crime data were obtained via the Office for National Statistics PFA Tables [18]. The PFA tables are unique in that they are one of the few datasets that aggregate crime by the jurisdiction that each law enforcement agency protects, meaning they map nicely onto the Process Force Code feature. Using the PFA tables, the features the total offenses per 1000 people and total drug offenses per 1000 people were imported into the PNC. The former was extracted to serve as a loose proxy for how criminogenic a location is, whereas the latter was extracted because substance abuse independently predicts recidivism [19]; thus, its criminogenic equivalent – drug offenses – should meaningfully boost the performance of our models.

*2.1.1.7.5 | Other Criminological Risk Factors.* Factor analysis consistently suggests that substance abuse can be a powerful predictor of recidivism [19]. Thus, we constructed the Boolean substance abuse risk where, if an offender was ever convicted of drug possession, he/she would be flagged as a risk.

**2.1.2 | Data Exclusions.** We remedied three existing problems via data exclusions. First, we overcame the issue of simultaneous convictions by excluding non-primary offenses (*n*=774,446). Second, we combat concept drift by setting a minimum court date requirement, and we ensured a valid measurement window by setting a maximum court date requirement, resulting in 520,535 date-related exclusions. Finally, we discarded individuals with an anomalous age, resulted in a further 109 exclusions. This resulted in a final dataset of 346,685 convicted offenders at a particular court date between January 1, 2000, and February 3, 2006.

*2.1.2.1 | Overcoming the Issue of Simultaneous Convictions: Dropping Non-Primary Offenses.* First, each row in the dataset should represent one offender at a particular court date. Due to an idiosyncrasy with the database’s architecture, however, this ideal was not accomplished. In other words, individuals are often convicted of *multiple* offenses at once. When this occurred, their most serious offense would be marked as the primary offense, whereas any less serious offense(s) would be marked as secondary; these secondary offenses would receive their own unique row on the dataset. Non-primary offenses appear in our dataset in the same manner as primary offenses, with the sole difference being the value of the isPrimaryOffense Boolean. An Illustration of the dataset appears in Table S3

| **Table S3.** Dataset Illustration. Illustration indicates the issue of simultaneous convictions, where one offender may be convicted of multiple offenses on the same court date. When this occurs, isPrimary is set to True, indicating the most serious offense. In this example, the offender was convicted of three offenses on the same court date: two criminal damages, and one drug, with the drug offense being the most serious, as indicated by TotalFine. | | | | | | | | | | | | | | | | | |
| --- | --- | --- | --- | --- | --- | --- | --- | --- | --- | --- | --- | --- | --- | --- | --- | --- | --- |
| **Demographics** | | | |  | **Offense** | | | |  | **Disposition** | | | |  | **Criminal History** | | |
| ***Person ID*** | ***Sex*** | ***…*** | ***Age*** |  | ***Court Date*** | ***Offense Class*** | ***…*** | ***isPrimary*** |  | ***DispCat1*** | ***…*** | ***DispCat4*** | ***TotalFine*** |  | ***Past Criminal Damage*** | ***…*** | ***Past Breach*** |
| 128 | Male | … | 24 |  | 1/4/ 2000 | Drug Offense | … | True |  | Fine | … | Other | 1000.00 |  | 0 | … | 5 |
| 128 | Male | … | 24 |  | 1/4/ 2000 | Criminal Damages | … | False |  | Fine | … | None | 360.00 |  | 0 | … | 5 |
| 128 | Male | … | 24 |  | 1/4/ 2000 | Criminal Damages | … | False |  | Fine | … | None | 30.50 |  | 0 | … | 5 |

The issue with simultaneous convictions – that is one primary offense, at least one secondary offense – is that this would cause our model to output multiple recidivism predictions for the same convicted offender at the exact same court date. For example, if an offender was convicted of three offenses during one court date, he would receive three predictions: one recidivism prediction per offense. This would create potentially contradicting information for criminal justice practitioners. Thus, we elected to drop non-primary offenses (*n*=774,446) such that the convicted offender only receives a recidivism prediction for the most serious offense he/she was convicted of on that particular date. This solves the issue of simultaneous convictions and their resulting simultaneous predictions.

This approach could have resulted in non-primary offenses being excluded from the offender’s recidivism prediction; yet, our criminal history features prevent that. In other words, we constructed several features that measure each convicted offender’s criminal history (see Feature Engineering). This means that, if an offender is facing simultaneous convictions, only the most serious offense will be reflected in the model’s predictions in that instance; however, the next time a convicted offender is convicted, all the non-primary offenses of the prior case will appear. For example, imagine an offender is convicted on January 1, 2000, for 5 offenses: one attempted murder – the most serious – and 4 theft offenses. On January 1, the model will only use the most serious offense - attempted murder - to predict recidivism. However, when the offender appears in court again – say, May 31, 2004 - the model will know he was convicted of 5 prior offenses - one attempted murder, 4 theft – due to the criminal history feature, as well as the new conviction, thereby factoring all of these offenses into its prediction.

This approach is not perfect; yet, generating predictions from the most serious offense – over the least serious ones - should serve as a relatively strong foundation for a recidivism forecast. Moreover, our approach solves the issue of simultaneous forecasts that stem from the inclusion of non-primary offenses.

*2.1.2.2 | Combatting Concept Drift & Ensuring Valid Measurement Period: Date-Related-Exclusions.* We also set a minimum and maximum court date. First, the dataset appears to contain court records that were added retrospectively; it contained a case as early as 1900, for example. This is problematic because of concept drift, a well-documented machine learning phenomenon in which data changes over time [20]. In other words, a model trained on data from the early-to-mid 20^th^ century may not be able to accurately forecast recidivism for data obtained in the 21^st^ century because recidivism predictors could have changed drastically during that timeframe. To combat concept drift, we therefore required that each case occur on January 1, 2000, or later, resulting in 365,877 exclusions. Second, our dataset was downloaded on February 3, 2011. When training the model, we wanted to ensure the convicted offenders had enough time to recidivate in order to be detected by our label; thus, we set the maximum date to February 3, 2006. This resulted in 154,658 exclusions, bringing the total date-related exclusions to 520,535.

*2.1.2.3 | Correcting Anomalous Age.* Finally, we excluded individuals who an infeasibly old age. Specifically, there were 109 individuals who were reported to be 111 years old. This was highly anomalous: 111-year-olds are considered supercentenarians that are extremely rare in Britain. Moreover, the second oldest person in our dataset was 99 years old (*n*=1), creating a ten-year year age gap in which there were no individuals aged between 100 and 110, inclusive. Therefore, we assumed that ‘111’ was a missing value, and thus, these 191 individuals were dropped. In criminal justice, it is especially important to drop individuals whose age is unknown because age tends to be one of the greatest predictors of crime [21]. Thus, incorporating individuals with a noticeably incorrect age could confound a model’s ability to learn patterns within a highly predictive feature. This resulted in a total of 346,685 offenses. Full details of age appear in Table S4 below.

| **Table S4. Abridged Age Frequency Table.** Table is intended to showcase the anomaly between the second-oldest age (99) and the anomalous age (111). | |
| --- | --- |
| **Age** | **Frequency** |
| 10 | 1 |
| 11 | 2 |
| 14 | 10 |
| 15 | 1 |
| 18 | 2 |
| 19 | 3 |
| 20 | 489 |
| 21 | 1,882 |
| 22 | 4,469 |
| 23 | 7,720 |
| 24 | 12,329 |
| … | … |
| 97 | 1 |
| 98 | 2 |
| 99 | 1 |
| 111 | 109 |

**2.1.3 | Data Encoding.** Machine learning algorithms cannot nicely handle nominal categorical features with string values, so all such features were one-hot encoded. One-hot encoding involved transforming categorical features into binaries; it changes the *structure* of the database, not its contents. For example, the categorical feature Gender feature may have three string values: male, female, and unknown. However, after one-hot encoding, the original Gender feature would be deleted, and three new features would take its place: Gender Male, Gender Female, and Gender Unknown which would take a True or False Boolean value. One-hot encoding is disadvantageous because it may massively expand the columns in the database, potentially increasing training time; yet, it follows best practices on rendering categorical features intelligible to machine learning algorithms [11], potentially boosting performance.

## 2.2 | Illustrative Confusion Matrices

A confusion matrix for our best performing general and violent recidivism models appears below, which are the gradient boosting machine and random forest respectively. These are the models that appear in §4.1 in the main text; hence, no fairness definitions are implemented. All values are taken from fold #0.

| **Table S5**. Confusion matrix for the best performing violent recidivism forecasting model, the random forest. | | | |
| --- | --- | --- | --- |
|  |  | **Predicted** | |
|  |  | **Recidivism** | **No Recidivism** |
| **Actual** | **Recidivism** | 4,953(**TP**) | 15,063 (**FN**) |
|  | **No Recidivism** | 867 (**FP**) | 48,454(**TN**) |

| **Table S6**. Confusion matrix for the best performing general recidivism forecasting model, gradient boosting machine | | | |
| --- | --- | --- | --- |
|  |  | **Predicted** | |
|  |  | **Recidivism** | **No Recidivism** |
| **Actual** | **Recidivism** | 55,023 (**TP**) | 2,445 (**FN**) |
|  | **No Recidivism** | 6,842 (**FP**) | 5,027 **TN**) |

## 2.3 | Full Fairness Results, Grouped by Race

Full results appear on the next page via Table S7.

| **Table S7.** Full results of all fairness models, separated by race. Recall that, to satisfy a fairness definition, the statistical range of the corresponding metric must be less than one percentage point (0.01). If an implementation of fairness required equalizing a particular metric, the values of that metric are indicated through green shading. Standard deviations were omitted.  ***PR*** stands for positive rate, and its range measures the implementation of statistical parity. ***Error Bal*** stands for error balance, and its range measures the implementation of equal treatment. The statistical range of ***Recall*** measures the implementation for equal opportunity, whereas the equivalent for ***Precision*** is predictive parity. | | | | | | | | | | | | |
| --- | --- | --- | --- | --- | --- | --- | --- | --- | --- | --- | --- | --- |
| **Model** |  | **General Recidivism** | | | | |  | **Violent Recidivism** | | | | |
|  |  | ***PR*** | ***Error Bal*** | ***Recall*** | ***Precision*** | ***F_1_*** |  | ***PR*** | ***Error Bal*** | ***Recall*** | ***Precision*** | ***F_1_*** |
| **Baseline Model** |  |  |  |  |  |  |  |  |  |  |  |  |
| Asian |  | 0.8200 | 0.4713 | 0.9185 | 0.8410 | 0.8780 |  | 0.0301 | 43.9680 | 0.1057 | 0.8329 | 0.1870 |
| Black |  | 0.9148 | 0.3589 | 0.9611 | 0.8984 | 0.9287 |  | 0.0906 | 22.4076 | 0.2234 | 0.8645 | 0.3547 |
| Other |  | 0.4542 | 1.2853 | 0.7851 | 0.8217 | 0.8027 |  | 0.0261 | 25.3533 | 0.1808 | 0.8435 | 0.2967 |
| White |  | 0.9016 | 0.3423 | 0.9599 | 0.8911 | 0.9242 |  | 0.0888 | 16.4849 | 0.2611 | 0.8527 | 0.3994 |
| **Level 1: No Explicit Discrimination**  ***Fairness through Unawareness.*** |  |  |  |  |  |  |  |  |  |  |  |  |
| Asian |  | 0.8238 | 0.4496 | 0.9213 | 0.8397 | 0.8786 |  | 0.0340 | 24.9720 | 0.1061 | 0.7404 | 0.1855 |
| Black |  | 0.8949 | 0.5301 | 0.9477 | 0.9056 | 0.9262 |  | 0.0812 | 27.1199 | 0.2013 | 0.8703 | 0.3267 |
| Other |  | 0.5681 | 0.4123 | 0.8638 | 0.7229 | 0.7870 |  | 0.0304 | 12.9244 | 0.1806 | 0.7248 | 0.0471 |
| White |  | 0.9011 | 0.3463 | 0.9594 | 0.8912 | 0.9241 |  | 0.0885 | 16.6198 | 0.2606 | 0.8538 | 0.3992 |
| **Level 2: Equal Outcome**  ***Statistical Parity*** |  |  |  |  |  |  |  |  |  |  |  |  |
| Asian |  | 0.8698 | 0.2348 | 0.9517 | 0.8216 | 0.8818 |  | 0.3217 | 0.5086 | 0.6446 | 0.4770 | 0.5476 |
| Black |  | 0.8718 | 0.7859 | 0.9315 | 0.9138 | 0.9225 |  | 0.3183 | 1.3126 | 0.5955 | 0.6574 | 0.6246 |
| Other |  | 0.8637 | 0.0250 | 0.9790 | 0.5389 | 0.6950 |  | 0.3162 | 0.0928 | 0.8407 | 0.3258 | 0.4690 |
| White |  | 0.8728 | 0.5952 | 0.9405 | 0.9020 | 0.9208 |  | 0.3207 | 0.7678 | 0.6741 | 0.6107 | 0.6403 |
| **Level 2: Equal Performance**  ***Equal Opportunity*** |  |  |  |  |  |  |  |  |  |  |  |  |
| Asian |  | 0.8165 | 0.4954 | 0.9168 | 0.8433 | 0.8783 |  | 0.3943 | 0.3257 | 0.7154 | 0.4363 | 0.5395 |
| Black |  | 0.8553 | 1.0296 | 0.9187 | 0.9187 | 0.9185 |  | 0.4234 | 0.6247 | 0.7157 | 0.5974 | 0.6486 |
| Other |  | 0.6530 | 0.2001 | 0.9112 | 0.6654 | 0.7686 |  | 0.2142 | 0.3237 | 0.7139 | 0.4192 | 0.5222 |
| White |  | 0.8425 | 0.9546 | 0.9184 | 0.9125 | 0.9153 |  | 0.3547 | 0.6159 | 0.7117 | 0.5868 | 0.6403 |
| ***Treatment Equality*** |  |  |  |  |  |  |  |  |  |  |  |  |
| Asian |  | 0.8345 | 0.3864 | 0.9301 | 0.8368 | 0.8810 |  | 0.3154 | 0.7867 | 0.6212 | 0.4943 | 0.5332 |
| Black |  | 0.9112 | 0.3862 | 0.9589 | 0.8999 | 0.9285 |  | 0.4301 | 0.7963 | 0.7060 | 0.5970 | 0.6348 |
| Other |  | 0.5726 | 0.3887 | 0.8718 | 0.7238 | 0.7907 |  | 0.1673 | 0.7918 | 0.6196 | 0.4904 | 0.5276 |
| White |  | 0.8961 | 0.3841 | 0.9564 | 0.8934 | 0.9238 |  | 0.3591 | 0.7841 | 0.7033 | 0.5894 | 0.6287 |
| ***Predictive Parity*** |  |  |  |  |  |  |  |  |  |  |  |  |
| Asian |  | 0.7792 | 0.7487 | 0.8895 | 0.8572 | 0.8730 |  | 0.2386 | 1.0328 | 0.5365 | 0.5384 | 0.5359 |
| Black |  | 0.9891 | 0.0130 | 0.9980 | 0.8629 | 0.9255 |  | 0.5254 | 0.3278 | 0.7990 | 0.5419 | 0.6421 |
| Other |  | 0.4105 | 2.0934 | 0.7372 | 0.8539 | 0.7911 |  | 0.1218 | 1.0247 | 0.5388 | 0.5422 | 0.5398 |
| White |  | 0.9592 | 0.0722 | 0.9888 | 0.8629 | 0.9215 |  | 0.4277 | 0.3390 | 0.7864 | 0.5389 | 0.6369 |

# 3 | Supplemental References

1. Ministry of Justice (2022) Criminal Justice Statistics quarterly, England and Wales, year ending March 2022. Ministry of Justice, London, UK. https://assets.publishing.service.gov.uk/media/62fd0d26e90e0703e39e3746/criminal-justice-statistics-march-2022.pdf. Accessed 13 March 2024

2. Ministry of Justice (2020) Criminal Justice Statistics quarterly, England and Wales, April 2019 to March 2020. Ministry of Justice, London, UK. https://assets.publishing.service.gov.uk/media/5f3d44018fa8f5174ac78fa0/criminal-justice-statistics-quarterly-march-2020.pdf. Accessed 13 March 2024

3. Ministry of Justice (2021) Criminal Justice Statistics quarterly, England and Wales, year ending March 2021. Ministry of Justice, London, UK. https://assets.publishing.service.gov.uk/media/611d0635e90e070545560ce0/criminal-justice-statistics-march-2021.pdf. Accessed 13 March 2024

4. Alper M, Durose MR, Markman J (2018) 2018 update on prisoner recidivism: A 9-year follow-up period (2005-2014). US Department of Justice, Washington, D.C. https://bjs.ojp.gov/content/pub/pdf/18upr9yfup0514.pdf. Accessed 5 June 2025

5. Fazel S, Wolf A (2015) A Systematic Review of Criminal Recidivism Rates Worldwide: Current Difficulties and Recommendations for Best Practice. PLoS ONE 10:e0130390. https://doi.org/10.1371/journal.pone.0130390

6. Yukhnenko D, Sridhar S, Fazel S (2020) A systematic review of criminal recidivism rates worldwide: 3-year update. Wellcome Open Res 4:28. https://doi.org/10.12688/wellcomeopenres.14970.3

7. Heeks M, Reed S, Tafsiri M, Prince S (2018) The economic and social costs of crime, Second edition. Home Office, London, UK. https://assets.publishing.service.gov.uk/media/5b684f22e5274a14f45342c9/the-economic-and-social-costs-of-crime-horr99.pdf. Accessed 29 October 2023

8. Pina-Sánchez J, Buil-Gil D, Brunton-Smith I, Cernat A (2023) The Impact of Measurement Error in Regression Models Using Police Recorded Crime Rates. J Quant Criminol 39:975–1002. https://doi.org/10.1007/s10940-022-09557-6

9. Salo B, Laaksonen T, Santtila P (2019) Predictive Power of Dynamic (vs. Static) Risk Factors in the Finnish Risk and Needs Assessment Form. Criminal Justice and Behavior 46:939–960. https://doi.org/10.1177/0093854819848793

10. Tollenaar N, Van Der Heijden PGM (2013) Which Method Predicts Recidivism Best?: A Comparison of Statistical, Machine Learning and Data Mining Predictive Models. Journal of the Royal Statistical Society Series A: Statistics in Society 176:565–584. https://doi.org/10.1111/j.1467-985X.2012.01056.x

11. Zheng A, Casari A (2018) Feature engineering for machine learning: principles and techniques for data scientists. O’Reilly Media, Inc., Sebastopol, CA

12. Gottfredson M, Hirschi T (1990) A General Theory of Crime. Stanford University Press, Stanford, CA

13. Piquero AR, Farrington DP, Blumstein A (2003) The Criminal Career Paradigm. Crime and Justice 30:359–506. https://doi.org/10.1086/652234

14. McGloin JM, Piquero AR (2010) On the Relationship between Co-Offending Network Redundancy and Offending Versatility. Journal of Research in Crime and Delinquency 47:63–90. https://doi.org/10.1177/0022427809348905

15. Braithwaite J (1989) Crime, shame and reintegration. Cambridge University Press, Cambridge, UK

16. Bindler A, Hjalmarsson R (2017) Prisons, recidivism and the age–crime profile. Economics Letters 152:46–49. https://doi.org/10.1016/j.econlet.2017.01.002

17. Cohen LE, Felson M (2010) Social change and crime rate trends: A routine activity approach (1979). In: Classics in environmental criminology. Routledge, pp 203–232

18. Office for National Statistics (2016) Crime in England & Wales, year ending June 2015 - PFA tables. https://www.ons.gov.uk/file?uri=/peoplepopulationandcommunity/crimeandjustice/datasets/policeforceareadatatables/current/previous/v1/04policeforceareadatatablescrimeinenglandandwalesyearendingjune2015_tcm77-419667.xls. Accessed 15 May 2025

19. Håkansson A, Berglund M (2012) Risk factors for criminal recidivism – a prospective follow-up study in prisoners with substance abuse. BMC Psychiatry 12:111. https://doi.org/10.1186/1471-244X-12-111

20. Lu J, Liu A, Dong F, et al (2018) Learning under Concept Drift: A Review. IEEE Trans Knowl Data Eng 1–1. https://doi.org/10.1109/TKDE.2018.2876857

21. Moffitt TE (1993) Adolescence-limited and life-course-persistent antisocial behavior: A developmental taxonomy. Psychological Review 100:674–701. https://doi.org/10.1037/0033-295X.100.4.674
